# Genome-wide identification and molecular evolution of *NAC* gene family in *Dendrobium nobile*

**DOI:** 10.3389/fpls.2023.1232804

**Published:** 2023-08-21

**Authors:** Chun Fu, MingYu Liu

**Affiliations:** Key Laboratory of Sichuan Province for Bamboo Pests Control and Resource Development, Leshan Normal University, Leshan, Sichuan, China

**Keywords:** *Dendrobium nobile*, *NAC* gene family, genome-wide identification, molecular evolution, collinearity analysis

## Abstract

NAC transcription factors are an important genes that regulate plant growth and development, and can regulate functions such as fruit ripening in plants. Based on genome data of *Dendrobium nobile*, the *NAC* gene family was identified and analyzed by bioinformatics methods. In this study, we identified 85 *NAC* genes in *Dendrobium nobile* genome, and systematically analyzed the *NAC* gene family. We found that they were distributed unevenly in the nineteen chromosomes. The amino acid length of *D. nobile NAC* gene family (*DnoNACs*) ranged from 80 to 1065, molecular weight ranged from 22.17 to 119.02 kD, and isoelectric point ranged from 4.61~9.26. Its promoter region contains multiple stress responsive elements, including light responsive, gibberellin-responsive, abscisic acid responsiveness, MeJA-responsiveness and drought-inducibility elements. Phylogenetic analysis indicates that the *D. nobile NAC* gene family is most closely related to *Dendrobium catenatum* and *Dendrobium chrysotoxum*. Analysis of SSR loci indicates that the fraction of mononucleotide repeats was the largest, as was the frequency of A/T. Non-coding RNA analysis showed that these 85 *NAC* genes contain 397 miRNAs. The collinearity analysis shows that 9 collinear locis were found on the chromosomes of *D. nobile* with *Arabidopsis thaliana*, and 75 collinear locis with *D.chrysotoxum.* QRT-PCR experiment under different salt concentration and temperature conditions verified the response mechanism of *DnoNAC* gene family under stress conditions. Most *DnoNAC* genes are sensitive to salt stress and temperature stress. The results of this study provide a reference for further understanding the function of *NAC* gene in *D. nobile*.

## Introduction

*Dendrobium nobile*, a perennial herb of the genus Dendrobium in the Orchidaceae family, is a traditional herbal medicine found primarily in tropical and subtropical Asia (Li et al., 2020; Li L. et al., 2022). According to research, the chemical constituents of *D. nobile* are primarily polysaccharides, alkaloids, amino acids, phenanthrenes, and coumarins, with polysaccharides and alkaloids serving as the foundation of its pharmacological effects and being primarily stored in the stem (Xu Q. et al., 2022). *D. nobile*’s pharmacological effects include antioxidant, antitumor, antihyperglycemic, improving immunity, promoting digestive juice secretion, inhibiting platelet agglutination, and lowering blood lipids (Wang et al., 2010; Li et al., 2011; Liu et al., 2011; Teixeira da Silva and Ng, 2017). Furthermore, the main alkaloid in *D. nobile* is dendrobine, a sesquiterpene alkaloid with significant pharmacological activity that is considered one of the criteria for evaluating *D. nobile* quality (Gong et al., 2022).

Transcription factor (TF) is a protein molecule with a specific structure in eukaryotes that has a regulatory influence on gene transcription (Kadonaga, 1998), and transcription factors in plants can be categorized into *DOF*, *WRKY*, *BZIP*, *NAC*, *MYB*, *ERF*, and other families based on DNA structural domains (Tolosa and Zhang, 2020). *NAC* transcription factors are widespread in terrestrial plants and comprise one of the most extensive families of plant-specific transcription factors involved in plant growth and development, as well as biotic and abiotic stress responses. Its name is derived from three gene fragments, Petunia *NAM*, Arabidopsis *ATAF1/2*, and *CUC2*, and because these three genes encode protein sequences with a highly conserved amino acid sequence at the N-terminus, the initials of these three genes were used to name this structure as the *NAC* structural domain (Aida, 1997; Olsen et al., 2005a). A typical *NAC* family member’s N terminus contains a conserved *NAC*-specific structural domain of about 150 amino acids that not contain the classical helix-turned-helical structure and instead consists of a twisted β-pleated sheet structure surrounded by several helical elements. The functional dimers formed by the *NAC* domain in this structure serve as a structural template for understanding the function of NAC protein at the molecular level (Ernst et al., 2004). Meanwhile, *NAC* is made up of three highly conserved sub-structural domains A, B, and C, as well as two less conserved domains D and E. Sub-structural domain C could be engaged in DNA binding, while sub-structural domain E could be involved in control and synergistic sub-structural domain D binding to DNA throughout plant development (Greve et al., 2003; Diao et al., 2020; Ren et al., 2021). The C-terminus of NAC proteins contains a transcriptional regulatory region with highly variable transcriptional activation or repression activity (Jensen et al., 2007), which is typically characterized by the presence of repetitive sequences of simple amino acids like Ser, Thr, Pro, Glu or acidic amino acid residues (Olsen et al., 2005b).

Although members of the *NAC* family perform some functions similarly, they have distinct roles during various stages and parts of plant growth and development (Liu et al., 2022). For instance, *NAC* can promote the growth of plant reproductive and nutritive organs, participate in the control of secondary plant cell wall production, signal phytohormones, govern the viability of seeds, and control the flowering and senescence of plant tissues. Additionally, it is also involved in plant defense responses in biotic stresses and responses to abiotic stresses, etc (Puranik et al., 2012; Kim et al., 2016; Guo et al., 2018; Diao et al., 2020). The *NAM* gene in the wheat *NAC* transcription factor plays a central role in improving the nutritional value of wheat seeds by regulating the RNA levels of multiple NAM homologs capable of improving protein, zinc and iron content (Uauy et al., 2006). Maize transcription factors *ZmNAM1*, *ZmNAM2*, and *ZmCUC3* are involved in the formation of interstem meristematic tissues (Zimmermann and Werr, 2005). Ectopic expression of NST1 (NAC secondary wall thinking promoting factor 1) or NST2 in *Arabidopsis* can cause ectopic thickening of secondary walls of various aboveground tissues (Mitsuda, 2005). *PtrWND1B* gene expression regulates cell wall thickening in *Populus trichocarpa* (Zhao et al., 2014). The rose *RhNAC*100 transcription factor regulates cell expansion (Pei et al., 2013). The tomato *SlNAP2* transcription factor regulates leaf senescence and fruit yield (Ma et al., 2018). The rice *NAC* transcription factor gene *OsNAC*6 can operate as a transcriptional activator in response to biotic and abiotic stresses, and it can be exploited as a biotechnological tool to improve stress resistance (Nakashima et al., 2007). The ability to express the *NtNAC-R1* gene in the tobacco *NAC* transcription factor impacts lateral root formation and nicotine synthesis (Diao et al., 2020). The wheat *TaNAC2-5A* transcription factor is important in nitrate signaling, and wheat overexpressing *TaNAC2-5A* has better yield and nitrogen buildup (He et al., 2015). The kiwifruit *NAC* transcription factors *AaNAC2*, *AaNAC3*, and *AaNAC4* genes govern fruit terpene synthesis (Nieuwenhuizen et al., 2015), The barley *HvNAC6* transcription factor can increase powdery mildew resistance by modulating ABA (Chen et al., 2013). The norway spruce *PaNAC03* transcription factor affects embryonic development and inhibits flavonoid synthesis (Dalman et al., 2017).

Since the first discovery of *NAC* transcription factors in 1996, researchers have discovered the existence of *NAC* transcription factors in an increasing number of plants, and have made certain progress in their structure, expression characteristics, and biological functions. At the moment, research on *NAC* transcription factors is continuing. Some studies have identified and analyzed members of the *NAC* gene family in plant genomes such as *Arabidopsis thaliana* (Hisako et al., 2004), *Zanthoxylum bungeanum* (Hu et al., 2022), potato (Singh et al., 2013), melon (Wei et al., 2016), sunflower (Bengoa Luoni et al., 2021), *Asparagus officinalis* (Li C. et al., 2022), *Brassica juncea* var. Tumida (He et al., 2020), *Saccharum spontaneum* (Shen et al., 2022) and *Hibiscus hamabo* Sieb (Wang et al., 2022b), but there is no relevant research report on the *NAC* gene family in *D. nobile*’s whole genome. In this study, bioinformatics methods were used to conduct a comprehensive analysis of the physicochemical properties, chromosomal positioning, and conserved motifs of the *D. nobile NAC* gene family (named: *DnoNAC*), and the resulting DnoNAC proteins were compared in multiple sequences to construct an phylogenetic tree to analyze their molecular evolutionary relationships, with the goal of laying the groundwork for future research on the functions of the *D. nobile NAC*.

## Materials and methods

### Data acquisition, plant materials and experimental design

The whole genome sequences, protein sequences and gene annotation files of *D. nobile* were downloaded from NCBI database (https://www.ncbi.nlm.nih.gov) (Xu Q. et al., 2022). The *NAC* family protein sequences of *Arabidopsis thaliana*, *Oryza sativa subsp. japonica*, *Zea mays*, *Populus euphratica*, *Populus trichocarpa*, *Brachypodium distachyon* and *Brachypodium stacei* were downloaded from Plant Transcription Factor Database (http://planttfdb.gao-lab.org/) (Chang and Chow, 2023). The genome sequences, protein sequences and gene annotation files of *Dendrobium catenatum* and *Dendrobium chrysotoxum* were downloaded from the NCBI database to identify the *NAC* gene protein sequences for subsequent bioinformatics analysis. *D. nobile* seedlings are planted in Key Laboratory of Sichuan Province for Bamboo Pests Control and Resource Development of Leshan Normal University from March 10th to May 10th, 2023. The first experimental treatment is as follows: the treatment group treated *D. nobile* seedlings with NaCl solutions at concentrations of 1.0, 3.0, 6.0, and 10.0 g/L for 0 h, 24 h, 48 h, and 72 h, while the control group had a concentration of 0 g/L. The second experimental treatment is as follows: the treatment group is cultivating *D. nobile* seedlings at temperatures of 0 °C, 5 °C, 10 °C, 35 °C, and 40 °C for 0 h, 24 h, 48 h, and 72 h. while the control group had a temperature of 25 °C. The total RNA was extracted from young leaves of the treatment group and the control group. Reverse transcription of purified RNA into cDNA using a reverse transcription kit, and reverse transcribed cDNA was used for qRT-PCR to verify the expression of *NAC* transcription factor family members in *D. nobile.* Plant RNA extraction kit and cDNA reverse transcription kit are purchased from TIANGEN Biotech(Beijing)Co.,Ltd. The qRT-PCR experiments in this study were all completed on fluorescence quantitative PCR instrument (qToWer^3^G) of the Analytick Jena AG. The number of replicates of biological samples in each treatment group and control group is 3, and the number of machine replicates on fluorescence quantitative PCR is 3. All *DnoNAC* gene primers designed by TBtools Batch q-RT-PCR primer design tool used in the qRT-PCR validation experiment in this study are shown in **Supplementary Table 1**. The qRT-PCR primers used in this study were synthesized by Sangon Biotech (Shanghai) Co., Ltd on commission.

### Analysis of identification, chromosomal localization

Analysis of conserved structural domains was performed using the online software SMART (http://smart.embl-heidelberg.de/) (Li et al., 2021). The family genes were identified by combining SMART online software and Pfam, and the selected sequences were simplified using TBtools (Chen et al., 2020). Chromosomal localization analysis of the *NAC* gene family was performed using MapChart software based on the GFF3 files of the genes.

### Analysis of phylogenetic tree, gene structure, conserved motifs, conserved domains, cis-acting elements

*D. nobile* NAC protein sequences were aligned using the Clustalx2.1 program, and the NGPhylogeny.fr online tool (https://NGPhylogeny.fr) was used to build a phylogenetic tree of *D. nobile* NAC proteins, which was subsequently ornamented using the ITOL online website (https://itol.embl.de/) (Puranik et al., 2013; Letunic and Bork, 2021; Wang et al., 2021). The same method was used to construct other phylogenetic trees. The *NAC* gene structure of *D. nobile* was mapped using the GSDS2.0 (http://gsds.cbi.pku.edu.cn/) tool to analyse its exon and intron structures (Hu et al., 2015). MEME(http://meme-suite.org/tools/meme) was used to examine the conservative motifs in the *D. nobile* NAC protein sequences (Bailey et al., 2009). The maximum number of conservative motifs detected was set to ten, and all other parameters were left at their default values (Bailey et al., 2006). Conserved domains in *DonNAC* genes were identified on NCBI’s Conserved Domain Database(https://www.ncbi.nlm.nih.gov/Structure/bwrpsb/bwrpsb.cgi) (Marchler-Bauer et al., 2015). The upstream 2000 bp sequence of *D. nobile NAC* CDS sequences was extracted by TBtools v1.108 software, submitted to PlantCare online website (http://bioinformatics.psb.ugent.be/webtools/plantcare/html/) for prediction of cis-acting element types, positions, and numbers, filtered and counted in Excel 2019, and the obtained cis-acting element position information was used for visualization in TBtools v1.108 (Chen et al., 2020; Hou et al., 2022; Luo et al., 2022).

### Physicochemical properties and subcellular localization analysis

The ExPASY website’s ProtParam tool (http://web.expasy.org/protparam/) was used to analyze the number, molecular weight(MW), isoelectric point information(pI), total number of positively or negatively charged residues, instability index, grand average of hydropathicity(GEAVY), and aliphatic index (Xu B. et al., 2022) of amino acids in DnoNAC proteins. Predicting the subcellular localization of DnoNAC proteins by the WoLF PSORT online website (https://www.genscript.com/wolf-psort.html) (Paul et al., 2007).

### Analysis of transmembrane structural domains, hydrophobicity of amino acids and the secondary and tertiary structures

The transmembrane structure of DnoNAC proteins was analyzed by TMHMMServerv.2.0 software(https://services.healthtech.dtu.dk/services/TMHMM-2.0) (Ding et al., 2019), and the hydrophilicity and hydrophobicity of DnoNAC proteins was predicted by ProtScale (https://web.expasy.org/protscale/) (You et al., 2017). The SOPMA tool (https://npsa-prabi.ibcp.fr/cgi-bin/npsa_automat.pl?page=npsa_sopma.html) was used to predict the secondary structure of DnoNAC proteins (Geourjon and Deléage, 1995). The tertiary structure of this protein family was predicted using SWISS-MODEL online software (https://swissmodel.expasy.org/) (Marco et al., 2014).

### Analysis of codon bias

The necessary metrics, such as codon base composition, relative synonymous codon usage (RSCU), codon adaptation index (CAI), codon bias index (CBI) and effective number of codons (ENC) were obtained using the software CodonW1.4.2. The software TBtools v1.108 was used to create a Heatmap clustering of codons RSCU of *NAC* gene family in *D. nobile* (Wang et al., 2022a).

### Collinearity analysis

The TBtools software Fasta Stats tool was used to process the genome sequence to obtain chromosome length files, the Gene Density Profile tool was used to process the gene structure annotation information to obtain gene density files, and the One Step MCScanX-Super Fast tool was used to compare the *D. nobile* proteins themselves to obtain blast results. The GFF3 Gene Position(Info.) Parse tool was used to acquire all genes’ positions, and the Advanced Circos tool was used to visualize the data (Krzywinski et al., 2009). The similar procedure was used to create *D. nobile* and *Arabidopsis* Circos maps. The simple Ka/Ks Calculator(NG) in Tbtools v1.120 was used to compute the nonsynonymous substitution rate(Ka), synonymous substitution rate(Ks) and selective strength(Ka/Ks) values.

### Prediction of miRNAs targeting *DnoNAC* genes

Potential miRNAs targeting *DnoNAC* genes were predicted using online software psRNATarget (https://www.zhaolab.org/psRNATarget/) with default parameters (Dai and Zhao, 2011).

### Analysis of SSR loci

The SSR locis contained in *DnoNAC* genes were analysed using MISA-web online software (https://webblast.ipk-gatersleben.de/misa/) with default parameters (Beier et al., 2017).

## Results

### Chromosome mapping analysis

85 *NAC* genes were identified in *D. nobile* genome by Pfam model and SMART search, which were called *DnoNAC01*~*DnoNAC85* based on the gene descriptions. *DnoNAC* genes were not found on chromosome 8 and were dispersed irregularly on the other chromosomes, according to the findings of chromosomal localization analysis using Mapchart 2.32. Chromosome 19 has the most *DnoNAC* genes, with 11 genes, named *DnoNAC01* to *DnoNAC11*. Chr2, Chr7 and Chr11 have the fewest *DnoNAC* genes, with only two family members (**Supplementary Figure 1**).

### Phylogenetic analysis

**Figure 1:**
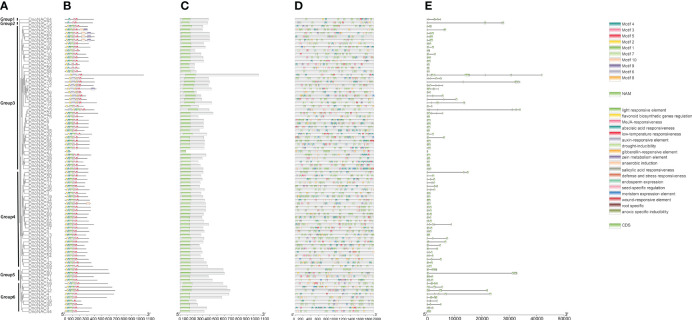
Phylogenetic tree, conserved motif, conserved domain, cis-acting elements and gene structure of *NAC* family in *Dendrobium nobile*. **(A)** phylogenetic tree of *DnoNAC* genes. **(B)** conserved motifs of *DnoNAC* proteins. **(C)** conserved domains of *DnoNAC* proteins. **(D)** cis-acting elements of *DnoNAC* genes’ promoter. **(E)** gene structure of *DnoNAC* genes.

85 members of the family was divided into 6 subfamilies based on significant amino acid sequence similarity and evolutionary closeness(designated Group1 to Group6). The *DnoNAC*s in the 6 subfamilies vary greatly, with Group3 having the most family members, accounting for 49.41% of the total number of family members, and Groups 1 and 2 having the fewest, accounting for 1.18% of the total number of family members. Group1 and Group2 are the most primitive of the entire family, while Group6 is the fastest evolving (**Figure 1A**).

**Figure 2:**
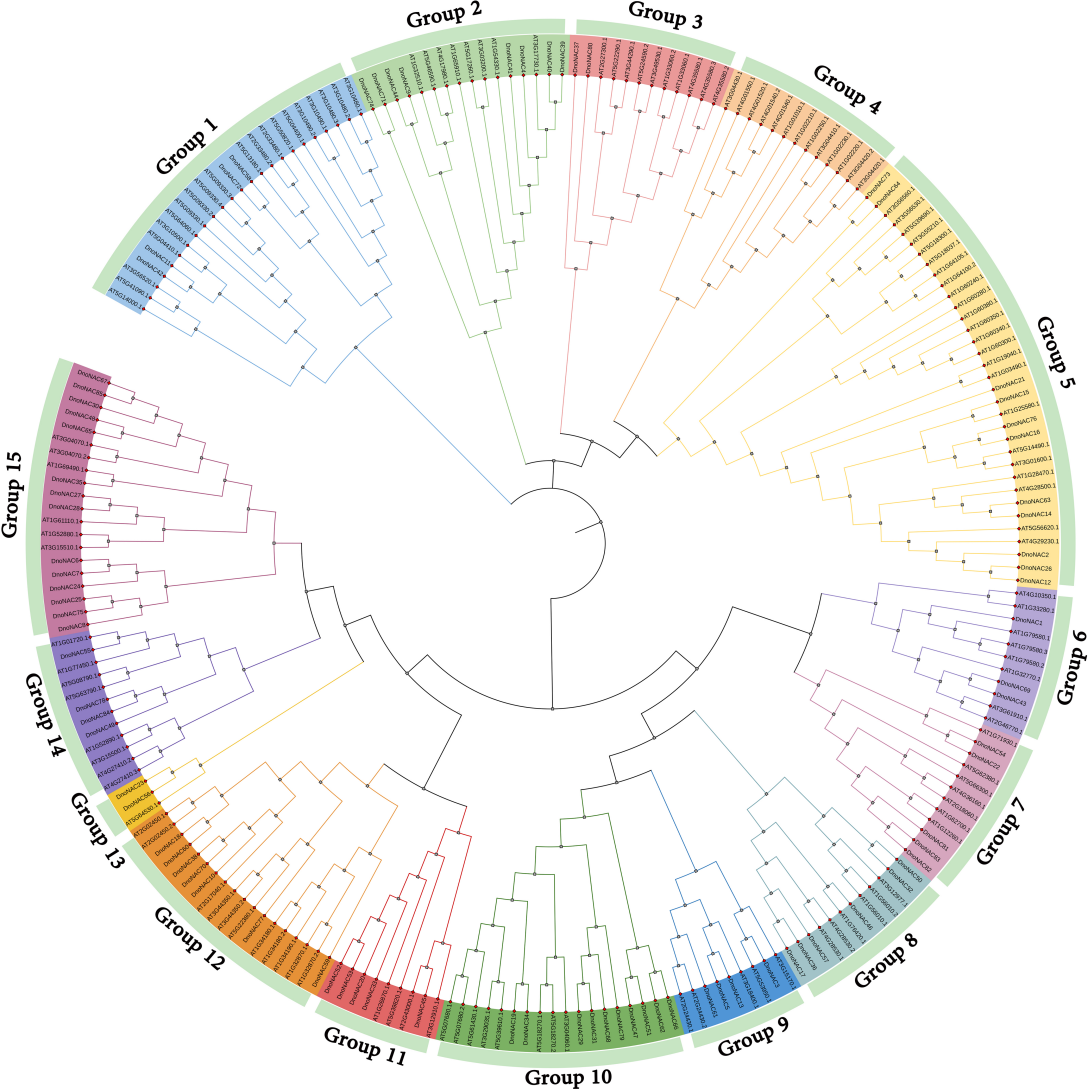
Phylogenetic tree of *NAC* gene family in *Dendrobium nobile* and *Arabidopsis thaliana*.

The phylogenetic tree of *NAC* gene family in *D. nobile* and *Arabidopsis thaliana* were separated into 15 subclades (designated Group1 to Group15), with 85 *DnoNAC* genes dispersed on 14 subclades excluding Group4. Group15 contains the most members at 14 subclades, followed by Group5 with 11 and Group9 with 10 *DnoNAC* members, Group6 contains 3 *DnoNAC* members, Group3 and Group13 contain the least number of members at 2. In general, Group 1 members are the most primitive, while Group 15 members are the most rapidly developing (**Figure 2**).

**Figure 3:**
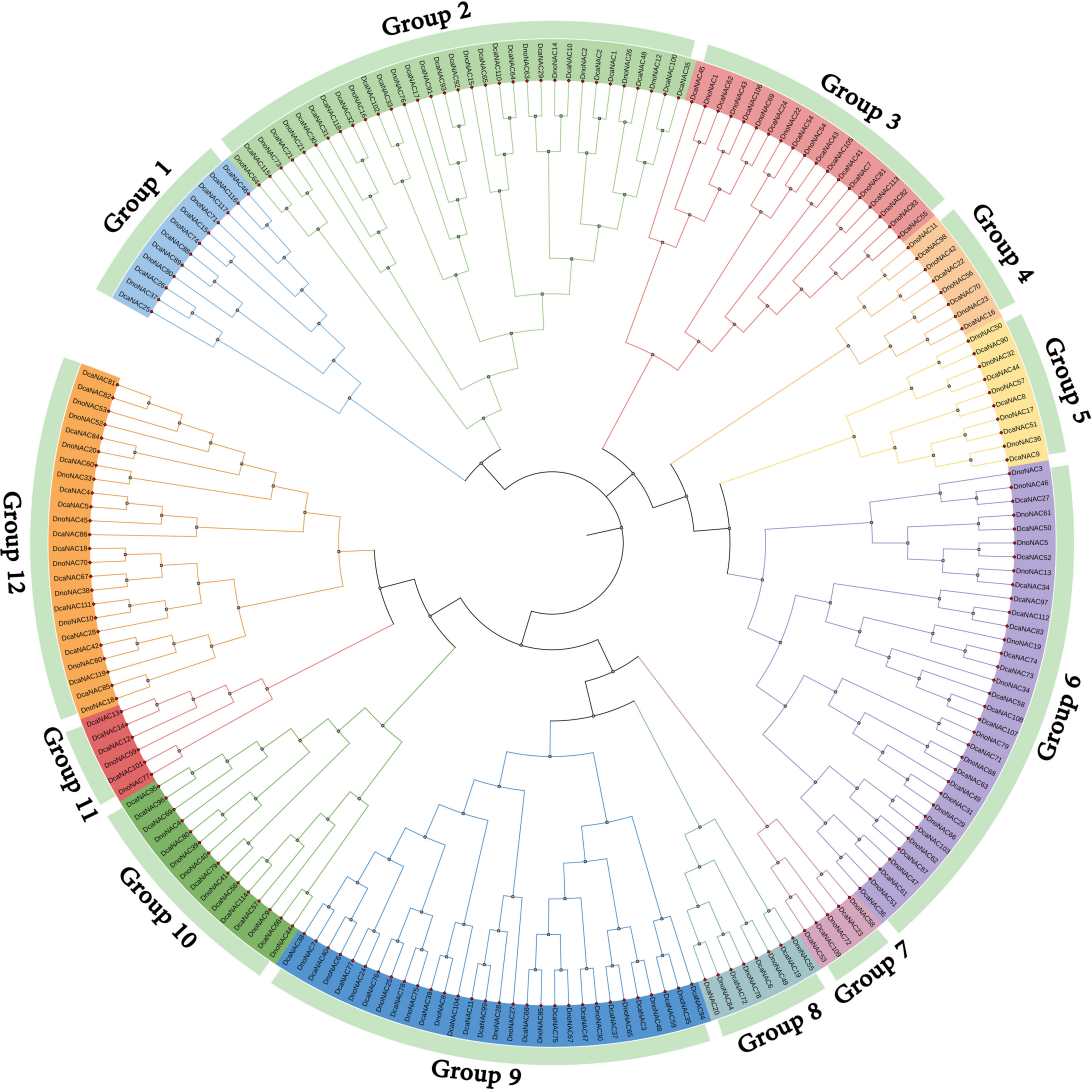
Phylogenetic tree of *NAC* gene family in *Dendrobium nobile* and *Dendrobium catenatum*.

The phylogenetic tree of *NAC* gene family in *D. nobile* and *Dendrobium catenatum* were separated into 12 subclades (designated Group1 to Group12), each containing 2 to 15 members. Group6 contains the most members at 15, followed by Group9 with 14 and Group2 with 11 *DnoNAC* members, Group5 contains 5 *DnoNAC* members, Group7 and Group11 contain the least number of members at 2. In general, Group 1 members are the most primitive, while Group 12 members are the most rapidly developing (**Figure 3**).

**Figure 4:**
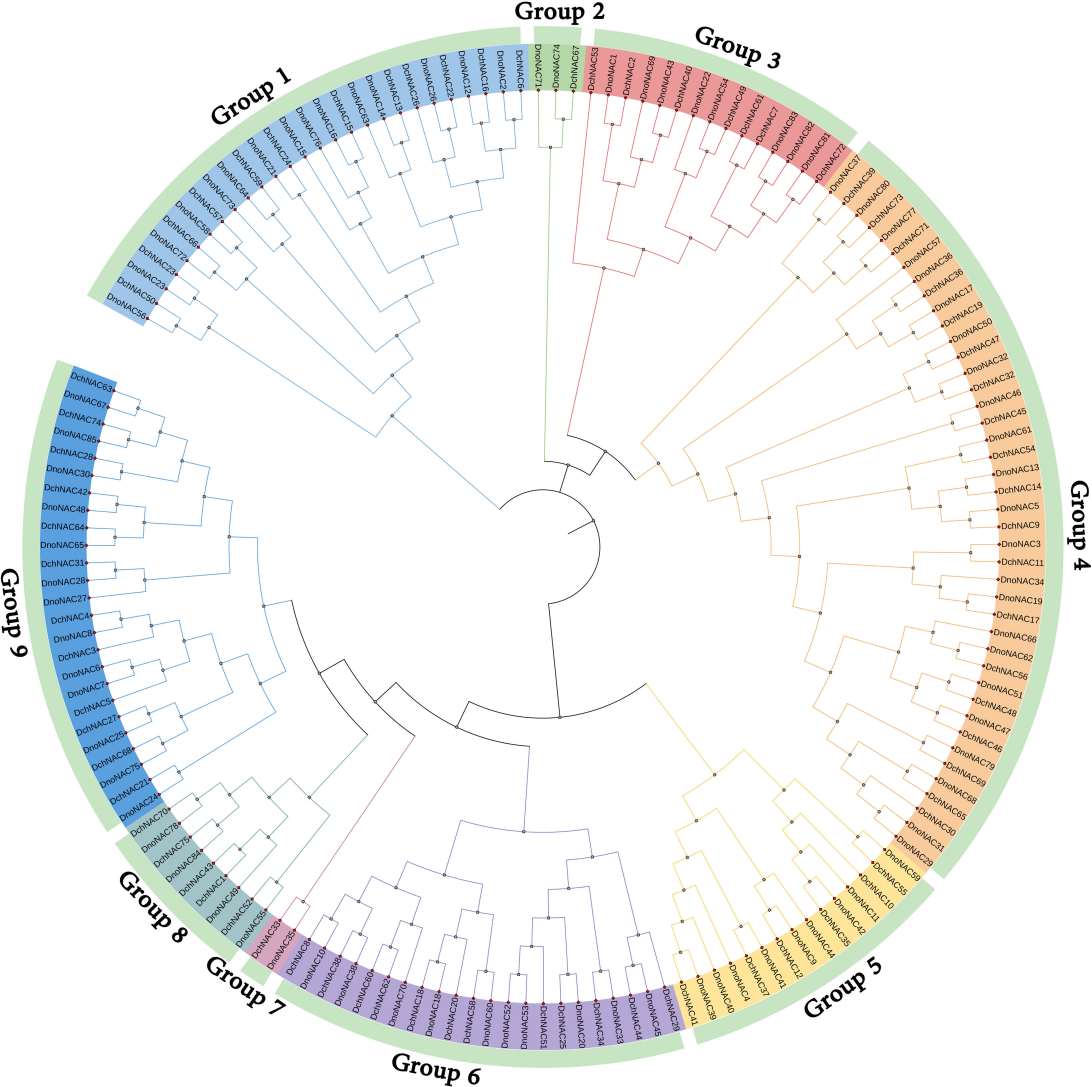
Phylogenetic tree of *NAC* gene family in *Dendrobium nobile* and *Dendrobium chrysotoxum*.

The phylogenetic tree of *NAC* gene family in *D. nobile* and *Dendrobium chrysotoxum* were separated into 9 subclades (designated Group1 to Group9), each containing 1 to 23 members. Group4 contains the most members at 23, followed by Group1 with 15 and Group9 with 13 *DnoNAC* members, Group2 contains 2 *DnoNAC* members, and Group7 contains the least number of members at 1. In general, Group 1 members are the most primitive, while Group 9 members are the most rapidly developing (**Figure 4**).

**Figure 5:**
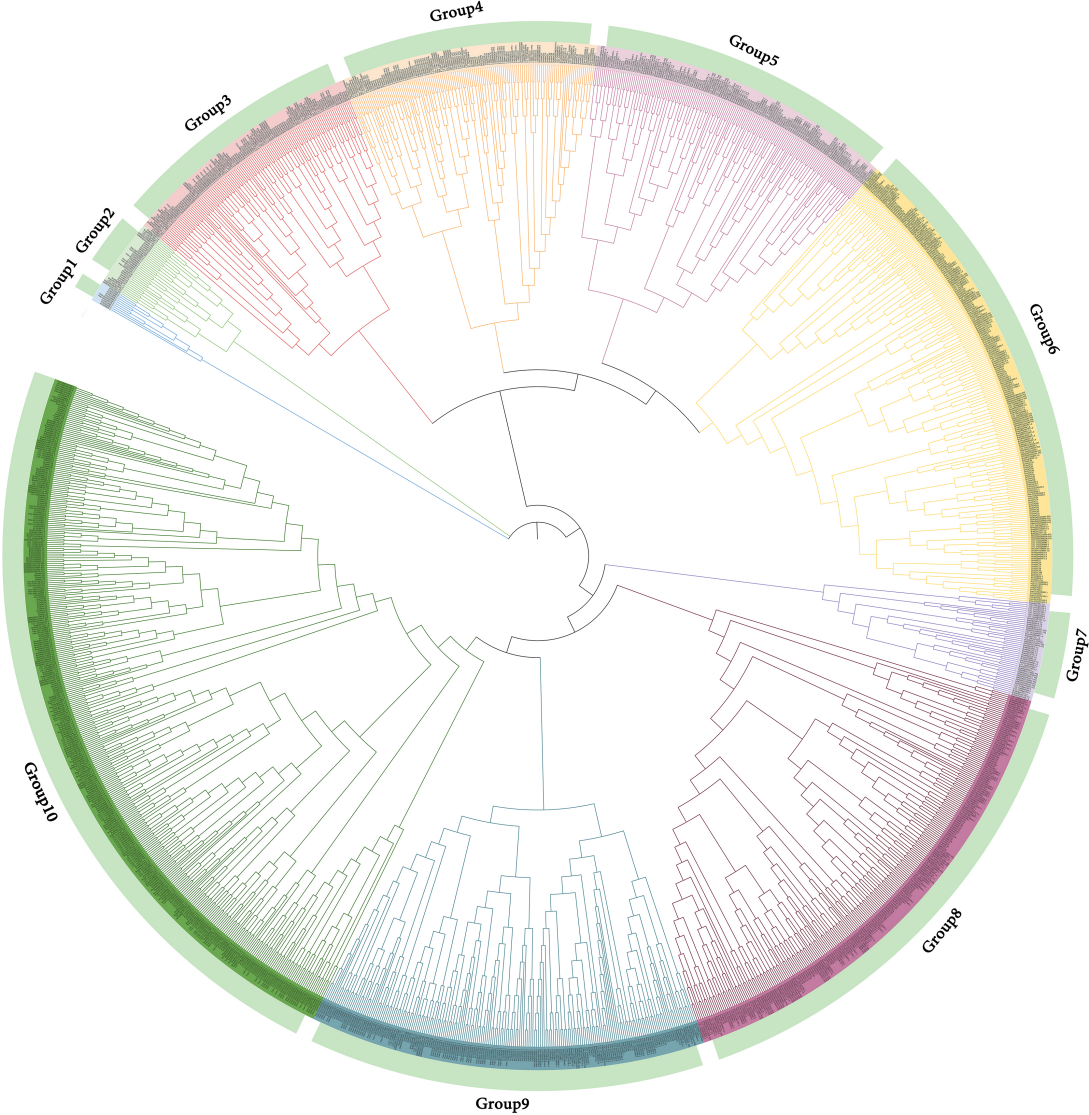
Phylogenetic tree of *NAC* gene family in *Dendrobium nobile* and other species.

The phylogenetic tree of *NAC* gene family in *D. nobile*, *Arabidopsis thaliana*, *Dendrobium catenatum*, *Dendrobium chrysotoxum*, *Oryza sativa subsp. Japonica*, *Zea mays*, *Populus euphratica*, *Populus trichocarpa*, *Brachypodium distachyon* and *Brachypodium stacei* were separated into 10 subclades (designated Group1 to Group10), with 85 *DnoNAC* genes dispersed on 10 subclades excluding Group1 and Group7. Group6 and Group8 contain the most members at 20, followed by Group3 with 10 and Group10 with 9 *DnoNAC* members. Combined with the distribution of these genes on the phylogenetic tree, it is known that the *D. nobile NAC* gene family is most closely related to *Dendrobium catenatum* and *Dendrobium chrysotoxum*, followed by *Brachypodium distachyon*, and *Zea mays* is the most distant (**Figure 5**).

### Gene structure analysis

The gene structural map of the *D. nobile* revealed that all 85 *DnoNAC* genes comprised introns and exons. The number of exons ranged from 2 to 13, with 60 genes having 3 exons, accounting for 70.59% of the total. The family member with the fewest exons was *DnoNAC27*, and the family member with the most exons was *DnoNAC21* (**Figure 1E**).

### Conservative motifs and conserved domain analysis

The conservative motifs of the 85 NAC protein sequences of *D. nobile* were analysed by using the online tool MEME. According to the findings, the lengths of the ten motifs ranged from 11 to 50 amino acids, 85 members included motifs in varied numbers, ranging from 2 to 8, with 83 members containing motif 2, showing that this motif 2 is more conserved in *D. nobile* NAC proteins (**Supplementary Figure 2**). Most *DnoNAC*s’ motifs are placed in the following order, motif2, motif4, motif1, motif7, motif3, motif5 were found in most subfamilies, with the highest frequency, and are associated with the NAM structural domain, which corresponds to the five conserved substructures A to E of the N-end part of the *DnoNAC* transcription factor. Furthermore, there are a few *DnoNAC*s with varying numbers and types of motifs, presumably due to mutations during evolution, which may be related to the various functions performed in the organism. (**Figures 1B, C**).

### Cis-acting elements analysis

**Figure 6:**
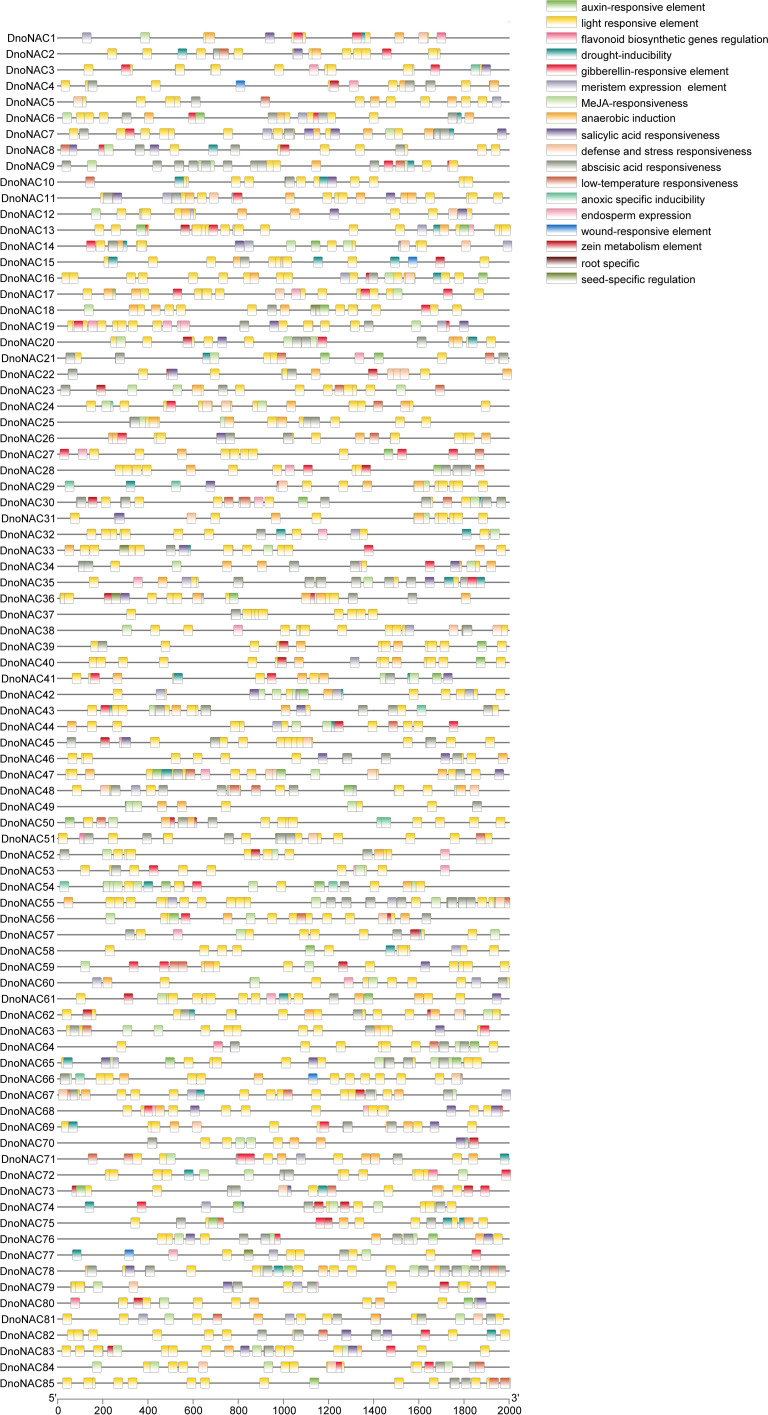
Cis-acting elements of *NAC* gene family in *Dendrobium nobile*.

The screening of 18 cis-acting elements was accomplished by analyzing 2000 bp regions upstream of the promoters of *DnoNAC* gene family members (**Figure 1D**). Light responsive elements were found in 85 *DnoNAC* family members, with the promoter region of *DnoNAC55* having the highest (31 light responsive elements)elements. Abscisic acid responsiveness element was found in 71 family members, with the promoter region of *DnoNAC78* having the highest (13 light responsive elements). MeJA-responsiveness element was found in 67 family members, with the promoter region of *DnoNAC54* having the highest (18 light responsive elements). Anaerobic induction element was found in *64* family members, with the promoter region of *DnoNAC11* having the highest (5 light responsive elements). Gibberellin-responsive element was found in 43 family members, with the promoter region of *DnoNAC17* and *DnoNAC27* having the highest (3 light responsive elements). Drought-inducibility element was found in 40 family members, with the promoter region of *DnoNAC15* having the highest (3 light responsive elements). Salicylic acid responsiveness element was found in 40 family members, with the promoter region of *DnoNAC7* and *DnoNAC68* having the highest (3 light responsive elements). Auxin-responsive element was found in 36 family members, with the promoter region of *DnoNAC13*, *DnoNAC21*, *DnoNAC30*, *DnoNAC47*, *DnoNAC54* and *DnoNAC65* having the highest (2 light responsive elements). Zein metabolism element was found in 36 family members, with the promoter region of *DnoNAC73* having the highest (3 light responsive elements). Root specific element was only found in 1 family members, with the promoter region of *DnoNAC10*. Seed-specific regulation element was found in 36 family members, with the promoter region of *DnoNAC18* having the highest (3 light responsive elements). Low-temperature responsiveness element was found in 31 family members, with the promoter region of *DnoNAC30* having the highest (4 light responsive elements). Meristem expression element was found in 30 family members, with the promoter region of *DnoNAC42* having the highest (3 light responsive elements). Defense and stress responsiveness element was found in 27 family members, with the promoter region of *DnoNAC22* and *DnoNAC47* having the highest (3 light responsive elements). Endosperm expression element was found in 23 family members, with the promoter region of *DnoNAC19* having the highest (4 light responsive elements). Anoxic specific inducibility element was found in 9 family members, with the promoter region of *DnoNAC29*, *DnoNAC50*, *DnoNAC54* and *DnoNAC55* having the highest (2 light responsive elements). Flavonoid biosynthetic genes regulation element was found in 6 family members, with the promoter region of *DnoNAC1*, *DnoNAC4*, *DnoNAC51*, *DnoNAC64*, *DnoNAC72* and *DnoNAC80* having the highest (1 light responsive elements). Wound-responsive element was found in 5 family members, with the promoter region of *DnoNAC4*, *DnoNAC15*, *DnoNAC66*, *DnoNAC74* and *DnoNAC77* having the highest (1 light responsive elements). Members of the *DnoNAC* family contain a variety of cis-elements, and it is anticipated that these family members serve critical roles in *D. nobile’*s response to environmental stress and hormone control (**Figure 6**).

The analysis of transcription factor binding sites revealed that all *DnoNAC*s exhibited a dense distribution of TFBSs in the promoter region. Based on the number of binding sites, four fundamental TFBSs were chosen for demonstration, with *ERF* having the most at 12,231 (**Supplementary Figure 3**), followed by *BBR-BPC* at 4,231 (**Supplementary Figure 4**), *MYB* at 3,290 (**Supplementary Figure 5**), and *NAC* having the fewest at 3,288 (**Supplementary Figure 6**). Three *DnoNAC*s(*DnoNAC14*, *DnoNAC49*, and *DnoNAC71*) were expected to have the highest number of TFBSs. The prediction of TFBSs provides a basis for further identification and validation of target genes.

### Analysis of the physicochemical properties and subcellular localization

The *D. nobile* NAC protein sequences were submitted to the ProtParam online program to calculate its length, Mw, pI, and other properties. The results showed that the total number of amino acids of *D. nobile NAC* protein family members ranged from 80 aa to 1065 aa, with DnoNAC21 having the highest number of amino acids (1065 aa) and DnoNAC27 having the lowest number of amino acids (80 aa). The average length of amino acid is 349, and the molecular weight ranges from 22.17 to 119.02 kD. Its pI ranges from 4.61 to 9.26, covering a wide range, with 29 members of its family having a theoretical isoelectric point greater than 7 and the remaining family members having a theoretical isoelectric point less than 7. As a result, the majority of members are acidic proteins. The majority of the 85 family members have instability coefficients above 40%, with only 16 members having instability index between 27.53% and 39.88%, the range of Aliphatic index of the family members between 51.78 and 86.85 indicates the wide variation in the thermal stability of the family proteins. The grand average of hydropathicity of the family members were all negative, with the greatest being -0.352 for member *DnoNAC44* and the lowest being -0.918 for member DnoNAC14. 54 members have a greater number of negatively charged residues than positively charged residues, indicating a negative charge, 8 members have an equal number of negatively charged residues as positively charged residues, indicating electric neutrality, and the remaining 23 members have a greater number of positively charged residues than negatively charged residues, indicating a positive charge.

The subcellular localization results of the DnoNAC protein family shows that 70 DnoNAC members were found in the nucleus, six members are found in chloroplast (DnoNAC11, DnoNAC12, DnoNAC23, DnoNAC30, and DnoNAC69, DnoNAC80), three members were found in cytoplasm (DnoNAC26, DnoNAC74, DnoNAC78). three members were found in the mitochondria, including DnoNAC55, DnoNAC76 and DnoNAC83, three members were found in the peroxisome, including DnoNAC16, DnoNAC17 and DnoNAC43 (**Table 1**).

**Table 1:** Physicochemical properties of NAC proteins from *Dendrobium nobile*.

Protein	ID	Amino acid number/aa	MW/kDa	PI	Instability index	Aliphatic index	GEAVY	Asp+Glu	Arg+Lys	subcellular localization
DnoNAC01	KAI0487980.1	319	36.51	7.15	40.55	68.21	-0.633	35	35	Nuclear
DnoNAC02	KAI0488077.1	424	47.41	6.65	53.62	60.31	-0.815	57	53	Nuclear
DnoNAC03	KAI0488151.1	284	32.02	8.70	37.70	60.49	-0.407	31	36	Nuclear
DnoNAC04	KAI0488161.1	661	75.69	5.97	47.13	65.52	-0.665	88	74	Nuclear
DnoNAC05	KAI0488616.1	326	36.89	8.48	27.53	59.20	-0.637	31	34	Nuclear
DnoNAC06	KAI0488954.1	320	36.32	6.48	48.19	68.25	-0.578	39	38	Nuclear
DnoNAC07	KAI0488955.1	318	35.98	5.59	47.65	68.40	-0.484	41	36	Nuclear
DnoNAC08	KAI0488956.1	316	35.51	6.38	48.48	68.86	-0.543	38	35	Nuclear
DnoNAC09	KAI0489002.1	361	41.00	5.67	50.75	69.06	-0.456	47	39	Nuclear
DnoNAC10	KAI0489044.1	284	32.78	6.49	60.75	67.01	-0.734	41	40	Nuclear
DnoNAC11	KAI0489316.1	637	70.44	4.61	46.35	79.62	-0.444	98	57	Chloroplast
DnoNAC12	KAI0491902.1	384	43.00	8.09	48.33	68.31	-0.695	47	49	Chloroplast
DnoNAC13	KAI0492501.1	283	32.32	7.05	37.32	59.26	-0.710	34	34	Nuclear
DnoNAC14	KAI0492607.1	283	32.48	8.59	46.56	62.65	-0.918	37	41	Nuclear
DnoNAC15	KAI0492745.1	428	48.21	5.32	43.85	66.29	-0.737	71	50	Nuclear
DnoNAC16	KAI0492769.1	387	43.82	5.65	49.81	71.06	-0.675	55	43	Peroxisome
DnoNAC17	KAI0493482.1	272	31.74	5.93	40.72	60.88	-0.756	37	32	Peroxisome
DnoNAC18	KAI0494268.1	321	36.73	7.06	54.14	68.07	-0.731	41	41	Nuclear
DnoNAC19	KAI0494619.1	310	35.46	6.22	40.65	56.03	-0.679	44	41	Nuclear
DnoNAC20	KAI0495450.1	398	45.03	6.62	46.62	61.56	-0.523	46	45	Nuclear
DnoNAC21	KAI0495750.1	1065	119.02	5.16	48.88	86.85	-0.391	156	116	Nuclear
DnoNAC22	KAI0496083.1	300	34.61	5.23	45.00	63.03	-0.794	46	34	Nuclear
DnoNAC23	KAI0496128.1	198	22.46	6.16	36.85	63.03	-0.441	27	24	Chloroplast
DnoNAC24	KAI0496717.1	446	50.62	8.88	39.88	69.06	-0.609	54	61	Nuclear
DnoNAC25	KAI0496718.1	395	45.20	5.09	37.77	57.01	-0.788	58	37	Nuclear
DnoNAC26	KAI0496808.1	222	24.91	8.49	31.88	73.33	-0.649	28	31	Cytoplasmic
DnoNAC27	KAI0497749.1	80	93.34	5.21	44.91	63.38	-0.659	13	8	Nuclear
DnoNAC28	KAI0497750.1	352	37.80	8.62	48.61	64.69	-0.538	33	37	Nuclear
DnoNAC29	KAI0498156.1	348	39.24	6.60	40.50	65.83	-0.551	42	40	Nuclear
DnoNAC30	KAI0498159.1	363	41.35	7.74	48.45	72.56	-0.605	39	40	Chloroplast
DnoNAC31	KAI0498162.1	348	39.25	6.37	42.04	63.85	-0.584	43	40	Nuclear
DnoNAC32	KAI0499000.1	313	35.30	7.64	46.43	63.90	-0.500	35	36	Nuclear
DnoNAC33	KAI0499225.1	304	34.93	8.62	58.87	70.62	-0.599	35	39	Nuclear
DnoNAC34	KAI0499251.1	335	37.94	6.15	28.53	64.36	-0.541	44	41	Nuclear
DnoNAC35	KAI0499252.1	262	29.96	5.53	56.79	67.02	-0.658	37	29	Nuclear
DnoNAC36	KAI0499741.1	264	30.57	5.94	50.16	60.91	-0.699	35	30	Nuclear
DnoNAC37	KAI0499901.1	585	64.71	4.75	36.92	76.56	-0.463	88	60	Nuclear
DnoNAC38	KAI0500092.1	313	36.01	6.99	56.96	65.14	-0.735	40	40	Nuclear
DnoNAC39	KAI0500140.1	218	25.14	4.69	51.37	60.37	-0.783	40	26	Nuclear
DnoNAC40	KAI0500144.1	236	27.22	4.94	49.34	62.37	-0.750	40	30	Nuclear
DnoNAC41	KAI0500145.1	566	63.73	6.09	45.88	68.55	-0.544	78	68	Nuclear
DnoNAC42	KAI0500568.1	677	75.17	4.77	39.38	71.17	-0.465	98	59	Nuclear
DnoNAC43	KAI0500751.1	355	39.69	6.79	43.50	64.85	-0.610	43	41	Peroxisome
DnoNAC44	KAI0500845.1	327	37.34	5.64	53.01	72.20	-0.352	43	37	Nuclear
DnoNAC45	KAI0501407.1	308	35.87	6.49	45.85	62.31	-0.770	43	42	Nuclear
DnoNAC46	KAI0501644.1	331	37.05	6.27	51.87	68.37	-0.417	35	30	Nuclear
DnoNAC47	KAI0501795.1	311	35.94	5.65	43.90	64.28	-0.677	44	36	Nuclear
DnoNAC48	KAI0501797.1	331	37.62	8.92	52.30	68.37	-0.692	36	41	Nuclear
DnoNAC49	KAI0501798.1	300	34.26	5.49	44.02	63.80	-0.739	46	41	Nuclear
DnoNAC50	KAI0503521.1	283	32.05	6.89	52.18	64.45	-0.442	35	35	Nuclear
DnoNAC51	KAI0504105.1	297	34.3	9.1	40.9	69.6	-0.701	35	44	Nuclear
DnoNAC52	KAI0507012.1	398	45.24	6.67	44.59	61.78	-0.544	46	45	Nuclear
DnoNAC53	KAI0507014.1	398	45.25	6.67	46.18	61.53	-0.551	46	45	Nuclear
DnoNAC54	KAI0507790.1	275	31.75	5.89	47.05	60.29	-0.748	38	32	Nuclear
DnoNAC55	KAI0507839.1	313	35.12	9.00	45.83	69.62	-0.521	35	42	Mitochondria
DnoNAC56	KAI0507891.1	194	22.17	5.18	31.32	62.22	-0.522	28	19	Nuclear
DnoNAC57	KAI0510199.1	302	34.49	5.73	40.42	67.12	-0.581	44	34	Nuclear
DnoNAC58	KAI0510561.1	219	25.06	9.26	54.10	70.73	-0.664	26	33	Nuclear
DnoNAC59	KAI0510647.1	580	64.81	4.85	43.41	67.59	-0.484	83	51	Nuclear
DnoNAC60	KAI0510707.1	342	39.41	7.21	43.8	72.98	-0.69	45	45	Nuclear
DnoNAC61	KAI0511069.1	324	36.50	8.76	43.47	63.24	-0.673	34	39	Nuclear
DnoNAC62	KAI0511981.1	321	36.83	5.32	33.45	55.23	-0.795	47	37	Nuclear
DnoNAC63	KAI0515580.1	293	32.69	8.6	30.8	62.83	-0.801	35	39	Nuclear
DnoNAC64	KAI0516465.1	386	43.79	4.74	48.73	60.08	-0.722	58	34	Nuclear
DnoNAC65	KAI0519417.1	326	37.35	9.03	42.23	67.06	-0.728	32	39	Nuclear
DnoNAC66	KAI0519420.1	388	43.68	5.87	35.18	63.35	-0.552	50	42	Nuclear
DnoNAC67	KAI0520039.1	323	37.00	7.81	51.06	67.37	-0.754	35	36	Nuclear
DnoNAC68	KAI0520048.1	318	35.85	8.4	38.93	59.81	-0.589	35	38	Nuclear
DnoNAC69	KAI0520209.1	377	41.95	6.60	44.36	69.36	-0.529	43	40	Chloroplast
DnoNAC70	KAI0520324.1	319	36.29	6.06	62.5	73.76	-0.569	41	39	Nuclear
DnoNAC71	KAI0522392.1	386	43.92	6.34	50.39	70.23	-0.592	50	47	Nuclear
DnoNAC72	KAI0522705.1	250	27.67	9.21	48.57	67.08	-0.384	25	34	Nuclear
DnoNAC73	KAI0523433.1	375	41.7	4.87	58.56	59.31	-0.83	56	36	Nuclear
DnoNAC74	KAI0523954.1	437	50.08	5.87	50.01	78.28	-0.566	66	57	Cytoplasmic
DnoNAC75	KAI0524416.1	247	28.29	7.01	54.54	74.62	-0.529	30	30	Nuclear
DnoNAC76	KAI0524531.1	400	45.22	5.48	54.67	70.45	-0.705	65	49	Mitochondria
DnoNAC77	KAI0527000.1	202	22.65	8.99	59.38	51.78	-0.660	22	26	Nuclear
DnoNAC78	KAI0527573.1	246	28.33	6.01	48.2	69.11	-0.796	39	36	Cytoplasmic
DnoNAC79	KAI0527576.1	345	39.44	6.16	36.91	65.25	-0.587	44	40	Nuclear
DnoNAC80	KAI0529092.1	601	67.36	5.77	51.29	74.96	-0.508	81	71	Chloroplast
DnoNAC81	KAI0529148.1	313	36.61	6.49	44.32	62.91	-0.898	44	40	Nuclear
DnoNAC82	KAI0530635.1	262	31.07	8.48	44.65	75.46	-0.806	37	40	Nuclear
DnoNAC83	KAI0530639.1	286	33.80	7.19	44.58	77.66	-0.733	40	40	Mitochondria
DnoNAC84	KAI0531374.1	274	31.32	5.43	43.74	63.39	-0.695	42	37	Nuclear
DnoNAC85	KAI0531375.1	333	37.71	7.64	47.57	69.76	-0.642	37	38	Nuclear

### Analysis of transmembrane structure prediction and hydrophobicity of amino acids

The analysis of transmembrane structural domains of the protein members encoded by the DnoNAC protein family revealed that only DnoNAC11, DnoNAC37, DnoNAC42, DnoNAC59, DnoNAC74, and DnoNAC80 of the 85 DnoNAC family members have transmembrane structural domains, implying that the family proteins are transmembrane proteins(**Table 2**, **Supplementary Figure 7**).

**Table 2:** Transmembrane structure prediction of NAC proteins from *Dendrobium nobile*.

Protein name	Number of transmembrane	TMhelix
DnoNAC11	1	604(Gly)-626(Leu)
DnoNAC37	1	560(Phe)-F582(Phe)
DnoNAC42	1	648(Gly)-670(Phe)
DnoNAC59	1	545(Tyr)-567(Phe)
DnoNAC74	1	403(Tyr)-422(Phe)
DnoNAC80	1	579(Leu)-598(Val)

The greatest hydrophobicity value of the DnoNAC protein members varied from 0.833 to 3.600, whereas the maximum hydrophilicity value ranged from -1.911 to -3.856. Based on the rule that the lower the amino acid value, the more hydrophilic, and the higher the amino acid value, the more hydrophobic. According to the trend of lower amino acid values being more hydrophilic and higher amino acid values being more hydrophobic, alanine at position 586 of DnoNAC80 has the most hydrophobic value while glutamic acid at position 160 of DnoNAC17 has the greatest hydrophilic value. The hydrophilic peaks are more frequent and dense than the hydrophobic peaks. As a result, the DnoNAC protein is relatively hydrophilic (**Supplementary Table 2**, **Supplementary Figure 8**).

### Analysis of the secondary and tertiary structures

The secondary structure of the DnoNAC proteins were examined using SOPMA online program, and the results revealed that the *NAC* gene family’s secondary structure is mostly made of α-helix, extended strand, β-turn and random coil. Among them, the highest proportion of random coil is 38.59% (DnoNAC21) to 73.62% (DnoNAC20), the proportion of alpha helix is 12.84% (DnoNAC34) to 33.90% (DnoNAC21), the proportion of extended strand structure is 6.28% (DnoNAC20) to 21.59 (DnoNAC36), and the lowest proportion of beta turn is 1.25% (DnoNAC27) ~ 8.74% (DnoNAC24). Except for DnoNAC14, DnoNAC26, DnoNAC27, DnoNAC34, DnoNAC36, DnoNAC40, DnoNAC46, and DnoNAC54, whose secondary structure ratios were random coil > extended strand ≥ alpha helix > beta turn, the remaining 74 members’ secondary structure ratios were random coil > alpha helix > extended strand > beta turn, indicating that side chain interactions have a significant impact on DnoNAC proteins(**Supplementary Table 3**, **Supplementary Figure 9**).

The SWISS-MODEL online software was used to predict the tertiary structure of the protein members encoded by the *D. nobile NAC* gene family, and the family was divided into six groups based on the similarity of the projected outcomes, marked as A, B, C, D, E and F. Group A has 63 members, followed by Group C, which has 13 members, Group B has 6 family members(DnoNAC3, DnoNAC5, DnoNAC44, DnoNAC51, DnoNAC61, and DnoNAC79). Groups D, E, and F each have one family member(DnoNAC14, DnoNAC21, and DnoNAC27). In conclusion, the *D. nobile* NAC family members have a higher fraction of random coil, whereas other structures are scattered across the protein structure, which is consistent with the secondary structure predictions (**Supplementary Figure 10**).

### Analysis of codon usage bias

The average content of the third base of the codon was T3s > A3s > C3s > G3s. The average GC content(GC) of the codon was 0.29-0.58, with a mean value of 0.40. GC of silent 3rd codon posit(GC3s) was 0.27-0.58, with a mean value of 0.38. According to an analysis of codon-related parameters of the *D. nobile NAC* gene family. Both the GC content and the GC3 mean values are less than 50%, showing that AU is utilized more frequently than GC in the codon of this family member’s coding sequence. The codon adaptiation index(CAI) varied from 0.11 to 0.22, with a mean value of 0.17, showing that the *D. nobile NAC* gene family has a low preference for codon selection. The frequency of optimal codons(Fop) varied from 0.31 to 0.50, with a mean value of 0.37. The codon bias index(CBI) ranged from 0.16 to 0.15, with a mean value of -0.07. The effective number of codons(ENc) varied from 45.56 to 59.70, with a mean value of 53.32, indicating that family members are more varied from one another, have relatively modest levels of expression, and show a low preference for codons when encoding amino acids. The number of synonymous codons (L_sym) ranged from 97 to 12736, with a mean of 1616.54. Total number of amino acids (L_aa) ranged from 100 to 13179, with a mean of 1674.25, and the aromaticity of protein (Aromo) ranged from 0.06 to 0.19, with a mean of 0.12 (**Supplementary Table 4**).

### Analysis of relative synonymous codon usage

There are 27 high-use codons(RSCU>1), 13 codons of which end in U, 11 end in A, 2 end in G, and 1 end in C.(with the exception of the termination codons UAA, UGA and UAG, as well as the initiation codons AUG and UGG). In 32 low-usage codons, 16 end in C, 10 end in G, 3 end in A and 3 end in U. This indicates that the preference for high-use codons ends in U and the preference for low-use codons ends in C. In addition, the RSCU value for AGA is greater than 2, revealing that *D. nobile NAC* family members have a strong preference for this codon (**Table 3**)

**Table 3:** Relative codon usage of the NAC gene family in *Dendrobium nobile*.

Amino acid	Codon	number	RSCU	Amino acid	Codon	number	RSCU
Phe(F)	UUU	76	1.21	Tyr(Y)	UAU	46	1.33
	UUC	41	0.79		UAC	20	0.68
Leu(L)	UUA	48	1.29	TER	UAA	47	1.26
	UUG	40	1.18		UAG	24	0.59
	CUU	39	1.27	His(H)	CAU	34	1.33
	CUC	24	0.8		CAC	18	0.66
	CUA	26	0.71	Gln(Q)	CAA	38	1.26
	CUG	18	0.75		CAG	17	0.74
ILE(I)	AUU	59	1.21	Asn(N)	AAU	60	1.32
	AUC	33	0.84		AAC	29	0.68
	AUA	49	0.93	Lys(K)	AAA	74	1.22
Met(M)	AUG	34	0.99		AAG	38	0.78
Val(V)	GUU	29	1.42	Asp(D)	GAU	30	1.4
	GUC	14	0.73		GAC	14	0.6
	GUA	20	1.02	Glu(E)	GAA	39	1.21
	GUG	16	0.81		GAG	24	0.79
Ser(S)	UCU	41	1.42	Cys(C)	UGU	29	1.03
	UCC	26	0.98		UGC	21	0.97
	UCA	42	1.36	TER	UGA	37	1.15
	UCG	13	0.52	Trp(W)	UGG	23	1
Pro(P)	CCU	23	1.17	Arg(R)	CGU	8	0.5
	CCC	15	0.7		CGC	7	0.45
	CCA	25	1.46		CGA	12	0.73
	CCG	10	0.66		CGG	9	0.64
Thr(T)	ACU	27	1.2	Ser(S)	AGU	25	0.76
	ACC	19	1.02		AGC	22	0.93
	ACA	30	1.28	Arg(R)	AGA	37	2.37
	ACG	9	0.5		AGG	21	1.31
Ala(A)	GCU	22	1.36	Gly(G)	GGU	18	0.94
	GCC	14	0.87		GGC	13	0.82
	GCA	22	1.28		GGA	25	1.38
	GCG	7	0.49		GGG	15	0.83

**Figure 7:**
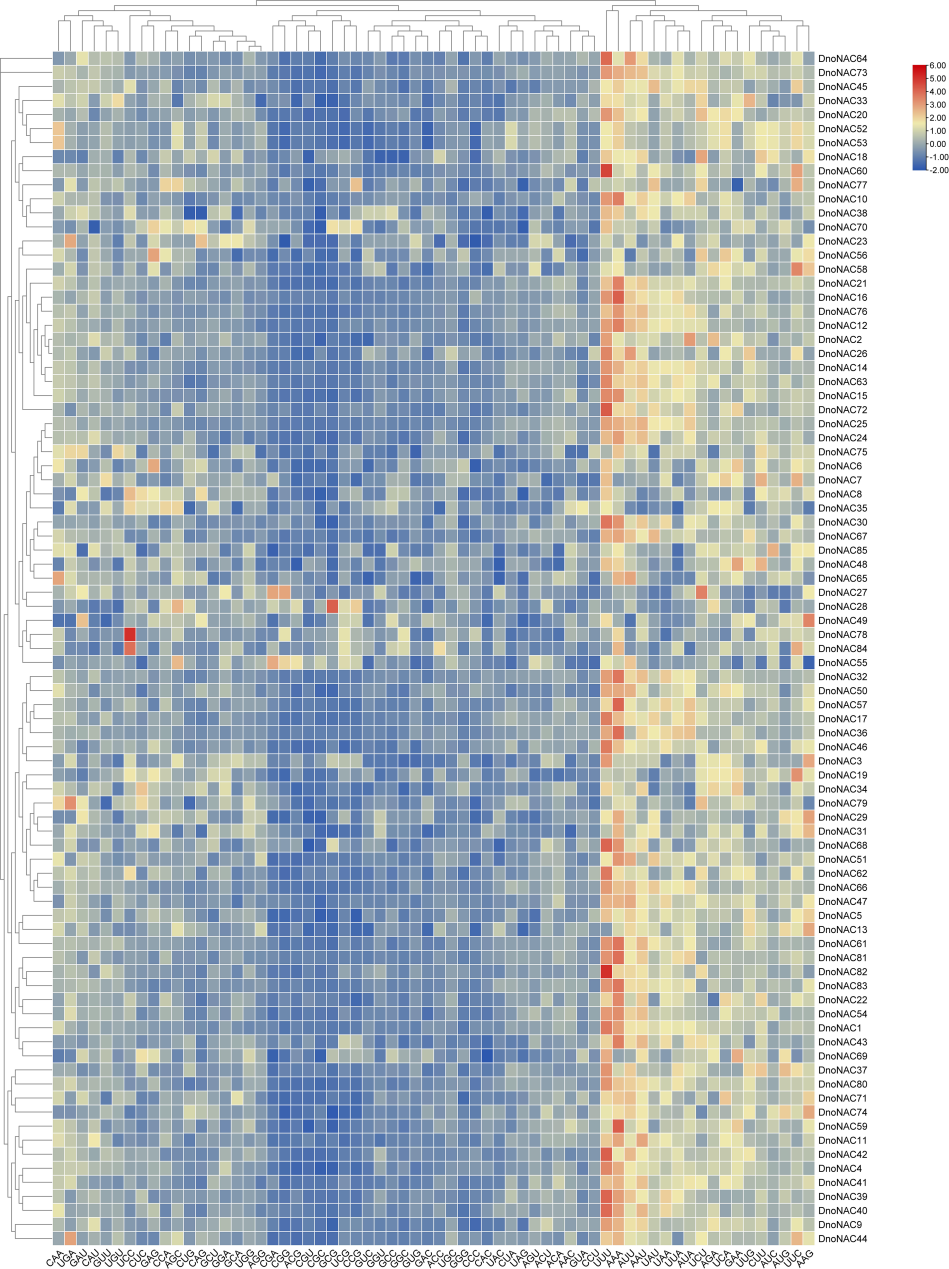
Heat map clustering of codons RSCU of *NAC* gene family in *Dendrobium nobile*.

The heat map clustering of codons RSCU of *NAC* gene family in *D. nobile* revealed that *DnoNAC67* and *DnoNAC85* were clustered separately, *DnoNAC30* and *DnoNAC35* were clustered together, and the remaining genes were clustered together. Members of the same clade show similar codon usage biases, and codons in the same clade have essentially identical RSCU value sizes among different members (**Figure 7**).

### Collinearity analysis

**Figure 8:**
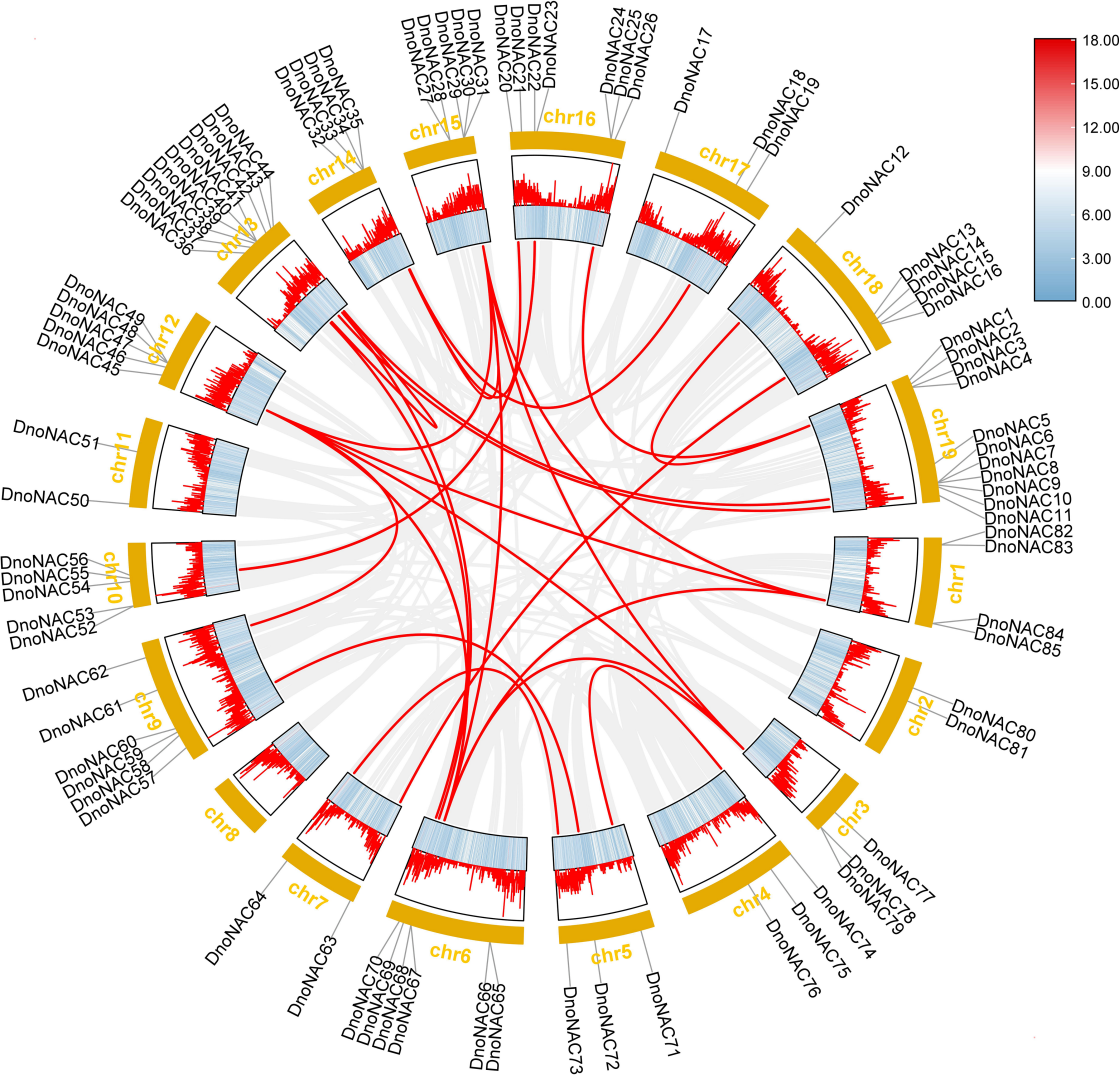
Collinearity analysis of *NAC* gene family in *Dendrobium nobile*.

Gene duplication can occur in a variety of ways, and gene families have been amplified primarily through segmental duplication, tandem duplication, and whole genome duplication(WGD) during organism evolution, with duplicated genes providing control over the physiological and morphological evolution of plants (Kong et al., 2010; Landis et al., 2018). The link between the gene family members was investigated further by comparing the *D. nobile* NAC protein sequences, which revealed 30 pairs of fragment copy genes among the 85 *D. nobile NAC* gene family members(connected by red lines in **Figure 8**). Among the 19 chromosomes, chr6 has the most fragment replication genes, with 8 pairs. Chr4, chr10, and chr17 each has one pair of fragment replication genes. There are no *DnoNAC* fragment replication genes in chr2, chr8, or chr11, pairs of genes within chromosomes are only present in chr13. *D. nobile NAC* family genes are hypothesized to have undergone some scale of fragment duplication events during evolutionary development (**Figure 8**).

Ka/Ks is the ratio of the non-synonymous substitution rate (Ka) to the synonymous substitution rate (Ks) of two protein-coding genes, and it may be used to evaluate if this protein-coding gene is under selection pressure. The Ka/Ks ratios were all less than 1, so the genes were subject to purifying selection in evolution (**Table 4**).

**Table 4:** Gene duplication types and Ka/Ks analysis for duplicated gene pairs of *DnoNAC*s.

Gene name	Gene name	Ka	Ks	Ka/Ks
DnoNAC84	DnoNAC67	0.471603264	3.191892929	0.14775034
DnoNAC84	DnoNAC48	0.481527039	3.300327897	0.145902787
DnoNAC84	DnoNAC30	0.488155543	NaN	NaN
DnoNAC78	DnoNAC67	0.390813882	1.567234946	0.249365217
DnoNAC79	DnoNAC68	0.262750454	4.583184647	0.057329232
DnoNAC78	DnoNAC48	0.50286036	2.698234738	0.186366424
DnoNAC79	DnoNAC47	0.415229286	NaN	NaN
DnoNAC78	DnoNAC30	0.507567853	NaN	NaN
DnoNAC79	DnoNAC31	0.241640178	1.383582099	0.174648239
DnoNAC74	DnoNAC71	0.248151556	0.695755135	0.356665074
DnoNAC73	DnoNAC64	0.455015162	1.078005442	0.422089857
DnoNAC72	DnoNAC58	0.431598801	2.649524768	0.162896685
DnoNAC67	DnoNAC48	0.361952933	1.971219062	0.183618828
DnoNAC68	DnoNAC47	0.410223503	NaN	NaN
DnoNAC69	DnoNAC43	0.248633256	2.172296104	0.114456429
DnoNAC70	DnoNAC38	0.157395091	1.186782439	0.132623374
DnoNAC67	DnoNAC30	0.228928672	1.32738702	0.172465655
DnoNAC63	DnoNAC14	0.151531982	1.025701671	0.147734947
DnoNAC62	DnoNAC47	0.31317265	1.45005749	0.215972575
DnoNAC54	DnoNAC22	0.182243913	0.795892331	0.228980612
DnoNAC47	DnoNAC31	0.408090079	2.049639136	0.19910338
DnoNAC48	DnoNAC30	0.345025465	1.923309873	0.179391511
DnoNAC39	DnoNAC44	0.406261565	NaN	NaN
DnoNAC44	DnoNAC9	0.198890695	0.701997094	0.283321252
DnoNAC42	DnoNAC11	0.27691072	0.893295429	0.309987839
DnoNAC34	DnoNAC31	0.383161758	NaN	NaN
DnoNAC33	DnoNAC20	0.191876401	1.183542234	0.162120451
DnoNAC34	DnoNAC19	0.231490586	1.308010332	0.176979173
DnoNAC26	DnoNAC2	0.146418386	1.009563693	0.145031351
DnoNAC12	DnoNAC2	0.113398959	0.842723598	0.134562458

**Figure 9:**
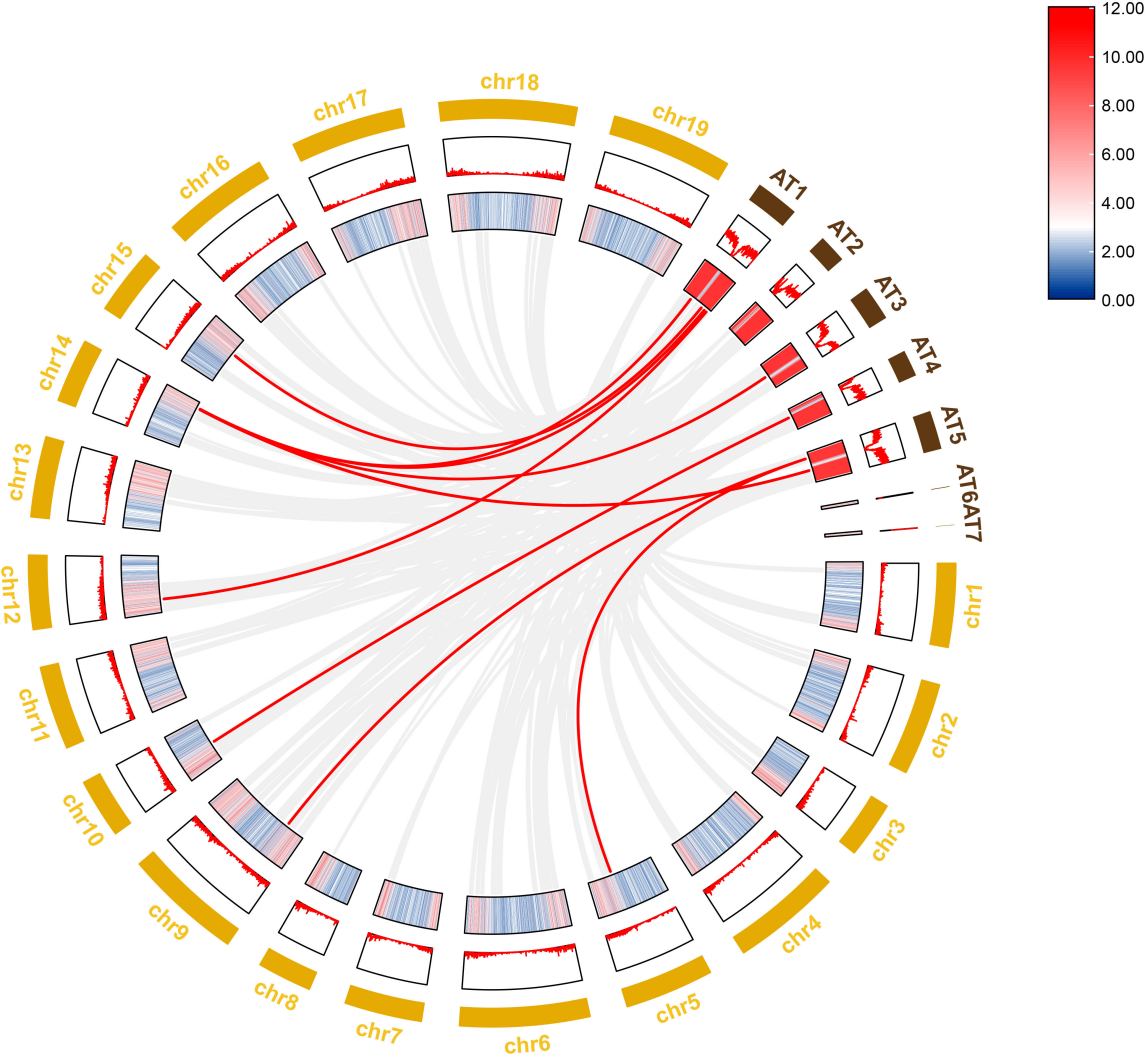
Collinearity analysis of *NAC* gene family between *Dendrobium nobile* (chr1–chr19) and *Arabidopsis thaliana* (AT1–AT7).

To further investigate the evolutionary history of *NAC* genes in various species, collinearity analysis of *D. nobile* and *Arabidopsis NAC* gene family was performed. The results revealing that 8 members of the *D. nobile NAC* gene family have a source relationship with *Arabidopsis* (*DnoNAC27*, *DnoNAC33*, *DnoNAC34*, *DnoNAC35*, *DnoNAC46*, *DnoNAC54*, *DnoNAC58*, *DnoNAC72*), and the most homologous gene pairs exist in chr14(connected by red lines in **Figure 9**). It is speculated that these gene pairs came from the same ancestor.

**Figure 10:**
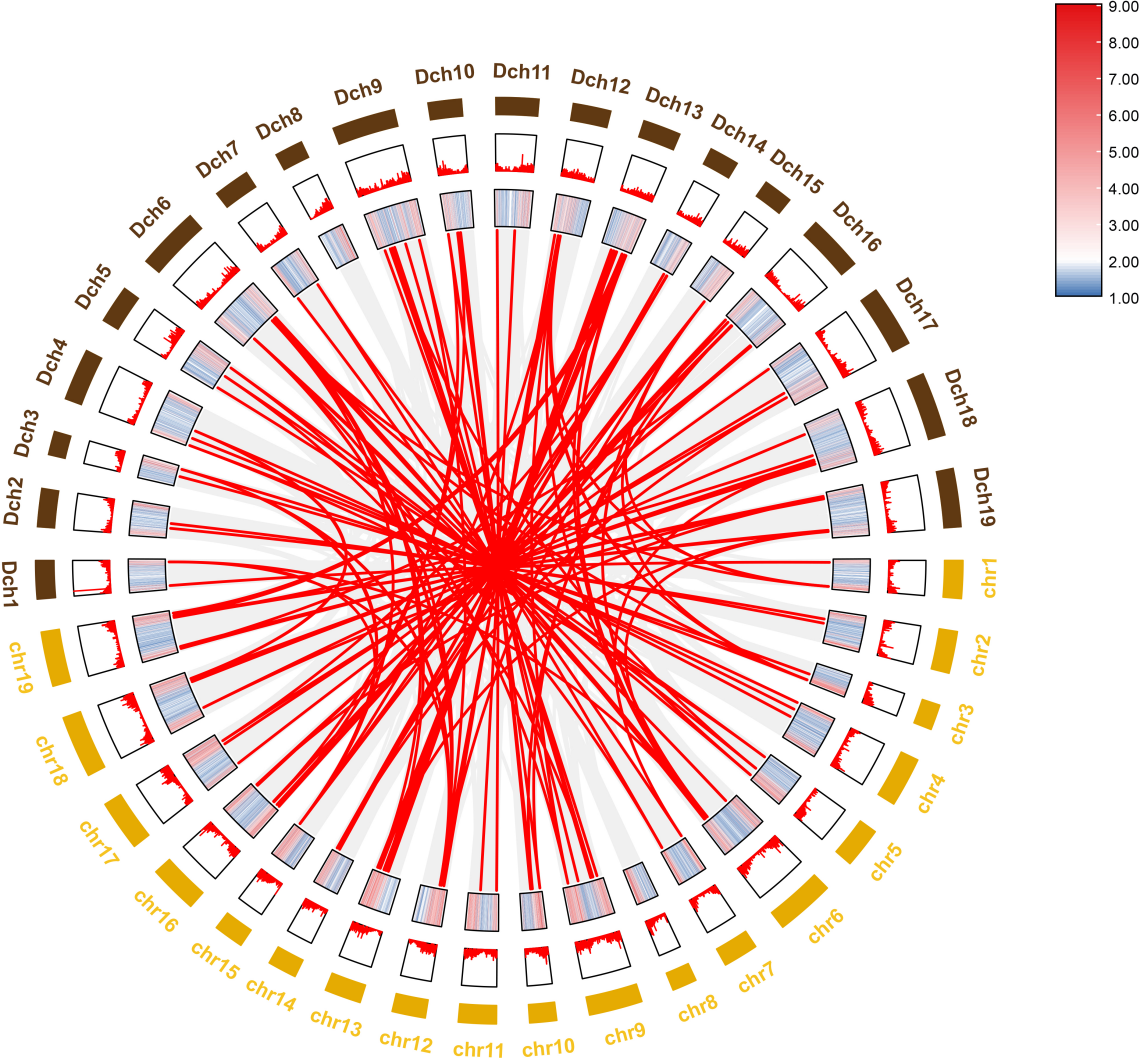
Collinearity analysis of *NAC* gene family between *Dendrobium nobile* (chr1–chr19) and *Dendrobium chrysotoxum* (Dch1–Dch19).

**Figure 11:**
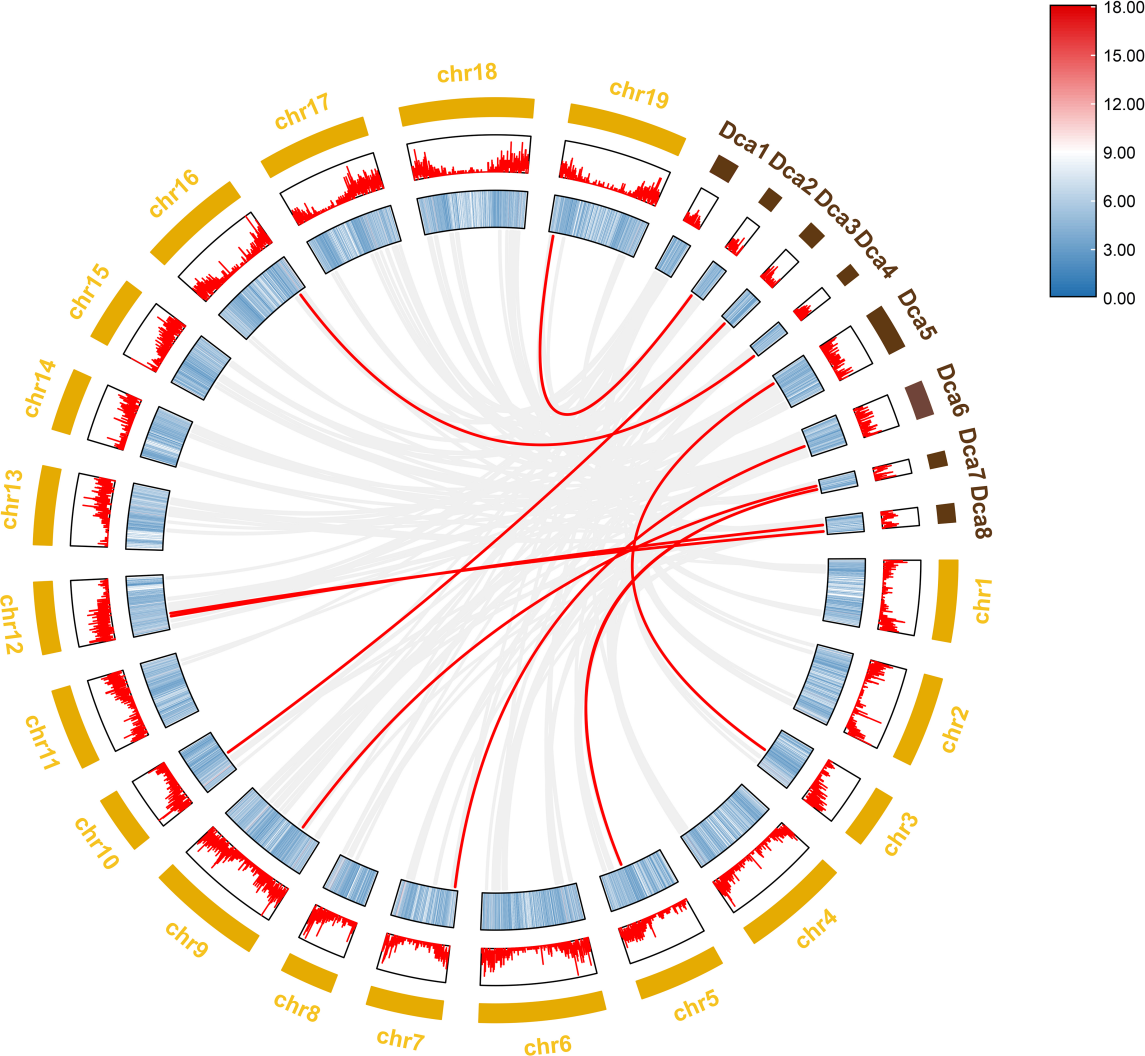
Collinearity analysis of *NAC *gene family between *Dendrobium nobile* (chr1–chr19) and *Dendrobium catenatum* (Dca1–Dca8).

To further investigate the evolutionary history of *NAC* genes in various species, collinearity analysis of *D. nobile* and *Dendrobium chrysotoxum NAC* gene family was performed. The results revealing that 75 members of the *D. nobile NAC* gene family have a source relationship with *Dendrobium chrysotoxum*. There are no homologous gene pair in chr8, two homologous gene pairs in chr2, chr7, and chr11, three homologous gene pairs in chr1, chr3, chr4, chr5, chr15 and chr17, four homologous gene pairs in chr10, chr14 and chr18, five homologous gene pairs in chr12, six homologous gene pairs in chr6, chr9 and chr16, eight homologous gene pairs in chr13 and chr19. There are no *DnoNAC* fragment replication genes in chr8, pairs of genes within chromosomes are only present in chr13(connected by red lines in **Figure 10**). Collinearity analysis of *D. nobile* and *Dendrobium catenatum NAC* gene family reveals that 9 members of the *D. nobile NAC* gene family have a source relationship with *Dendrobium catenatum*. There is one homologous gene pair in chr3, chr5, chr7, chr9, chr10, chr16 and chr19, and two homologous gene pairs in chr12(connected by red lines in **Figure 11**). It is speculated that these gene pairs came from the same ancestor.

**Figure 12:**
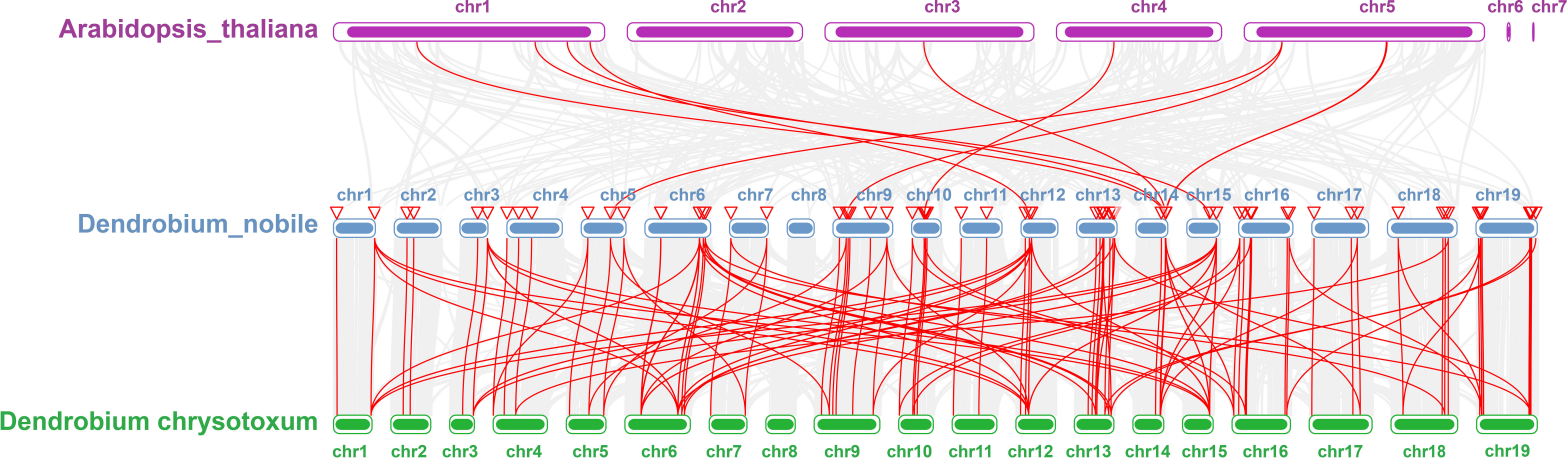
Collinearity analysis of *NAC* gene family between *Dendrobium nobile* (chr1–chr19), *Arabidopsis thaliana* (AT1–AT7) and *Dendrobium chrysotoxum* (Dch1–Dch19).

**Figure 13:**
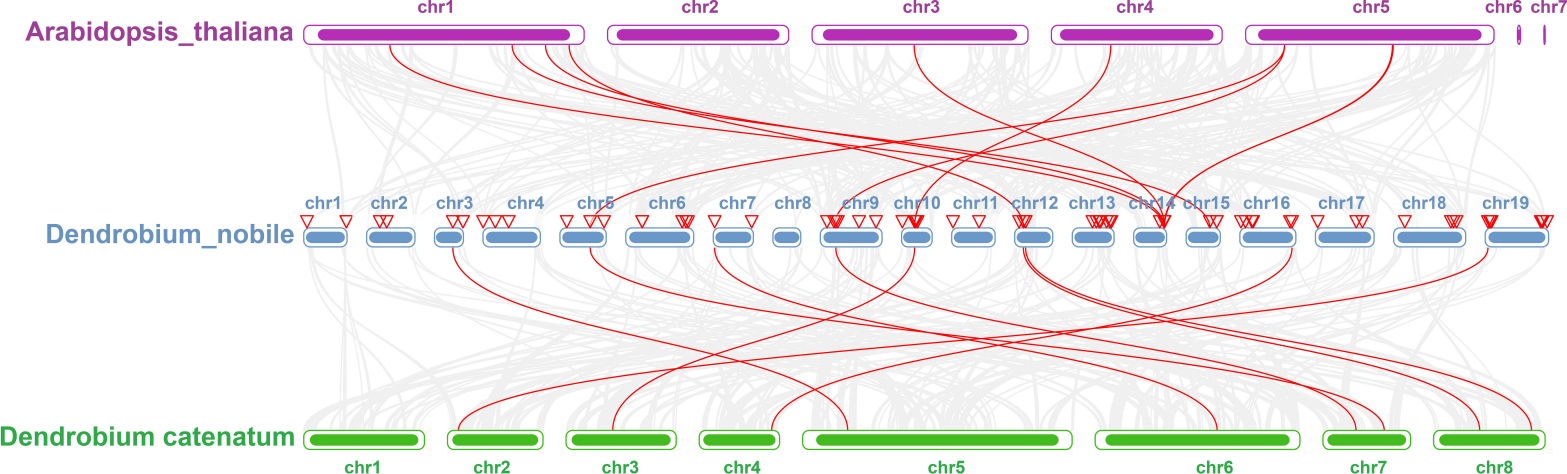
Collinearity analysis of *NAC* gene family between *Dendrobium nobile* (chr1–chr19), *Arabidopsis thaliana* (chr1–chr7) and *Dendrobium catenatum* (chr1–chr8).

Collinearity analysis was performed on *D. nobile*, *Arabidopsis thaliana* and *Dendrobium chrysotoxum* to explore the conservation and variability of *NAC* gene family members over species evolution. During the evolution of *NAC* family gene members in *D. nobile*, the conservation between *D. nobile* and *Dendrobium chrysotoxum* was greater, while the variability between *D. nobile* and *Arabidopsis thaliana* was greater. The collinear loci of *NAC* gene were not evenly distributed on the chromosomes. 9 collinear loci were found on the chromosomes of *D. nobile* with *Arabidopsis thaliana*, and 75 collinear loci with *Dendrobium chrysotoxum* (**Figure 12**). Collinearity analysis of *D. nobile*, *Arabidopsis thaliana*, and *Dendrobium catenatum* revealed that there were a few direct homologous gene pairings and high diversity within the three families (**Figure 13**).

### Prediction of miRNAs targeting in *DnoNAC* genes

397 miRNAs were predicted to target 85 *DnoNAC* genes. The number of target genes of these miRNAs varied greatly, with ath-miR5021 having up to 40 target genes, ath-miR5658 having up to 39 target genes, ath-miR5640 having up to 33 target genes, but 65 miRNAs having only one (ath-miR158a-3p, ath-miR158b, ath-miR161.1, ath-miR162a-3p etc.). The majority of miRNA mature sequences (5’ – 3’) were 21 bp in length, accounting for 76.20% of all sequences. The miRNA mature sequences (5’ – 3’) were 19 bp and 26 bp in length each accounting for 0.03% of all sequences (**Supplementary Table 5**).

### Analysis of SSR loci in *DnoNAC* genes

The study’s findings revealed that *D. nobile* SSR locis were rich in repeat types such as mononucleotide, dinucleotide, trinucleotide, hexanucleotide, and compound nucleotide. The number of each repeat type varied substantially, but mononucleotide repeats dominated, accounting for 58.91% of all SSRs with a total length of 1324 bp. The distribution of mononucleotide ssr loci displayed a clear preference, with the ssr loci number of motif A/T is 102, while the motif C/G is just 7. Dinucleotide repeats accounting for 14.59% of all SSRs with a total length of 820 bp and the dominant motif type is AT/TA. Trinucleotide repeats accounting for 11.89% of all SSRs with a total length of 417 bp. Hexanucleotide repeats accounting for 0.05% of all SSRs with a total length of 30 bp. Compound repeats accounting for 13.51% of all SSRs with a total length of 2050 bp. Overall, The majority of SSR loci in *D. nobile* were less than 50 bp long, accounting for 85.94% of all SSRs (**Supplementary Table 6**).

### Expression of *DnoNAC* genes under salt stress and different temperature conditions

**Figure 14:**
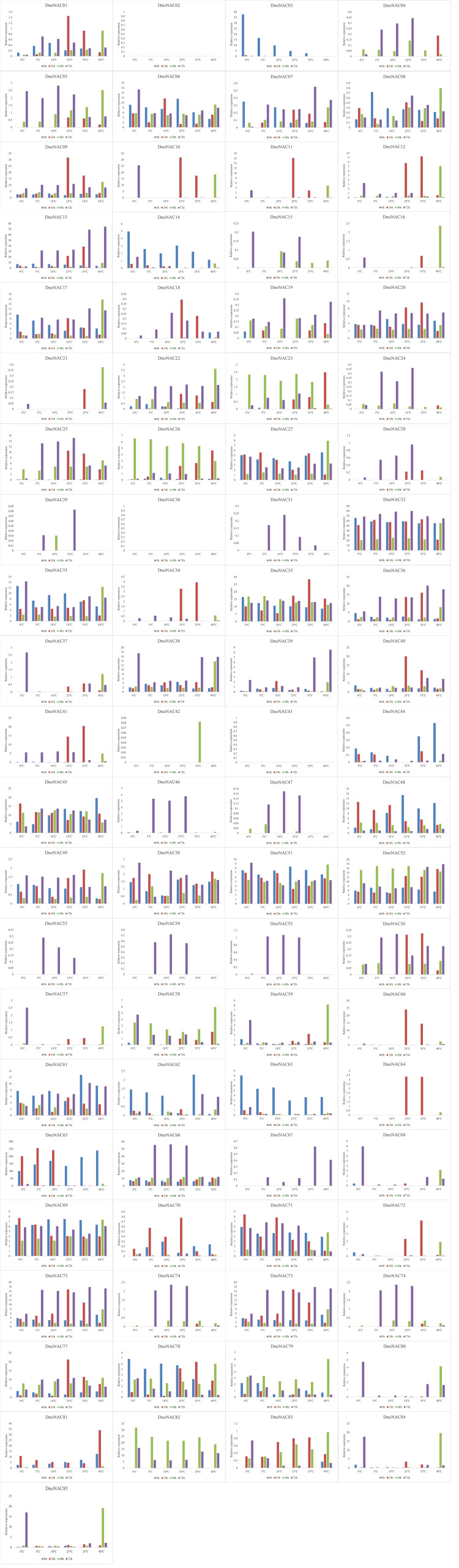
Expression level of *DnoNAC* genes at 0h, 24h, 48h and 72h under diffenrent temperature stress.

The gene expression of 85 *DnoNAC* genes in leaves of *D. nobile* seedlings under different temperature stress treatments was analyzed by qRT-PCR, and the results showed that changes in gene expression level were rather small after 48 hours of the low temperature stress treatment. After 72 hours of low temperature treatment, the expression level of *DnoNAC3*, *DnoNAC7*, *DnoNAC14*, *DnoNAC62*, *DnoNAC63*, *DnoNAC65*, *DnoNAC76* and *DnoNAC78* genes showed a decreasing trend, and a total of 20 *DnoNAC* genes with *DnoNAC5*, *DnoNAC9*, *DnoNAC10*, *DnoNAC15*, *DnoNAC19*, *DnoNAC24*, *DnoNAC28*, *DnoNAC31*, *DnoNAC37*, *DnoNAC46*, *DnoNAC53*, *DnoNAC54*, *DnoNAC55*, *DnoNAC57*, *DnoNAC58*, *DnoNAC59*, *DnoNAC68*, *DnoNAC79*, *DnoNAC84* and *DnoNAC85* showed an increasing trend in expression, in which the expression of *DnoNAC37*, *DnoNAC57* and *DnoNAC68* was significantly increased. The expression level of *DnoNAC9*, *DnoNAC10*, *DnoNAC11*, *DnoNAC18*, *DnoNAC34*, *DnoNAC40*, *DnoNAC56*, *DnoNAC60*, *DnoNAC64* and *DnoNAC72* genes increased significantly after 24 hours of high-temperature stress treatment. After 48 hours of high temperature stress treatment, the gene expression of *DnoNAC16*, *DnoNAC21*, *DnoNAC42*, *DnoNAC59*, and *DnoNAC80* rose considerably. the gene expression of *DnoNAC7*, *DnoNAC13*, *DnoNAC32*, *DnoNAC33*, *DnoNAC36*, *DnoNAC38*, *DnoNAC39* and *DnoNAC67* showed an increasing trend after 72 hours of high-temperature stress treatment (**Figure 14**). In short, the *NAC* transcription factors may play an important part in cold and high temperature resistance in *D. nobile* and must be highly expressed following a period of stress.

**Figure 15:**
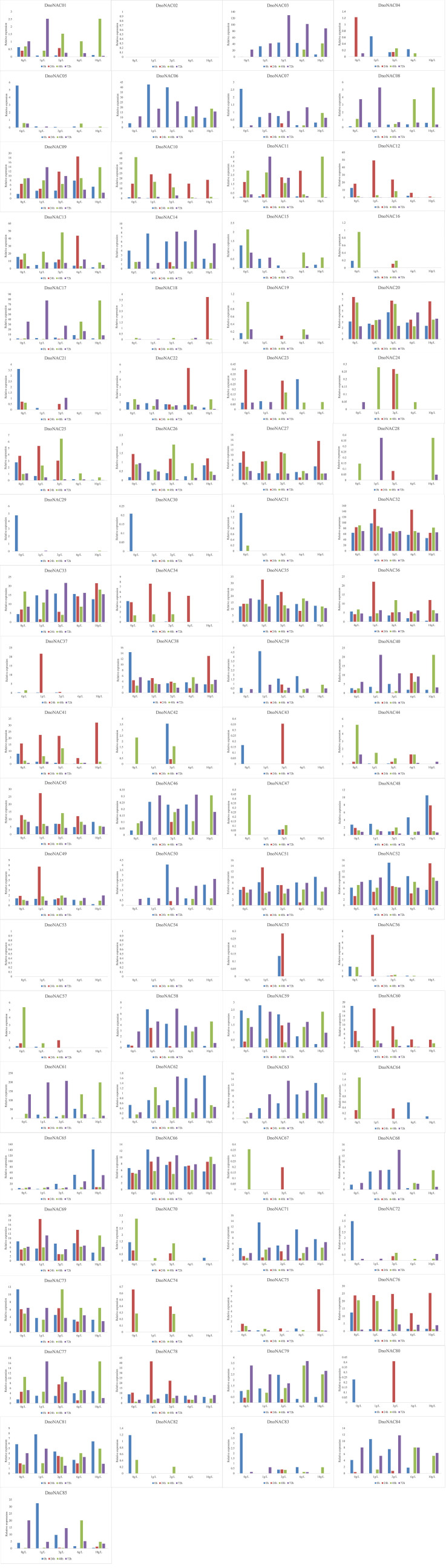
Expression level of *DnoNAC* genes at 0h, 24h, 48h and 72h under salt stress.

The gene expression of 85 *DnoNAC* genes in leaves of *D. nobile* seedlings under different salt concentration stress treatments was analyzed by qRT-PCR. After 24 hours of salt stress treatment, the gene expression of *DnoNAC25*, *DnoNAC34*, *DnoNAC35*, *DnoNAC36*, *DnoNAC37*, *DnoNAC43*, *DnoNAC45*, *DnoNAC49*, *DnoNAC51*, *DnoNAC55*, *DnoNAC56*, *DnoNAC60*, *DnoNAC78* and *DnoNAC80* had a tendency to decline with an increase in salt concentration. The gene expression of *DnoNAC10*, *DnoNAC15*, *DnoNAC16*, *DnoNAC19*, *DnoNAC44*, *DnoNAC47*, *DnoNAC57*, *DnoNAC64* and *DnoNAC70* showed a declining trend under low salt concentration stress after 48 hours of salt stress treatment. After 72 hours of salt stress treatment, there was an increasing trend in the expression of *DnoNAC3*, *DnoNAC14* and *DnoNAC50*(**Figure 15**). The findings imply that salt stress regulates the expression of *D. nobile NAC* family genes, which may be crucial in response to salt stress.

The above results indicate that most members of the *DnoNAC* gene family in leaves of *D. nobile* seedlings have sensitive response mechanisms to salt stress, low temperature and high temperature stress, indicating that most members of the *DnoNAC* genes have molecular functions to regulate salt stress, low temperature and high temperature stress during the growth and development of *D. nobile.*

## Discussion

Gene expression regulation is a way of regulating plant stress in response to adversity and plays a crucial role in plant growth and development, with the fundamental regulatory elements being transcription factors. Plant transcription factors have long been a focus of functional genomics research. As a trans-acting factor, it interacts with cis-elements of particular target genes to regulate various signaling pathways (Kumar et al., 2021). To date, thousands of transcription factors have been found in higher plants, with a considerable fraction being linked to resistance to or eradication of drought, high salinity, low and high temperatures (Liu and Zou, 2010) The *NAC* gene family is a group of transcription factors found in green plants. According to reports, there are 117 *NAC* genes in *Arabidopsis* (Ooka, 2003; Nuruzzaman et al., 2010), 151 in *Rice* (Yuan et al., 2019), 110 in *Potato* (Singh et al., 2013), 152 in *Tobacco* (Rushton et al., 2008), and 152 in *Soybean* (Le et al., 2011). In the present study, 85 *NAC* genes were identified in *D. nobile*, the number of which was lower than that of arabidopsis, rice and soybean. This discrepancy in number might be attributed to the fact that *D. nobile*’s genome developed without polyploidization events, which is compatible with the findings of academic investigations on the tomato *NAC* gene family (Jin et al., 2020).

The physicochemical properties, subcellular localization, conserved motifs, protein structure, codon bias, collinearity, phylogenetic tree construction and cis-elements of 85 *D. nobile NAC* transcription factor were analyzed in this study using relevant databases and software tools. The interrelationships among different *D. nobile NAC* gene family members were obtained.

The bioinformatics analysis of *D. nobile NAC* gene family members revealed that the isoelectric points of *D. nobile NAC* gene family members span a wide range, indicating that the encoded proteins can adapt to different acidic and alkaline environments. The total average hydrophilicity was negative, indicating that *D. nobile* NAC proteins are relatively hydrophilic proteins. The *D. nobile NAC* gene family contains 54 negatively charged residues, 8 electrically neutral residues and 23 positively charged residues, presumably the *D. nobile* NAC protein is negatively charged.

The highest proportion of secondary structures in this gene family is random coiled, followed by the alpha-helix and extended strand, and the least proportion of beta-turn. The overall proportions are relatively consistent and it can be assumed that *D. nobile NAC* family are relatively conservative in structure. The spatial conformation of the protein folding in the tertiary structure is highly conserved, but the tertiary structures of *DnoNAC14*, *DnoNAC21*, and *DnoNAC27* are more distinct from the other family members, as are the corresponding conserved motifs. The tertiary structure of *DnoNAC27* is the simplest, lacking a large number of conserved motifs and comprising just motif2 and motif4. The roles of *DnoNAC14*, *DnoNAC21*, and *DnoNAC27* are thought to be slightly different from those of other family members. This is consistent with the findings of scientific investigations on the sesame *NAC* transcription factor family (Zhang et al., 2018).

According to the projected subcellular localization data, the places where *D. nobile NAC* transcription factors work are somewhat distributed, but the majority of members are located in the nucleus. This implies that *D. nobile NAC* transcription factors may behave differently in distinct subcellular locations, with the nucleus serving as the primary site of action. The precise location of each family member requires futuer investigation. This is consistent with the findings of scientific investigations on the *Passiflora edulis NAC* transcription factor family (Yang et al., 2022).

The analysis of codon use bias revealed that 85 family members exhibited slight preference for codon selection. Only the RSCU of AGA is greater than 2, indicating that AGA is strongly preferred by family members. The average GC3s and GC content was less than 50%, and the high-use codon preference ended in A/U(T), suggesting that A/U(T) was utilized more frequently than G/C in the coding sequence codon.

From the phylogenetic tree of *NAC* gene family in *D. nobile*, it can be seen that the *D. nobile NAC* gene family can be divided into 11 subfamilies and the family members are unevenly distributed in each subfamily. Combining the results of collinearity analysis and the phylogenetic tree of *NAC* gene family in *D. nobile* and *Arabidopsis NAC* gene family, it was discovered that there are eight pairings of genes with a high degree of similarity(*DnoNAC27* and *AT1G61110.1*, *DnoNAC33* and *AT1G26870.1*, *DnoNAC34* and *AT3G29035.1*, *DnoNAC35* and *AT1G69490.1*, *DnoNAC46* and *AT1G76420.1*, *DnoNAC54* and *AT4G10350.1*, *DnoNAC54* and *AT4G10350.1 DnoNAC58* and *AT5G13180.1*, *DnoNAC72* and *AT5G13180.1*). Except for *DnoNAC46* and *AT1G76420.1*, which are found in neighboring subclades, the remaining seven pairs of genes are found on the same subclade and are quite near together, presumably their biological functions are also similar. Based on the phylogenetic tree of *D. nobile* and other typical species, it is known that the *D. nobile NAC* gene family is most closely related to *Dendrobium catenatum* and *Dendrobium chrysotoxum*.

The cis-acting element of the promoter region regulates the exact commencement of gene transcription and transcriptional efficiency by binding to transcription factors, it can determine the core area of transcriptional activation (Wittkopp and Kalay, 2011; Ding et al., 2021; Liu et al., 2023; Rui et al., 2023). The *NAC* family has a substantial amount of cis-acting elements associated to light sensitivity, hormone response, biotic and abiotic stress response that are speculated to have a role in *D. nobile* growth and development, stress tolerance, and hormone signaling. This is consistent with the findings of Saidi et al. on the *NAC* gene family in other plants (Ling et al., 2017; Saidi et al., 2017; Liu et al., 2018; Wang et al., 2023).

MiRNAs regulate many genes and so act in multiple biological processes, demonstrating the complexity of miRNA control of target genes. The study identified ath-miR5021 with a high number of target genes and the majority of miRNA mature sequences(5’ – 3’) were 21 bp in length. Analysis of SSR loci through MISA-web software indicates that the fraction of mononucleotide repeats was the largest, as was the frequency of A/T. AT/GC is the prominent motif for dinucleotides. Excluding compound nucleotides, The SSR motif type grow with the number of motif, while the number of SSR loci decreases with the number of motif.

In this study, 85 *DnoNAC* genes in *D. nobile* genome were identified, with its amino acid length ranged from 80 to 1065. Its promoter region contains multiple stress responsive elements, including light responsive, gibberellin-responsive, abscisic acid responsiveness, MeJA-responsiveness and drought-inducibility elements. *NAC* gene family in *D. nobile* is most closely related to that of *Dendrobium catenatum* and *Dendrobium chrysotoxum*. The fraction of mononucleotide repeats in its SSRs was the largest, as was the frequency of A/T. These 85 *DnoNAC* genes contain 397 miRNAs. The collinearity analysis shows that 9 collinear locis were found on the chromosomes of *D. nobile* with *Arabidopsis thaliana*, and 75 collinear locis with *D.chrysotoxum.* The response mechanism of *DnoNAC* gene family in leaves of *D. nobile* seedlings to salt stress, low temperature and high temperature stress was verified by qRT-PCR experiment. These results provide a reference for further understanding the function of *NAC* gene family in *D. nobile*.

## Data availability statement

The datasets presented in this study can be found in online repositories. The names of the repository/repositories and accession number(s) can be found in the article/**Supplementary Material**.

## Author contributions

CF: Experimental design, Resources, Funding acquisition, Writing-original draft,Writing-review & editing. MYL: Investigation, Experimental operations, Formal analysis, Visualization, Writing-original draft. All authors contributed to the article and approved the submitted version.
