# Supplementary material for: Genome-wide identification and molecular evolution of NAC gene family in Dendrobium nobile
Source: Front Plant Sci. 2023 Aug 21;14:1232804. doi: 10.3389/fpls.2023.1232804 (PMC10475575; doi:10.3389/fpls.2023.1232804)
Supplement: Supplementary file 1 [file DataSheet_1.zip › Supplementary Table Legends.docx]

**Supplementary Table Legend**

Supplementary Table1 qRT-PCR primer list for *DnoNAC* genes

Supplementary Table2. Hydrophilicity/hydrophobicity analysis of NAC proteins in *Dendrobium nobile*

Supplementary Table 3. Secondary structure analysis of NAC proteins from *Dendrobium nobile*

Supplementary Table 4. Base compositions and related codon parameters of *NAC* family members in *Dendrobium nobile*

Supplementary Table 5 Predicted miRNAs targeting *DnoNAC* genes

Supplementary Table 6 Predicted SSR loci in *DnoNAC* genes/promoters
