# Supplementary material for: Genome-wide identification and molecular evolution of NAC gene family in Dendrobium nobile
Source: Front Plant Sci. 2023 Aug 21;14:1232804. doi: 10.3389/fpls.2023.1232804 (PMC10475575; doi:10.3389/fpls.2023.1232804)
Supplement: Supplementary file 1 [file DataSheet_1.zip › Supplementary Tables 1-4.docx]

Supplementary Table1 qRT-PCR primer list for *DnoNAC* genes

| Sequence ID | Primer ID | Primer Sequence | Primer Tm | Primer GC | Product Size |
| --- | --- | --- | --- | --- | --- |
| DnoNAC1 | DnoNAC1_1_F | AAAGCTCCACCATGCCCC | 59.962 | 61.111 | 144 |
| DnoNAC1 | DnoNAC1_1_R | GGCAGCCGAGGACTGATC | 59.892 | 66.667 | 144 |
| DnoNAC10 | DnoNAC10_1_F | CGGGCAGCCATGGAAGAA | 60.045 | 61.111 | 150 |
| DnoNAC10 | DnoNAC10_1_R | CTCGGCCGCCTCTATTGG | 59.97 | 66.667 | 150 |
| DnoNAC11 | DnoNAC11_1_F | GCCGGTAACCTTCCGCTT | 60.047 | 61.111 | 82 |
| DnoNAC11 | DnoNAC11_1_R | GACGCAACTAGCCCCTGG | 60.125 | 66.667 | 82 |
| DnoNAC12 | DnoNAC12_1_F | CCGTTTTGTGCCACCGTG | 59.975 | 61.111 | 111 |
| DnoNAC12 | DnoNAC12_1_R | AGCACTTCTTCCACCGGC | 59.967 | 61.111 | 111 |
| DnoNAC13 | DnoNAC13_1_F | GGCTTTCGCTTCCACCCT | 59.966 | 61.111 | 131 |
| DnoNAC13 | DnoNAC13_1_R | GGAAGGTCCCATGGCTCAC | 60.077 | 63.158 | 131 |
| DnoNAC14 | DnoNAC14_1_F | TAAGGTGCTTTGGCcccc | 59.557 | 61.111 | 84 |
| DnoNAC14 | DnoNAC14_1_R | ACAGCACCGCATCTCTCG | 59.817 | 61.111 | 84 |
| DnoNAC15 | DnoNAC15_1_F | GATCCTCCAGCCGATGCC | 59.968 | 66.667 | 148 |
| DnoNAC15 | DnoNAC15_1_R | tgcttcgcactgagctcc | 60.048 | 61.111 | 148 |
| DnoNAC16 | DnoNAC16_1_F | gttccgaGGACACTGCCC | 60.047 | 66.667 | 92 |
| DnoNAC16 | DnoNAC16_1_R | GGCTCCTTgacggggttc | 60.046 | 66.667 | 92 |
| DnoNAC17 | DnoNAC17_1_F | TGGGAGTCTCGAGCCGAA | 59.965 | 61.111 | 150 |
| DnoNAC17 | DnoNAC17_1_R | GGAAGGCAACGGGGAAGG | 60.361 | 66.667 | 150 |
| DnoNAC18 | DnoNAC18_1_F | ACTGGCAGCGATAGGGGA | 60.042 | 61.111 | 115 |
| DnoNAC18 | DnoNAC18_1_R | TCCGGTCGCCTTCCAGTA | 59.964 | 61.111 | 115 |
| DnoNAC19 | DnoNAC19_1_F | GAGAGCGGTGGACATGGG | 59.812 | 66.667 | 101 |
| DnoNAC19 | DnoNAC19_1_R | GTCACGTGCCCTGCTTCT | 59.969 | 61.111 | 101 |
| DnoNAC2 | DnoNAC2_1_F | GGTGACAGCGAACGGGAA | 59.97 | 61.111 | 81 |
| DnoNAC2 | DnoNAC2_1_R | TCCGGCTTCCTGTGCTTG | 59.967 | 61.111 | 81 |
| DnoNAC20 | DnoNAC20_1_F | AGGCCAACACCTCAGCAC | 59.886 | 61.111 | 96 |
| DnoNAC20 | DnoNAC20_1_R | TGGCACGGGGAAGAGAGA | 59.88 | 61.111 | 96 |
| DnoNAC21 | DnoNAC21_1_F | TGGTTGGGCGGGCTAAAG | 59.965 | 61.111 | 112 |
| DnoNAC21 | DnoNAC21_1_R | GTGGCTGCTCGGACTGTT | 59.969 | 61.111 | 112 |
| DnoNAC22 | DnoNAC22_1_F | GCGCGTTTCTTCGGCAAG | 60.5 | 61.111 | 127 |
| DnoNAC22 | DnoNAC22_1_R | GAGCTCCAGCCAAACGGT | 59.967 | 61.111 | 127 |
| DnoNAC23 | DnoNAC23_1_F | AAAGGGCAGGCCAAAGCA | 60.123 | 55.556 | 142 |
| DnoNAC23 | DnoNAC23_1_R | CCTGACTCGCACACTCGG | 60.126 | 66.667 | 142 |
| DnoNAC24 | DnoNAC24_1_F | GCGCTGCACACAAAGCTT | 59.973 | 55.556 | 130 |
| DnoNAC24 | DnoNAC24_1_R | AGAGGGAGGTCCGGCTTT | 59.877 | 61.111 | 130 |
| DnoNAC25 | DnoNAC25_1_F | CGGGCATCTAGGCTTCGG | 59.97 | 66.667 | 113 |
| DnoNAC25 | DnoNAC25_1_R | CGATGGCATTTTCCGGCG | 59.975 | 61.111 | 113 |
| DnoNAC26 | DnoNAC26_1_F | CAGGCTTCGTCTTCCCCG | 60.126 | 66.667 | 129 |
| DnoNAC26 | DnoNAC26_1_R | AGCACTTCTTCCACCGGC | 59.967 | 61.111 | 129 |
| DnoNAC27 | DnoNAC27_1_F | TCGGCGATGATGGTGACG | 59.896 | 61.111 | 145 |
| DnoNAC27 | DnoNAC27_1_R | AGAGCACCGACTCCTCGT | 59.966 | 61.111 | 145 |
| DnoNAC28 | DnoNAC28_1_F | AGAGCACCGACTCCTCGT | 59.966 | 61.111 | 135 |
| DnoNAC28 | DnoNAC28_1_R | TGGTGACGGGGAGAGGAG | 59.963 | 66.667 | 135 |
| DnoNAC29 | DnoNAC29_1_F | TTCAGTCGTGGCTGCCTG | 59.969 | 61.111 | 98 |
| DnoNAC29 | DnoNAC29_1_R | CCGGTCGGTGCTGATACC | 59.894 | 66.667 | 98 |
| DnoNAC3 | DnoNAC3_1_F | ACATGCTCCAGCGTCACC | 60.047 | 61.111 | 134 |
| DnoNAC3 | DnoNAC3_1_R | GCGGCTGGGAAGTGACTT | 59.967 | 61.111 | 134 |
| DnoNAC30 | DnoNAC30_1_F | AAGGCTCTGTTCGGCGAC | 60.048 | 61.111 | 112 |
| DnoNAC30 | DnoNAC30_1_R | CGGTCGCCTTCCAGTACC | 59.816 | 66.667 | 112 |
| DnoNAC31 | DnoNAC31_1_F | TTCAGTCGTGGCTGCCTG | 59.969 | 61.111 | 98 |
| DnoNAC31 | DnoNAC31_1_R | CCGGTCGGTGCTGATACC | 59.894 | 66.667 | 98 |
| DnoNAC32 | DnoNAC32_1_F | tacccagTGCTTGTGCGG | 59.968 | 61.111 | 139 |
| DnoNAC32 | DnoNAC32_1_R | GTGGATGCTGACCCACCC | 60.045 | 66.667 | 139 |
| DnoNAC33 | DnoNAC33_1_F | CGGCCGGATGCGAATTTG | 59.975 | 61.111 | 97 |
| DnoNAC33 | DnoNAC33_1_R | GCCGCGGCTGGAAGATAT | 59.969 | 61.111 | 97 |
| DnoNAC34 | DnoNAC34_1_F | TGCCAGAGCGAGTGGGTA | 60.284 | 61.111 | 150 |
| DnoNAC34 | DnoNAC34_1_R | CGGGGGAGTTGGAGTTGG | 59.649 | 66.667 | 150 |
| DnoNAC35 | DnoNAC35_1_F | GAGGGGGCTGTGTGTGTC | 59.967 | 66.667 | 117 |
| DnoNAC35 | DnoNAC35_1_R | CGCTCTGTCGGGGTGAAG | 60.126 | 66.667 | 117 |
| DnoNAC36 | DnoNAC36_1_F | TGGGGTGTTTCAAGGCGA | 59.08 | 55.556 | 147 |
| DnoNAC36 | DnoNAC36_1_R | GGCCACAGGAAAAGACCGA | 59.927 | 57.895 | 147 |
| DnoNAC37 | DnoNAC37_1_F | AGGCGCGTCTTGCTGATT | 60.047 | 55.556 | 137 |
| DnoNAC37 | DnoNAC37_1_R | TTCCGTGGCCCGTTTGAA | 59.808 | 55.556 | 137 |
| DnoNAC38 | DnoNAC38_1_F | TCTTGCTGCCACGTGGAG | 59.969 | 61.111 | 126 |
| DnoNAC38 | DnoNAC38_1_R | AACGCGGGTTCTGGAAGG | 59.968 | 61.111 | 126 |
| DnoNAC39 | DnoNAC39_1_F | AACGCATGGCTCAGTGGT | 59.568 | 55.556 | 80 |
| DnoNAC39 | DnoNAC39_1_R | gccgtcccaaacaaagcc | 59.661 | 61.111 | 80 |
| DnoNAC4 | DnoNAC4_1_F | tcggggaggtggcataca | 59.96 | 61.111 | 93 |
| DnoNAC4 | DnoNAC4_1_R | gctcccccaccactttcc | 59.964 | 66.667 | 93 |
| DnoNAC40 | DnoNAC40_1_F | cacggacacgacacgaca | 59.975 | 61.111 | 82 |
| DnoNAC40 | DnoNAC40_1_R | tattgccgtgtcctcgcc | 59.814 | 61.111 | 82 |
| DnoNAC41 | DnoNAC41_1_F | GGCAGTTCCCAGGGTTCG | 60.36 | 66.667 | 145 |
| DnoNAC41 | DnoNAC41_1_R | TGGCTCTTCCTCCTGGCT | 59.876 | 61.111 | 145 |
| DnoNAC42 | DnoNAC42_1_F | caggtggtggtctggcag | 59.967 | 66.667 | 132 |
| DnoNAC42 | DnoNAC42_1_R | gaaccaccacctgaggcc | 59.966 | 66.667 | 132 |
| DnoNAC43 | DnoNAC43_1_F | AAACGGCTCGTCGAAGGG | 60.049 | 61.111 | 83 |
| DnoNAC43 | DnoNAC43_1_R | GGATTCGGTGACTGGGCC | 60.125 | 66.667 | 83 |
| DnoNAC44 | DnoNAC44_1_F | CCAAGCCCAATGCATTGCA | 59.703 | 52.632 | 114 |
| DnoNAC44 | DnoNAC44_1_R | TGCACTAGCTTGACGGCA | 59.26 | 55.556 | 114 |
| DnoNAC45 | DnoNAC45_1_F | CCACTGTCCAGCTGCACA | 59.888 | 61.111 | 141 |
| DnoNAC45 | DnoNAC45_1_R | TCTGCGGATTCGAGCTCG | 59.591 | 61.111 | 141 |
| DnoNAC46 | DnoNAC46_1_F | AGGAAGTTGGTGGCGAGC | 59.967 | 61.111 | 126 |
| DnoNAC46 | DnoNAC46_1_R | CCGCAATATCAACGCCGC | 60.052 | 61.111 | 126 |
| DnoNAC47 | DnoNAC47_1_F | ATGGCGATCGGGCTGAAG | 59.89 | 61.111 | 82 |
| DnoNAC47 | DnoNAC47_1_R | GATTCCACCCGACCGACG | 60.203 | 66.667 | 82 |
| DnoNAC48 | DnoNAC48_1_F | GATCGACGAAGGGCAGGG | 59.893 | 66.667 | 115 |
| DnoNAC48 | DnoNAC48_1_R | GTCCAACGGCTCACCCTC | 60.047 | 66.667 | 115 |
| DnoNAC49 | DnoNAC49_1_F | ATGGAGGCGGTGGAGGAT | 60.04 | 61.111 | 111 |
| DnoNAC49 | DnoNAC49_1_R | AGACGTCCGCAAACCAGG | 59.969 | 61.111 | 111 |
| DnoNAC5 | DnoNAC5_1_F | CCTTGGGAGCCCTACCCT | 59.957 | 66.667 | 121 |
| DnoNAC5 | DnoNAC5_1_R | AATCGAGCCACACACGCA | 59.97 | 55.556 | 121 |
| DnoNAC50 | DnoNAC50_1_F | AGTTTGCGGGATGGACGG | 60.046 | 61.111 | 121 |
| DnoNAC50 | DnoNAC50_1_R | TTCGTGTCAGGTGTCGGC | 59.972 | 61.111 | 121 |
| DnoNAC51 | DnoNAC51_1_F | AGCCCAGCGCAAGAGTTT | 59.886 | 55.556 | 136 |
| DnoNAC51 | DnoNAC51_1_R | GCTCCAGTTGACCCTCCAC | 60.003 | 63.158 | 136 |
| DnoNAC52 | DnoNAC52_1_F | TGCGACCTCCATGCTTCC | 59.731 | 61.111 | 87 |
| DnoNAC52 | DnoNAC52_1_R | TGCGGCTGCCCAAAGTTT | 60.837 | 55.556 | 87 |
| DnoNAC53 | DnoNAC53_1_F | TGCGACCTCCATGCTTCC | 59.731 | 61.111 | 99 |
| DnoNAC53 | DnoNAC53_1_R | CCACTAGACTGCTGCGGC | 60.51 | 66.667 | 99 |
| DnoNAC54 | DnoNAC54_1_F | CCATTTGGTGCTCGGCCT | 60.362 | 61.111 | 119 |
| DnoNAC54 | DnoNAC54_1_R | AGGGCCACTGCTTCTGGA | 60.53 | 61.111 | 119 |
| DnoNAC55 | DnoNAC55_1_F | ATCGGTACCCCCAAGCCT | 59.958 | 61.111 | 138 |
| DnoNAC55 | DnoNAC55_1_R | ATTTTTCCTGCGGGCGGA | 59.965 | 55.556 | 138 |
| DnoNAC56 | DnoNAC56_1_F | TGCTGGGATGGAAGGGGA | 59.873 | 61.111 | 83 |
| DnoNAC56 | DnoNAC56_1_R | CCACTTCCTCCCCTCCGA | 59.961 | 66.667 | 83 |
| DnoNAC57 | DnoNAC57_1_F | gtgGGCTTCTGCGATGGT | 60.046 | 61.111 | 123 |
| DnoNAC57 | DnoNAC57_1_R | GTGAACAGTGAgccgcca | 60.281 | 61.111 | 123 |
| DnoNAC58 | DnoNAC58_1_F | TCTGGTCAGGCTTCCCCC | 60.611 | 66.667 | 116 |
| DnoNAC58 | DnoNAC58_1_R | TCTCGGGAATGATGGCGG | 59.178 | 61.111 | 116 |
| DnoNAC59 | DnoNAC59_1_F | GTCAGCCTCGCCAACCAT | 60.046 | 61.111 | 138 |
| DnoNAC59 | DnoNAC59_1_R | GGTCGGCAGTTCGGAGAG | 59.818 | 66.667 | 138 |
| DnoNAC6 | DnoNAC6_1_F | CGAGGCTTTCTTCGGCGA | 60.126 | 61.111 | 93 |
| DnoNAC6 | DnoNAC6_1_R | ACCCCGGCAGCCCTATTA | 60.04 | 61.111 | 93 |
| DnoNAC60 | DnoNAC60_1_F | GCTGCCGCCATGAGGAAT | 60.518 | 61.111 | 87 |
| DnoNAC60 | DnoNAC60_1_R | CGTTGGGTGGAAGCGGAA | 60.281 | 61.111 | 87 |
| DnoNAC61 | DnoNAC61_1_F | TCGCTCGTACCAGGTCCA | 59.965 | 61.111 | 124 |
| DnoNAC61 | DnoNAC61_1_R | AGGTCTAGGGCGGAGTGG | 60.043 | 66.667 | 124 |
| DnoNAC62 | DnoNAC62_1_F | GCTCCAGCTGAACGGCAA | 60.67 | 61.111 | 81 |
| DnoNAC62 | DnoNAC62_1_R | ggtgaacagtgcaacgcc | 59.669 | 61.111 | 81 |
| DnoNAC63 | DnoNAC63_1_F | ACGCGATGCCCACAAGAA | 59.968 | 55.556 | 93 |
| DnoNAC63 | DnoNAC63_1_R | CGACGACGGCGATCATGA | 59.976 | 61.111 | 93 |
| DnoNAC64 | DnoNAC64_1_F | TCGCCTCCATCTTCCCGA | 60.043 | 61.111 | 109 |
| DnoNAC64 | DnoNAC64_1_R | CGAGCACAGGTCCTTGGG | 60.047 | 66.667 | 109 |
| DnoNAC65 | DnoNAC65_1_F | CTTCAGCCCTCGTGACCG | 60.126 | 66.667 | 95 |
| DnoNAC65 | DnoNAC65_1_R | TCGGTTTGTCGGTTCCGG | 59.971 | 61.111 | 95 |
| DnoNAC66 | DnoNAC66_1_F | CCCCTTAGACCCCCGACA | 59.96 | 66.667 | 83 |
| DnoNAC66 | DnoNAC66_1_R | GAACAGGGGCGGAACCAA | 59.886 | 61.111 | 83 |
| DnoNAC67 | DnoNAC67_1_F | CGTCTCCCTTGCCCGTTT | 59.968 | 61.111 | 135 |
| DnoNAC67 | DnoNAC67_1_R | TGAGCAACACTCCGGCTG | 59.969 | 61.111 | 135 |
| DnoNAC68 | DnoNAC68_1_F | TTCATCCAACCCGCCACC | 59.965 | 61.111 | 92 |
| DnoNAC68 | DnoNAC68_1_R | ACTGCTCGGCCTTGCAAT | 59.965 | 55.556 | 92 |
| DnoNAC69 | DnoNAC69_1_F | GTCGTGGTGGAAAGGCGA | 59.97 | 61.111 | 129 |
| DnoNAC69 | DnoNAC69_1_R | CTGGAGAGCCCAACGACG | 60.126 | 66.667 | 129 |
| DnoNAC7 | DnoNAC7_1_F | TCCGTTGATTCCGTCGGC | 60.126 | 61.111 | 129 |
| DnoNAC7 | DnoNAC7_1_R | TCTGCAGGCGAGAACAGC | 60.048 | 61.111 | 129 |
| DnoNAC70 | DnoNAC70_1_F | TGGCGAAGATGGGGGAGA | 59.96 | 61.111 | 112 |
| DnoNAC70 | DnoNAC70_1_R | CCGGTGGCCTTCCAGAAG | 60.046 | 66.667 | 112 |
| DnoNAC71 | DnoNAC71_1_F | TGCGCTTTCCAGAGGGTG | 59.967 | 61.111 | 126 |
| DnoNAC71 | DnoNAC71_1_R | GAGCGACTGAAGCAGGCT | 59.736 | 61.111 | 126 |
| DnoNAC72 | DnoNAC72_1_F | GCTCGGAGCTTTACGGCT | 59.815 | 61.111 | 121 |
| DnoNAC72 | DnoNAC72_1_R | TTCGGGGATTATGGCGGC | 59.888 | 61.111 | 121 |
| DnoNAC73 | DnoNAC73_1_F | ggtttggaggggaaggCC | 59.963 | 66.667 | 122 |
| DnoNAC73 | DnoNAC73_1_R | ACCGGCTACCACCATCCT | 59.959 | 61.111 | 122 |
| DnoNAC74 | DnoNAC74_1_F | CGCGCAAATAGAGCCACG | 59.679 | 61.111 | 120 |
| DnoNAC74 | DnoNAC74_1_R | CGGCCTGCCTTCATGGAA | 60.045 | 61.111 | 120 |
| DnoNAC75 | DnoNAC75_1_F | AAGGGCCTTCTTGACGCC | 59.966 | 61.111 | 137 |
| DnoNAC75 | DnoNAC75_1_R | GCCCTCGTGATCGCAAGT | 60.126 | 61.111 | 137 |
| DnoNAC76 | DnoNAC76_1_F | GAGCTCATTGGCCTGCCA | 60.045 | 61.111 | 89 |
| DnoNAC76 | DnoNAC76_1_R | GGGTCAACGGTGATCGCA | 60.048 | 61.111 | 89 |
| DnoNAC77 | DnoNAC77_1_F | CTACAACGGTCGGGCTCC | 59.816 | 66.667 | 82 |
| DnoNAC77 | DnoNAC77_1_R | GGTGGTGGCCTGGTCTTC | 59.966 | 66.667 | 82 |
| DnoNAC78 | DnoNAC78_1_F | CCGGCGTCCTGAAACTGT | 59.969 | 61.111 | 95 |
| DnoNAC78 | DnoNAC78_1_R | TTGGGTGCTGTGCCGATT | 59.885 | 55.556 | 95 |
| DnoNAC79 | DnoNAC79_1_F | TTCCTTCTGCCTCCGGGA | 59.878 | 61.111 | 140 |
| DnoNAC79 | DnoNAC79_1_R | TCCCAAGGCTCGCACTTG | 59.967 | 61.111 | 140 |
| DnoNAC8 | DnoNAC8_1_F | TCCGAGCTTGCTCACTGC | 60.048 | 61.111 | 118 |
| DnoNAC8 | DnoNAC8_1_R | TGAGCTCATCGAGTGCGC | 60.203 | 61.111 | 118 |
| DnoNAC80 | DnoNAC80_1_F | AGGCTTCCGCTTTCACCC | 59.966 | 61.111 | 130 |
| DnoNAC80 | DnoNAC80_1_R | TTCCCACGGCTCGCATTT | 59.967 | 55.556 | 130 |
| DnoNAC81 | DnoNAC81_1_F | GTTTCGGCGCAGTTTCCG | 60.127 | 61.111 | 84 |
| DnoNAC81 | DnoNAC81_1_R | taaggggccggaattcGC | 59.809 | 61.111 | 84 |
| DnoNAC82 | DnoNAC82_1_F | CGGGGCTTCGTCCTCATC | 59.893 | 66.667 | 107 |
| DnoNAC82 | DnoNAC82_1_R | AGGCCGCGAACCTACAAC | 60.048 | 61.111 | 107 |
| DnoNAC83 | DnoNAC83_1_F | ACCGAGCTTGAAGAACCTTCA | 59.584 | 47.619 | 102 |
| DnoNAC83 | DnoNAC83_1_R | GCATTGCAGGTGATGGCG | 59.896 | 61.111 | 102 |
| DnoNAC84 | DnoNAC84_1_F | ATCAACCACCGGCACCTG | 59.966 | 61.111 | 115 |
| DnoNAC84 | DnoNAC84_1_R | GGAGATGCAGGTGGAGGC | 59.808 | 66.667 | 115 |
| DnoNAC85 | DnoNAC85_1_F | GTCAACCTCCGCCGTTCA | 59.97 | 61.111 | 107 |
| DnoNAC85 | DnoNAC85_1_R | CTGGGCATGGGGACGAAG | 60.125 | 66.667 | 107 |
| DnoNAC9 | DnoNAC9_1_F | CGATCTGCCCAAGCCCAT | 59.806 | 61.111 | 83 |
| DnoNAC9 | DnoNAC9_1_R | TGCAACCCAGTCAAGGCT | 59.074 | 55.556 | 83 |

Supplementary Table2. Hydrophilicity/hydrophobicity analysis of NAC proteins in *Dendrobium nobile*

| Protein name | Maximum hydrophobicity | | | Maximum hydrophilicity | | |
| --- | --- | --- | --- | --- | --- | --- |
|  | Position | amino acid | Value | Position | amino acid | Value |
| DnoNAC01 | 235 | A | 1.644 | 212 | N | -2.856 |
| DnoNAC02 | 244 | A | 2.333 | 302 | E | -3.8 |
| DnoNAC03 | 244 | L | 1.911 | 128 | P | -2.322 |
| DnoNAC04 | 567 | I | 2.433 | 615 | E | -2.944 |
| DnoNAC05 | 36 | T | 1.7 | 164 | P | -2.5 |
| DnoNAC06 | 269 | V | 1.8 | 200 | K | -3.244 |
| DnoNAC07 | 239 | A | 1.533 | 77 | R | -2.8 |
| DnoNAC08 | 264 | M | 1.711 | 78 | R | -2.8 |
| DnoNAC09 | 37 | V | 1.867 | 180 | K | -3.167 |
| DnoNAC10 | 44 | V | 1.622 | 74 | R | -2.933 |
| DnoNAC11 | 620 | G | 2.833 | 156 | L | -2.522 |
| DnoNAC12 | 239 | G | 1.489 | 178 | P | -3.189 |
| DnoNAC13 | 187 | I | 1.367 | 271 | Q | -3.2 |
| DnoNAC14 | 44 | I | 1.822 | 28 | D | -3.344 |
| DnoNAC15 | 176 | Y | 1.956 | 136 | R | -2.956 |
| DnoNAC16 | 210 | S | 1.567 | 135 | R | -2.967 |
| DnoNAC17 | 118 | Y | 1.056 | 160 | E | -3.856 |
| DnoNAC18 | 55 | T | 1.689 | 89 | R | -3.289 |
| DnoNAC19 | 246 | S | 1.156 | 269270 | C,K | -2.733 |
| DnoNAC20 | 292 | S | 1.456 | 79 | K | -3.122 |
| DnoNAC21 | 248 | V | 2.844 | 796 | R | -3.822 |
| DnoNAC22 | 38 | V | 1.189 | 254255 | Y,G | -3.067 |
| DnoNAC23 | 182 | D | 1.489 | 139141 | L,R | -2.4 |
| DnoNAC24 | 254 | A | 1.956 | 196197 | R,S | -2.822 |
| DnoNAC25 | 82 | G | 1.622 | 353357358 | H,Q,H | -3.433 |
| DnoNAC26 | 82 | F | 1.8 | 142 | T | -2.678 |
| DnoNAC27 | 47 | I | 1.944 | 67 | F | -1.911 |
| DnoNAC28 | 47 | I | 1.944 | 190 | A | -2.844 |
| DnoNAC29 | 332 | P | 1.8 | 75 | R | -2.8 |
| DnoNAC30 | 76 | A | 1.767 | 317 | H | -3.633 |
| DnoNAC31 | 332 | P | 1.8 | 75 | R | -2.8 |
| DnoNAC32 | 276 | Q | 1.589 | 129 | G | -2.289 |
| DnoNAC33 | 249 | L | 1.822 | 73 | K | -3.122 |
| DnoNAC34 | 10 | H | 1.367 | 136 | P | -2.389 |
| DnoNAC35 | 252 | N | 1.767 | 159 | Q | -3.333 |
| DnoNAC36 | 235249 | W,E | 0.833 | 160 | E | -3.056 |
| DnoNAC37 | 574 | L | 3.322 | 547 | N | -3.589 |
| DnoNAC38 | 44 | L | 1.611 | 74 | R | -2.944 |
| DnoNAC39 | 178 | S | 1.133 | 187 | K | -2.956 |
| DnoNAC40 | 196 | S | 1.133 | 205 | K | -2.956 |
| DnoNAC41 | 553 | L | 1.878 | 458 | D | -2.844 |
| DnoNAC42 | 656 | S | 2.611 | 303 | E | -2.8 |
| DnoNAC43 | 296 | D | 1.267 | 78 | K | -2.489 |
| DnoNAC44 | 143 | G | 2.233 | 70 | K | -2.644 |
| DnoNAC45 | 62 | I | 1.122 | 53 | D | -2.833 |
| DnoNAC46 | 117 | A | 2.033 | 176 | D | -2.567 |
| DnoNAC47 | 38 | A | 1.256 | 72 | K | -2.589 |
| DnoNAC48 | 111 | K | 1.456 | 147 | A | -3.022 |
| DnoNAC49 | 44 | I | 1.811 | 242243 | K,E | -3.444 |
| DnoNAC50 | 5 | S | 1.444 | 260 | Q | -2.311 |
| DnoNAC51 | 252 | L | 1.322 | 73 | K | -2.589 |
| DnoNAC52 | 292 | S | 1.522 | 79 | K | -3.122 |
| DnoNAC53 | 292 | S | 1.522 | 79 | K | -3.122 |
| DnoNAC54 | 38 | I | 1.533 | 211 | E | -2.944 |
| DnoNAC55 | 37 | V | 2.433 | 85 | R | -2.8 |
| DnoNAC56 | 178 | D | 1.456 | 77 | R | -2.9 |
| DnoNAC57 | 263 | L | 1.622 | 159 | G | -2.333 |
| DnoNAC58 | 42 | P | 1.689 | 182 | H | -3.356 |
| DnoNAC59 | 558 | F | 3.244 | 206 | E | -2.867 |
| DnoNAC60 | 56 | T | 1.689 | 90 | R | -3.289 |
| DnoNAC61 | 168 | C | 1.611 | 6 | N | -3.311 |
| DnoNAC62 | 101 | K | 1.211 | 212 | D | -3.2 |
| DnoNAC63 | 32 | I | 1.356 | 12 | G | -3.233 |
| DnoNAC64 | 119 | F | 1.5 | 139 | R | -3.078 |
| DnoNAC65 | 41 | A | 2.089 | 72 | R | -2.8 |
| DnoNAC66 | 318 | L | 2.633 | 72 | K | -2.589 |
| DnoNAC67 | 39 | I | 1.9 | 72 | R | -2.8 |
| DnoNAC68 | 172 | G | 1.656 | 75 | K | -2.656 |
| DnoNAC69 | 247 | L | 1.233 | 144 | D | -2.567 |
| DnoNAC70 | 44 | L | 1.611 | 74 | R | -2.689 |
| DnoNAC71 | 375 | L | 2.2 | 352 | R | -3.089 |
| DnoNAC72 | 42 | P | 1.689 | 207208 | R,D | -2.478 |
| DnoNAC73 | 144 | C | 1.289 | 295 | S | -2.867 |
| DnoNAC74 | 418 | I | 2.478 | 391392 | K,Q | -3.156 |
| DnoNAC75 | 239 | D | 1.744 | 76 | R | -2.8 |
| DnoNAC76 | 333 | F | 1.622 | 281 | P | -3.144 |
| DnoNAC77 | 41 | A | 1.756 | 129 | W | -2.433 |
| DnoNAC78 | 44,45 | P,I | 1.767 | 169 | K | -2.867 |
| DnoNAC79 | 43 | A | 1.5 | 77 | K | -2.656 |
| DnoNAC80 | 586 | A | 3.6 | 329 | D | -3.311 |
| DnoNAC81 | 230 | V | 1.367 | 142 | T | -2.844 |
| DnoNAC82 | 151 | V | 0.956 | 63 | D | -2.989 |
| DnoNAC83 | 111 | K | 1 | 63 | D | -2.922 |
| DnoNAC84 | 44 | I | 1.811 | 168 | N | -3.1 |
| DnoNAC85 | 41 | A | 1.5 | 72 | R | -2.8 |

Supplementary Table 3. Secondary structure analysis of NAC proteins from *Dendrobium nobile*

| Protein | Alpha helix | | Extended strand | | Beta turn | | Random coil | |
| --- | --- | --- | --- | --- | --- | --- | --- | --- |
|  | Value | Proporttion | Value | Proporttion | Value | Proporttion | Value | Proporttion |
| DnoNAC1 | 68 | 21.32% | 46 | 14.42% | 9 | 2.82% | 196 | 61.44% |
| DnoNAC2 | 108 | 25.47% | 54 | 12.74% | 26 | 6.13% | 236 | 55.66% |
| DnoNAC3 | 38 | 13.38% | 46 | 16.20% | 8 | 2.82% | 192 | 67.61% |
| DnoNAC4 | 197 | 29.80% | 73 | 11.04% | 27 | 4.08% | 364 | 55.07% |
| DnoNAC5 | 65 | 19.94% | 54 | 16.56% | 10 | 3.07% | 197 | 60.43% |
| DnoNAC6 | 66 | 20.62% | 40 | 12.50% | 10 | 3.12% | 204 | 63.75% |
| DnoNAC7 | 72 | 22.64% | 37 | 11.64% | 13 | 4.09% | 196 | 61.64% |
| DnoNAC8 | 76 | 24.05% | 36 | 11.39% | 13 | 4.11% | 191 | 60.44% |
| DnoNAC9 | 76 | 21.05% | 45 | 12.47% | 9 | 2.49% | 231 | 63.99% |
| DnoNAC10 | 84 | 29.58% | 36 | 12.68% | 11 | 3.87% | 153 | 53.87% |
| DnoNAC11 | 155 | 24.33% | 88 | 13.81% | 26 | 4.08% | 368 | 57.77% |
| DnoNAC12 | 74 | 19.27% | 58 | 15.10% | 18 | 4.69% | 234 | 60.94% |
| DnoNAC13 | 63 | 22.26% | 46 | 16.25% | 8 | 2.83% | 166 | 58.66% |
| DnoNAC14 | 40 | 14.13% | 49 | 17.31% | 17 | 6.01% | 177 | 62.54% |
| DnoNAC15 | 106 | 24.77% | 63 | 14.72% | 21 | 4.91% | 238 | 55.61% |
| DnoNAC16 | 81 | 20.93% | 59 | 13.25% | 17 | 4.39% | 230 | 59.43% |
| DnoNAC17 | 60 | 22.06% | 54 | 19.85% | 20 | 7.35% | 138 | 50.74% |
| DnoNAC18 | 65 | 20.25% | 39 | 12.15% | 13 | 4.05% | 204 | 63.55% |
| DnoNAC19 | 56 | 18.06% | 50 | 16.13% | 20 | 6.45% | 184 | 59.35% |
| DnoNAC20 | 68 | 17.09% | 25 | 6.28% | 12 | 3.02% | 293 | 73.62% |
| DnoNAC21 | 361 | 33.90% | 209 | 19.62% | 84 | 7.89% | 411 | 38.59% |
| DnoNAC22 | 49 | 16.33% | 37 | 12.33% | 13 | 4.33% | 201 | 67.00% |
| DnoNAC23 | 45 | 22.73% | 36 | 18.18% | 9 | 4.55% | 108 | 54.55% |
| DnoNAC24 | 105 | 23.54% | 77 | 17.26% | 39 | 8.74% | 225 | 50.45% |
| DnoNAC25 | 107 | 27.09% | 60 | 15.19% | 22 | 5.57% | 206 | 52.15% |
| DnoNAC26 | 35 | 15.32% | 44 | 19.82% | 17 | 7.66% | 127 | 57.21% |
| DnoNAC27 | 11 | 13.75% | 17 | 21.25% | 1 | 1.25% | 51 | 63.75% |
| DnoNAC28 | 81 | 23.01% | 38 | 10.80% | 12 | 3.41% | 221 | 62.78% |
| DnoNAC29 | 73 | 20.98% | 44 | 12.64% | 12 | 3.45% | 219 | 62.93% |
| DnoNAC30 | 59 | 16.25% | 45 | 12.40% | 7 | 1.93% | 252 | 69.42% |
| DnoNAC31 | 56 | 16.09% | 51 | 14.66% | 19 | 5.46% | 222 | 63.79% |
| DnoNAC32 | 66 | 21.09% | 43 | 13.74% | 17 | 5.43% | 187 | 59.74% |
| DnoNAC33 | 55 | 18.09% | 40 | 13.16% | 11 | 3.62% | 198 | 65.13% |
| DnoNAC34 | 43 | 12.84% | 56 | 16.72% | 16 | 4.78% | 220 | 65.67% |
| DnoNAC35 | 67 | 25.57% | 48 | 18.32% | 10 | 3.82% | 137 | 52.29% |
| DnoNAC36 | 37 | 14.02% | 57 | 21.59% | 18 | 6.82% | 152 | 57.58% |
| DnoNAC37 | 159 | 27.18% | 78 | 13.33% | 24 | 4.10% | 324 | 55.38% |
| DnoNAC38 | 77 | 24.60% | 36 | 11.50% | 10 | 3.19% | 190 | 60.70% |
| DnoNAC39 | 38 | 17.43% | 29 | 13.30% | 7 | 3.21% | 144 | 66.06% |
| DnoNAC40 | 32 | 13.56% | 34 | 14.41% | 12 | 5.08% | 158 | 66.95% |
| DnoNAC41 | 158 | 27.92% | 65 | 11.48% | 18 | 3.18% | 325 | 57.42% |
| DnoNAC42 | 146 | 21.57% | 84 | 12.41% | 21 | 3.10% | 426 | 62.92% |
| DnoNAC43 | 94 | 26.48% | 47 | 13.24% | 18 | 5.07% | 196 | 55.21% |
| DnoNAC44 | 60 | 18.35% | 49 | 14.98% | 12 | 3.67% | 206 | 63.00% |
| DnoNAC45 | 86 | 27.92% | 43 | 13.96% | 5 | 1.62% | 174 | 56.49% |
| DnoNAC46 | 57 | 17.22% | 57 | 17.22% | 17 | 5.14% | 200 | 60.42% |
| DnoNAC47 | 70 | 22.51% | 41 | 13.18% | 10 | 3.22% | 190 | 61.09% |
| DnoNAC48 | 62 | 18.73% | 39 | 11.78% | 7 | 2.11% | 223 | 67.37% |
| DnoNAC49 | 71 | 23.67% | 31 | 10.33% | 9 | 3.00% | 189 | 63.00% |
| DnoNAC50 | 69 | 24.38% | 36 | 12.72% | 12 | 4.24% | 166 | 58.66% |
| DnoNAC51 | 75 | 25.25% | 53 | 17.85% | 12 | 4.04% | 157 | 52.86% |
| DnoNAC52 | 67 | 16.83% | 27 | 6.78% | 14 | 3.52% | 290 | 72.86% |
| DnoNAC53 | 66 | 16.58% | 28 | 7.04% | 14 | 3.52% | 290 | 72.86% |
| DnoNAC54 | 40 | 14.55% | 50 | 18.18% | 10 | 3.64% | 175 | 63.64% |
| DnoNAC55 | 58 | 18.53% | 34 | 10.86% | 7 | 2.24% | 214 | 68.37% |
| DnoNAC56 | 52 | 26.80% | 36 | 18.56% | 9 | 4.64% | 97 | 50.00% |
| DnoNAC57 | 52 | 17.22% | 55 | 18.21% | 21 | 6.95% | 174 | 57.62% |
| DnoNAC58 | 45 | 20.55% | 40 | 18.26% | 11 | 5.02% | 123 | 56.16% |
| DnoNAC59 | 160 | 27.59% | 71 | 12.24% | 18 | 3.10% | 331 | 57.07% |
| DnoNAC60 | 74 | 21.64% | 38 | 11.11% | 14 | 4.09% | 216 | 63.16% |
| DnoNAC61 | 51 | 15.74% | 36 | 11.11% | 14 | 4.32% | 223 | 68.83% |
| DnoNAC62 | 86 | 26.76% | 53 | 16.51% | 15 | 4.67% | 167 | 52.02% |
| DnoNAC63 | 45 | 15.36% | 58 | 19.80% | 13 | 4.44% | 177 | 60.41% |
| DnoNAC64 | 79 | 20.47% | 50 | 12.95% | 10 | 2.59% | 247 | 63.99% |
| DnoNAC65 | 69 | 21.17% | 40 | 12.27% | 10 | 3.07% | 207 | 63.50% |
| DnoNAC66 | 77 | 19.85% | 69 | 17.78% | 27 | 6.96% | 215 | 55.41% |
| DnoNAC67 | 61 | 18.89% | 35 | 10.84% | 11 | 3.41% | 216 | 66.87% |
| DnoNAC68 | 71 | 22.33% | 44 | 13.84% | 9 | 2.83% | 194 | 61.01% |
| DnoNAC69 | 99 | 26.26% | 48 | 12.73% | 18 | 4.77% | 212 | 56.23% |
| DnoNAC70 | 83 | 26.02% | 34 | 10.66% | 6 | 1.88% | 196 | 61.44% |
| DnoNAC71 | 118 | 30.75% | 61 | 15.80% | 18 | 4.66% | 189 | 48.96% |
| DnoNAC72 | 42 | 16.80% | 45 | 18.00% | 13 | 5.20% | 150 | 60.00% |
| DnoNAC73 | 71 | 18.93% | 66 | 17.60% | 10 | 2.67% | 228 | 60.80% |
| DnoNAC74 | 110 | 2517.50% | 63 | 14.24% | 22 | 5.03% | 242 | 55.38% |
| DnoNAC75 | 51 | 20.65% | 45 | 18.22% | 13 | 5.26% | 138 | 55.87% |
| DnoNAC76 | 95 | 23.75% | 54 | 13.50% | 19 | 4.75% | 232 | 58.00% |
| DnoNAC77 | 54 | 26.77% | 37 | 18.32% | 12 | 5.94% | 99 | 49.01% |
| DnoNAC78 | 65 | 26.42% | 35 | 14.23% | 8 | 3.25% | 138 | 56.10% |
| DnoNAC79 | 61 | 17.68% | 44 | 12.75% | 14 | 4.06% | 226 | 65.51% |
| DnoNAC80 | 200 | 33.28% | 82 | 13.64% | 18 | 3.00% | 301 | 50.08% |
| DnoNAC81 | 52 | 16.61% | 42 | 13.42% | 11 | 3.51% | 208 | 66.45% |
| DnoNAC82 | 56 | 21.37% | 43 | 16.41% | 11 | 4.20% | 152 | 58.02% |
| DnoNAC83 | 59 | 20.63% | 45 | 15.73% | 13 | 4.55% | 169 | 59.09% |
| DnoNAC84 | 66 | 24.09% | 44 | 16.06% | 9 | 3.28% | 155 | 56.57% |
| DnoNAC85 | 60 | 18.02% | 32 | 9.61% | 12 | 3.60% | 229 | 68.77% |

Supplementary Table 4. Base compositions and related codon parameters of *NAC* family members in *Dendrobium nobile*

| gene | T3s | C3s | A3s | G3s | CAI | CBI | Fop | ENc | GC3s | GC | L_sym | L_aa | Aromo |
| --- | --- | --- | --- | --- | --- | --- | --- | --- | --- | --- | --- | --- | --- |
| DnoNAC1 | 0.44 | 0.23 | 0.45 | 0.17 | 0.16 | -0.13 | 0.33 | 49.67 | 0.30 | 0.33 | 1596 | 1645 | 0.13 |
| DnoNAC2 | 0.40 | 0.20 | 0.43 | 0.24 | 0.14 | -0.14 | 0.34 | 50.72 | 0.33 | 0.38 | 1274 | 1320 | 0.12 |
| DnoNAC3 | 0.35 | 0.20 | 0.34 | 0.32 | 0.17 | -0.08 | 0.38 | 56.46 | 0.42 | 0.46 | 357 | 371 | 0.12 |
| DnoNAC4 | 0.40 | 0.24 | 0.41 | 0.21 | 0.16 | -0.10 | 0.35 | 52.09 | 0.35 | 0.40 | 7168 | 7394 | 0.12 |
| DnoNAC5 | 0.38 | 0.25 | 0.38 | 0.27 | 0.15 | -0.16 | 0.31 | 53.63 | 0.40 | 0.40 | 455 | 473 | 0.12 |
| DnoNAC6 | 0.34 | 0.28 | 0.33 | 0.33 | 0.20 | 0.00 | 0.42 | 58.69 | 0.47 | 0.46 | 361 | 376 | 0.11 |
| DnoNAC7 | 0.36 | 0.29 | 0.34 | 0.24 | 0.17 | -0.03 | 0.40 | 56.20 | 0.43 | 0.46 | 364 | 377 | 0.12 |
| DnoNAC8 | 0.31 | 0.34 | 0.29 | 0.30 | 0.19 | -0.01 | 0.40 | 56.04 | 0.51 | 0.48 | 346 | 363 | 0.09 |
| DnoNAC9 | 0.44 | 0.20 | 0.41 | 0.22 | 0.17 | -0.12 | 0.35 | 50.33 | 0.32 | 0.38 | 695 | 722 | 0.12 |
| DnoNAC10 | 0.39 | 0.24 | 0.47 | 0.21 | 0.15 | -0.16 | 0.33 | 51.60 | 0.33 | 0.36 | 954 | 974 | 0.13 |
| DnoNAC11 | 0.42 | 0.21 | 0.46 | 0.21 | 0.17 | -0.13 | 0.34 | 50.05 | 0.31 | 0.36 | 1714 | 1779 | 0.11 |
| DnoNAC12 | 0.46 | 0.22 | 0.44 | 0.19 | 0.19 | -0.09 | 0.37 | 49.03 | 0.30 | 0.33 | 1748 | 1794 | 0.14 |
| DnoNAC13 | 0.35 | 0.27 | 0.34 | 0.32 | 0.17 | -0.05 | 0.38 | 51.48 | 0.45 | 0.40 | 314 | 331 | 0.11 |
| DnoNAC14 | 0.46 | 0.20 | 0.46 | 0.18 | 0.15 | -0.15 | 0.33 | 46.93 | 0.28 | 0.32 | 1771 | 1817 | 0.14 |
| DnoNAC15 | 0.38 | 0.26 | 0.38 | 0.24 | 0.16 | -0.08 | 0.37 | 56.61 | 0.39 | 0.40 | 4155 | 4319 | 0.12 |
| DnoNAC16 | 0.44 | 0.23 | 0.43 | 0.21 | 0.17 | -0.11 | 0.35 | 53.03 | 0.32 | 0.36 | 1620 | 1658 | 0.13 |
| DnoNAC17 | 0.43 | 0.22 | 0.47 | 0.16 | 0.15 | -0.14 | 0.33 | 47.54 | 0.29 | 0.32 | 862 | 884 | 0.16 |
| DnoNAC18 | 0.37 | 0.27 | 0.29 | 0.31 | 0.22 | 0.05 | 0.45 | 53.81 | 0.45 | 0.44 | 440 | 455 | 0.10 |
| DnoNAC19 | 0.28 | 0.33 | 0.34 | 0.28 | 0.18 | 0.00 | 0.41 | 52.12 | 0.48 | 0.46 | 345 | 358 | 0.10 |
| DnoNAC20 | 0.40 | 0.23 | 0.43 | 0.19 | 0.18 | -0.06 | 0.38 | 50.32 | 0.33 | 0.39 | 719 | 751 | 0.11 |
| DnoNAC21 | 0.39 | 0.25 | 0.42 | 0.21 | 0.16 | -0.10 | 0.36 | 53.39 | 0.35 | 0.38 | 12736 | 13179 | 0.11 |
| DnoNAC22 | 0.40 | 0.25 | 0.41 | 0.19 | 0.19 | -0.04 | 0.39 | 54.99 | 0.34 | 0.38 | 567 | 589 | 0.11 |
| DnoNAC23 | 0.36 | 0.23 | 0.33 | 0.29 | 0.18 | -0.09 | 0.37 | 56.39 | 0.42 | 0.46 | 254 | 266 | 0.09 |
| DnoNAC24 | 0.40 | 0.26 | 0.39 | 0.21 | 0.18 | -0.05 | 0.38 | 54.89 | 0.37 | 0.38 | 1796 | 1845 | 0.11 |
| DnoNAC25 | 0.44 | 0.22 | 0.44 | 0.19 | 0.15 | -0.14 | 0.33 | 51.46 | 0.30 | 0.33 | 10242 | 10575 | 0.14 |
| DnoNAC26 | 0.40 | 0.28 | 0.33 | 0.21 | 0.19 | -0.03 | 0.39 | 53.72 | 0.39 | 0.41 | 403 | 414 | 0.12 |
| DnoNAC27 | 0.36 | 0.19 | 0.36 | 0.28 | 0.11 | -0.08 | 0.37 | 55.43 | 0.38 | 0.50 | 97 | 100 | 0.08 |
| DnoNAC28 | 0.23 | 0.26 | 0.27 | 0.36 | 0.15 | 0.09 | 0.45 | 54.23 | 0.55 | 0.58 | 359 | 365 | 0.06 |
| DnoNAC29 | 0.36 | 0.28 | 0.33 | 0.33 | 0.20 | -0.04 | 0.40 | 54.38 | 0.46 | 0.43 | 474 | 503 | 0.14 |
| DnoNAC30 | 0.39 | 0.27 | 0.37 | 0.24 | 0.18 | -0.03 | 0.39 | 57.42 | 0.39 | 0.40 | 737 | 759 | 0.13 |
| DnoNAC31 | 0.39 | 0.25 | 0.39 | 0.25 | 0.17 | -0.11 | 0.35 | 55.12 | 0.38 | 0.43 | 466 | 491 | 0.13 |
| DnoNAC32 | 0.41 | 0.24 | 0.44 | 0.20 | 0.15 | -0.13 | 0.34 | 52.95 | 0.33 | 0.36 | 4544 | 4685 | 0.12 |
| DnoNAC33 | 0.39 | 0.22 | 0.35 | 0.27 | 0.17 | -0.06 | 0.37 | 57.24 | 0.39 | 0.45 | 2109 | 2173 | 0.08 |
| DnoNAC34 | 0.35 | 0.28 | 0.38 | 0.26 | 0.16 | -0.04 | 0.39 | 51.61 | 0.42 | 0.44 | 357 | 369 | 0.10 |
| DnoNAC35 | 0.31 | 0.33 | 0.33 | 0.21 | 0.19 | 0.03 | 0.43 | 57.51 | 0.46 | 0.50 | 285 | 296 | 0.08 |
| DnoNAC36 | 0.44 | 0.19 | 0.52 | 0.19 | 0.16 | -0.13 | 0.34 | 45.56 | 0.27 | 0.29 | 1170 | 1199 | 0.14 |
| DnoNAC37 | 0.39 | 0.24 | 0.33 | 0.27 | 0.15 | -0.07 | 0.34 | 57.22 | 0.40 | 0.39 | 1048 | 1117 | 0.13 |
| DnoNAC38 | 0.39 | 0.28 | 0.35 | 0.20 | 0.18 | -0.06 | 0.38 | 57.27 | 0.39 | 0.44 | 414 | 426 | 0.11 |
| DnoNAC39 | 0.46 | 0.23 | 0.40 | 0.20 | 0.17 | -0.11 | 0.34 | 51.69 | 0.32 | 0.35 | 1133 | 1181 | 0.14 |
| DnoNAC40 | 0.43 | 0.25 | 0.41 | 0.20 | 0.17 | -0.10 | 0.36 | 53.31 | 0.34 | 0.37 | 1556 | 1609 | 0.14 |
| DnoNAC41 | 0.41 | 0.22 | 0.45 | 0.19 | 0.16 | -0.13 | 0.35 | 51.71 | 0.31 | 0.36 | 1105 | 1146 | 0.11 |
| DnoNAC42 | 0.39 | 0.24 | 0.36 | 0.26 | 0.16 | -0.11 | 0.34 | 56.17 | 0.39 | 0.42 | 6687 | 6949 | 0.12 |
| DnoNAC43 | 0.42 | 0.19 | 0.37 | 0.26 | 0.15 | -0.07 | 0.37 | 54.74 | 0.35 | 0.40 | 597 | 620 | 0.10 |
| DnoNAC44 | 0.42 | 0.28 | 0.37 | 0.24 | 0.20 | -0.02 | 0.41 | 52.06 | 0.39 | 0.38 | 396 | 414 | 0.15 |
| DnoNAC45 | 0.44 | 0.23 | 0.43 | 0.18 | 0.19 | -0.09 | 0.36 | 47.60 | 0.31 | 0.36 | 486 | 496 | 0.14 |
| DnoNAC46 | 0.41 | 0.26 | 0.38 | 0.22 | 0.18 | -0.07 | 0.37 | 54.71 | 0.37 | 0.39 | 908 | 930 | 0.13 |
| DnoNAC47 | 0.42 | 0.23 | 0.38 | 0.27 | 0.17 | -0.11 | 0.35 | 56.09 | 0.36 | 0.37 | 539 | 563 | 0.12 |
| DnoNAC48 | 0.33 | 0.33 | 0.32 | 0.25 | 0.18 | 0.03 | 0.42 | 56.58 | 0.46 | 0.46 | 354 | 367 | 0.10 |
| DnoNAC49 | 0.28 | 0.32 | 0.26 | 0.42 | 0.20 | -0.02 | 0.41 | 55.86 | 0.57 | 0.50 | 337 | 354 | 0.11 |
| DnoNAC50 | 0.36 | 0.25 | 0.42 | 0.25 | 0.16 | -0.08 | 0.37 | 54.68 | 0.38 | 0.40 | 785 | 805 | 0.11 |
| DnoNAC51 | 0.40 | 0.23 | 0.42 | 0.26 | 0.16 | -0.11 | 0.35 | 51.42 | 0.35 | 0.37 | 438 | 455 | 0.11 |
| DnoNAC52 | 0.36 | 0.27 | 0.39 | 0.24 | 0.17 | -0.03 | 0.39 | 52.25 | 0.40 | 0.40 | 622 | 649 | 0.11 |
| DnoNAC53 | 0.36 | 0.27 | 0.39 | 0.24 | 0.17 | -0.03 | 0.39 | 52.26 | 0.39 | 0.40 | 622 | 649 | 0.11 |
| DnoNAC54 | 0.45 | 0.22 | 0.40 | 0.20 | 0.18 | -0.10 | 0.36 | 50.35 | 0.32 | 0.35 | 671 | 691 | 0.12 |
| DnoNAC55 | 0.31 | 0.26 | 0.29 | 0.32 | 0.14 | 0.00 | 0.42 | 58.16 | 0.48 | 0.52 | 444 | 455 | 0.09 |
| DnoNAC56 | 0.34 | 0.29 | 0.35 | 0.27 | 0.18 | -0.08 | 0.37 | 53.77 | 0.44 | 0.44 | 235 | 241 | 0.10 |
| DnoNAC57 | 0.37 | 0.25 | 0.48 | 0.18 | 0.16 | -0.09 | 0.36 | 50.43 | 0.33 | 0.35 | 864 | 891 | 0.12 |
| DnoNAC58 | 0.36 | 0.29 | 0.35 | 0.27 | 0.18 | -0.05 | 0.39 | 53.56 | 0.43 | 0.41 | 273 | 284 | 0.15 |
| DnoNAC59 | 0.36 | 0.27 | 0.43 | 0.21 | 0.19 | -0.03 | 0.40 | 54.50 | 0.37 | 0.40 | 1151 | 1200 | 0.09 |
| DnoNAC60 | 0.45 | 0.24 | 0.37 | 0.21 | 0.19 | -0.09 | 0.37 | 50.93 | 0.34 | 0.36 | 725 | 754 | 0.19 |
| DnoNAC61 | 0.45 | 0.21 | 0.42 | 0.23 | 0.17 | -0.10 | 0.35 | 51.90 | 0.32 | 0.35 | 2299 | 2380 | 0.14 |
| DnoNAC62 | 0.43 | 0.25 | 0.37 | 0.21 | 0.21 | -0.02 | 0.40 | 51.35 | 0.36 | 0.39 | 497 | 510 | 0.14 |
| DnoNAC63 | 0.43 | 0.21 | 0.41 | 0.22 | 0.15 | -0.15 | 0.32 | 51.52 | 0.33 | 0.36 | 3300 | 3444 | 0.13 |
| DnoNAC64 | 0.43 | 0.23 | 0.36 | 0.24 | 0.17 | -0.09 | 0.35 | 55.22 | 0.36 | 0.39 | 1533 | 1594 | 0.13 |
| DnoNAC65 | 0.34 | 0.30 | 0.35 | 0.25 | 0.19 | 0.00 | 0.42 | 59.22 | 0.43 | 0.45 | 403 | 423 | 0.08 |
| DnoNAC66 | 0.44 | 0.21 | 0.41 | 0.21 | 0.15 | -0.13 | 0.33 | 51.99 | 0.32 | 0.35 | 2680 | 2779 | 0.14 |
| DnoNAC67 | 0.45 | 0.20 | 0.40 | 0.24 | 0.17 | -0.12 | 0.34 | 51.05 | 0.33 | 0.35 | 1957 | 2029 | 0.13 |
| DnoNAC68 | 0.38 | 0.22 | 0.34 | 0.28 | 0.18 | -0.01 | 0.41 | 56.53 | 0.40 | 0.44 | 493 | 507 | 0.10 |
| DnoNAC69 | 0.34 | 0.25 | 0.33 | 0.31 | 0.16 | -0.09 | 0.35 | 56.23 | 0.45 | 0.47 | 498 | 527 | 0.10 |
| DnoNAC70 | 0.29 | 0.26 | 0.28 | 0.40 | 0.19 | 0.03 | 0.43 | 55.27 | 0.53 | 0.50 | 362 | 382 | 0.11 |
| DnoNAC71 | 0.41 | 0.24 | 0.40 | 0.23 | 0.22 | -0.10 | 0.37 | 52.43 | 0.36 | 0.39 | 640 | 667 | 0.10 |
| DnoNAC72 | 0.45 | 0.22 | 0.43 | 0.21 | 0.18 | -0.10 | 0.37 | 52.75 | 0.32 | 0.35 | 569 | 591 | 0.16 |
| DnoNAC73 | 0.42 | 0.24 | 0.38 | 0.23 | 0.16 | -0.11 | 0.34 | 54.16 | 0.36 | 0.39 | 8454 | 8777 | 0.12 |
| DnoNAC74 | 0.38 | 0.28 | 0.34 | 0.28 | 0.20 | 0.00 | 0.41 | 55.64 | 0.42 | 0.41 | 654 | 689 | 0.11 |
| DnoNAC75 | 0.37 | 0.27 | 0.39 | 0.23 | 0.18 | -0.06 | 0.38 | 55.12 | 0.39 | 0.43 | 292 | 305 | 0.08 |
| DnoNAC76 | 0.41 | 0.24 | 0.42 | 0.19 | 0.17 | -0.09 | 0.36 | 52.93 | 0.33 | 0.36 | 10295 | 10596 | 0.12 |
| DnoNAC77 | 0.34 | 0.27 | 0.29 | 0.33 | 0.20 | 0.02 | 0.42 | 58.23 | 0.47 | 0.47 | 302 | 313 | 0.13 |
| DnoNAC78 | 0.27 | 0.42 | 0.26 | 0.27 | 0.20 | 0.12 | 0.49 | 52.82 | 0.56 | 0.51 | 295 | 306 | 0.09 |
| DnoNAC79 | 0.36 | 0.29 | 0.40 | 0.20 | 0.16 | -0.10 | 0.36 | 51.69 | 0.39 | 0.43 | 364 | 383 | 0.11 |
| DnoNAC80 | 0.41 | 0.22 | 0.41 | 0.25 | 0.15 | -0.15 | 0.33 | 51.95 | 0.35 | 0.37 | 9830 | 10261 | 0.13 |
| DnoNAC81 | 0.39 | 0.22 | 0.51 | 0.19 | 0.15 | -0.12 | 0.34 | 49.91 | 0.30 | 0.33 | 2099 | 2173 | 0.13 |
| DnoNAC82 | 0.50 | 0.20 | 0.35 | 0.21 | 0.18 | -0.10 | 0.35 | 49.77 | 0.31 | 0.36 | 1500 | 1556 | 0.16 |
| DnoNAC83 | 0.45 | 0.20 | 0.48 | 0.21 | 0.16 | -0.15 | 0.33 | 47.05 | 0.29 | 0.29 | 1173 | 1213 | 0.15 |
| DnoNAC84 | 0.16 | 0.47 | 0.34 | 0.22 | 0.21 | 0.15 | 0.50 | 47.67 | 0.58 | 0.51 | 316 | 319 | 0.10 |
| DnoNAC85 | 0.32 | 0.33 | 0.31 | 0.28 | 0.22 | 0.06 | 0.45 | 59.70 | 0.48 | 0.47 | 357 | 372 | 0.10 |
