# Supplementary material for: Genome-wide identification and molecular evolution of NAC gene family in Dendrobium nobile
Source: Front Plant Sci. 2023 Aug 21;14:1232804. doi: 10.3389/fpls.2023.1232804 (PMC10475575; doi:10.3389/fpls.2023.1232804)
Supplement: Supplementary file 1 [file DataSheet_1.zip › Data Sheet 1 - 2023-07-31T122136.933.PDF]

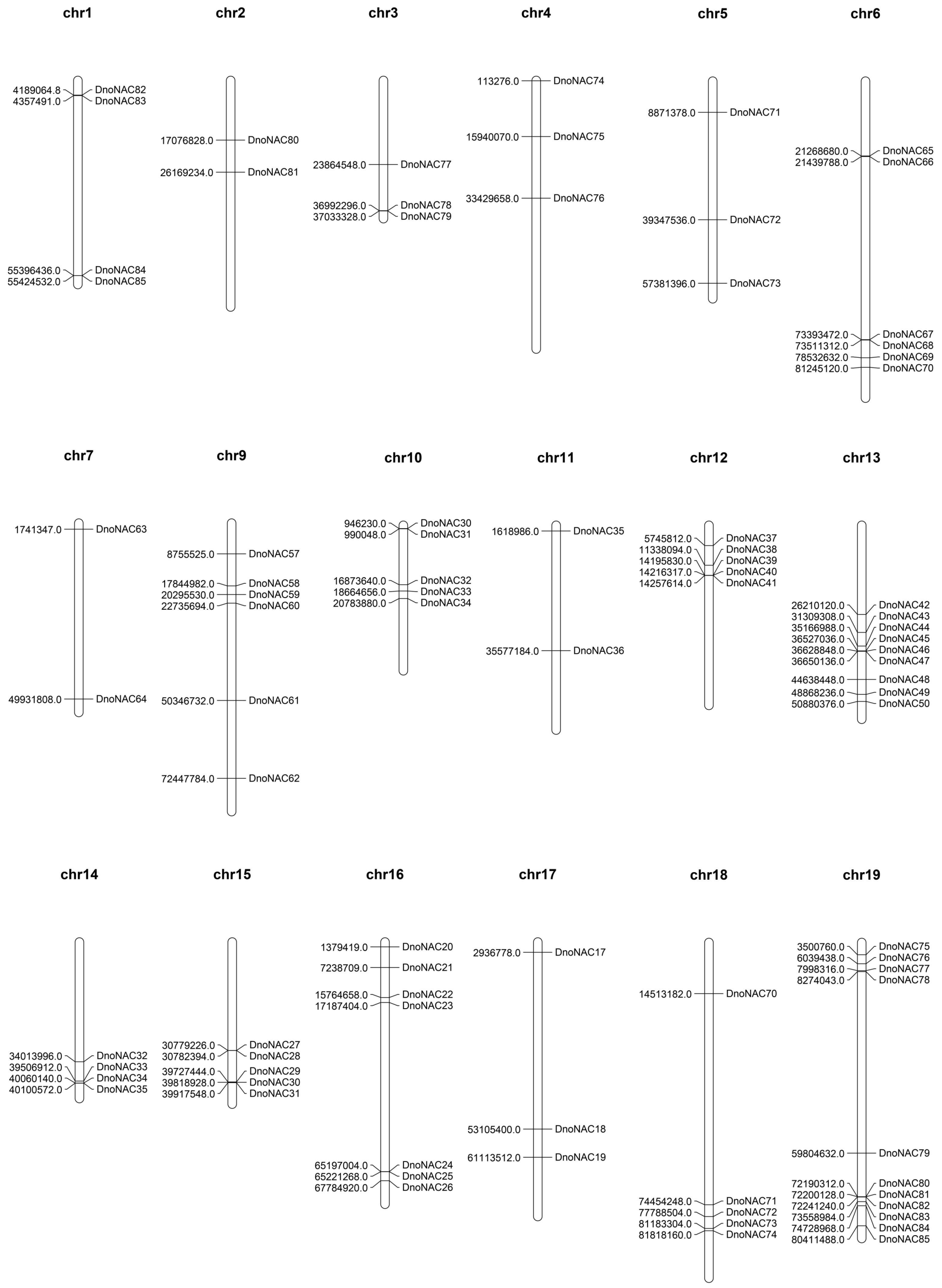

Supplementary Fig. 1. Chromosome mapping of *NAC* gene family in *Dendrobium nobile*

Motif name	Sequence logo	E-value
motif1	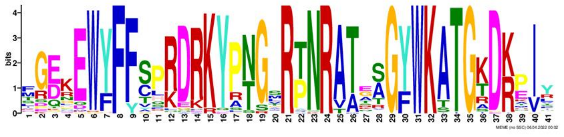	3.8e-2108
motif2	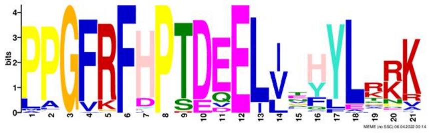	1.9e-1228
motif3	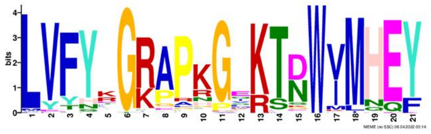	1.7e-1231
motif4	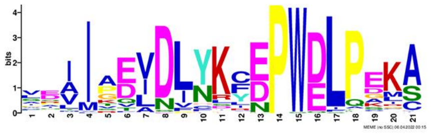	9.0e-786
motif5	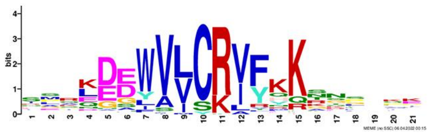	1.1e-469
motif6	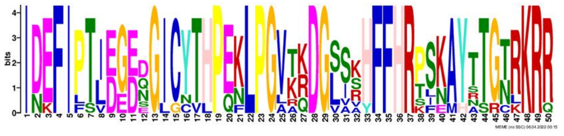	8.1e-235
motif7	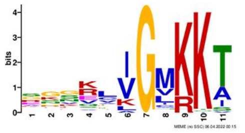	1.3e-212
motif8	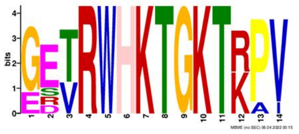	1.3e-050
motif9	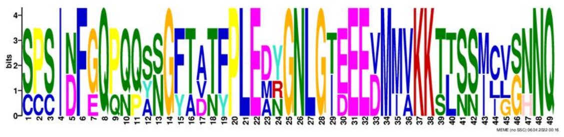	1.7e-049
motif10	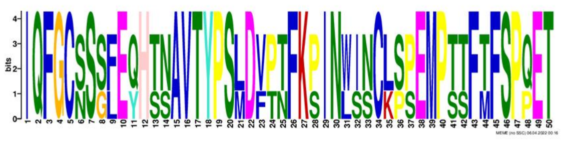	5.2e-036

Supplementary Fig. 2. Sequence logo of the conservative motifs

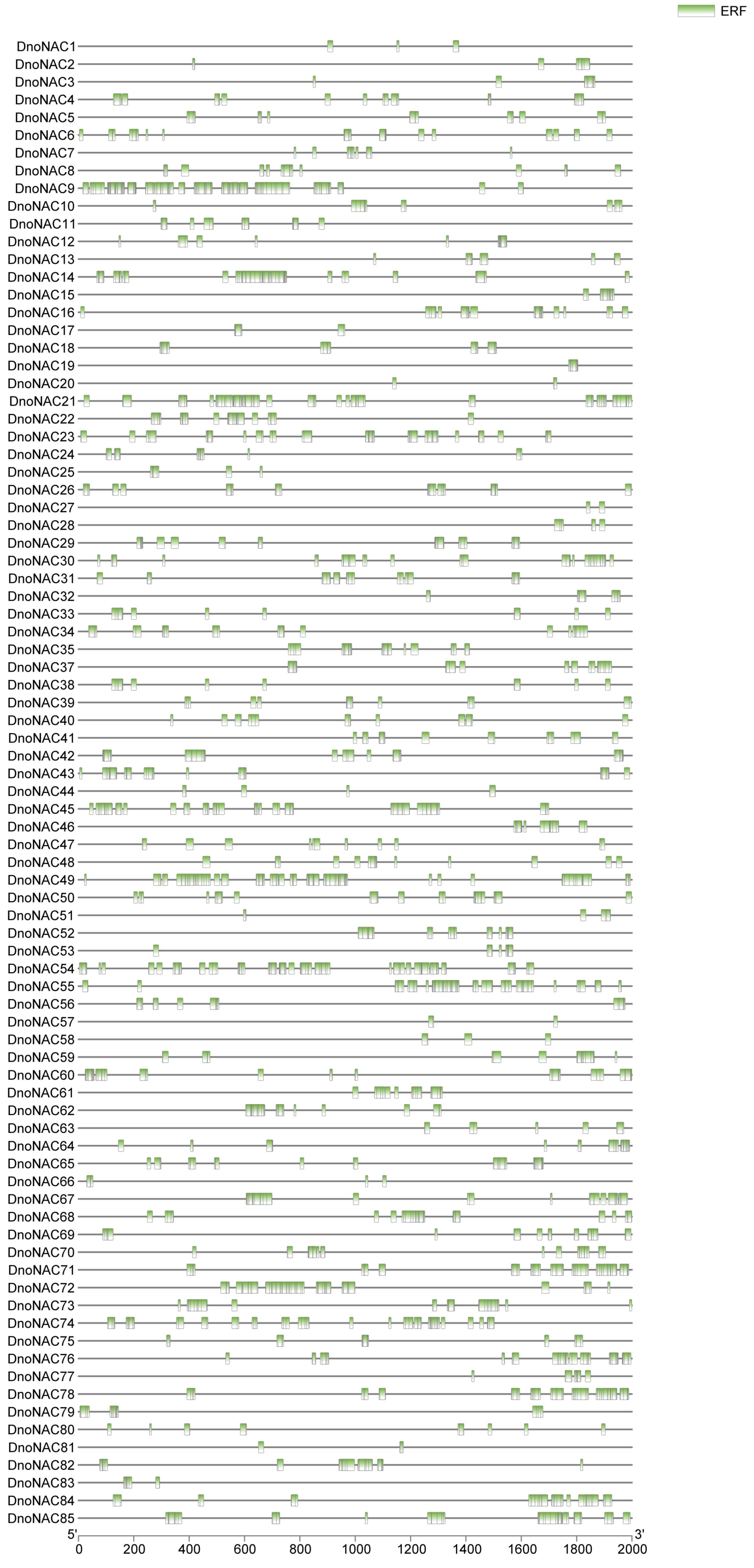

Supplementary Fig. 3. The ERF TF binding sites in the promoter region of the DnoNACs

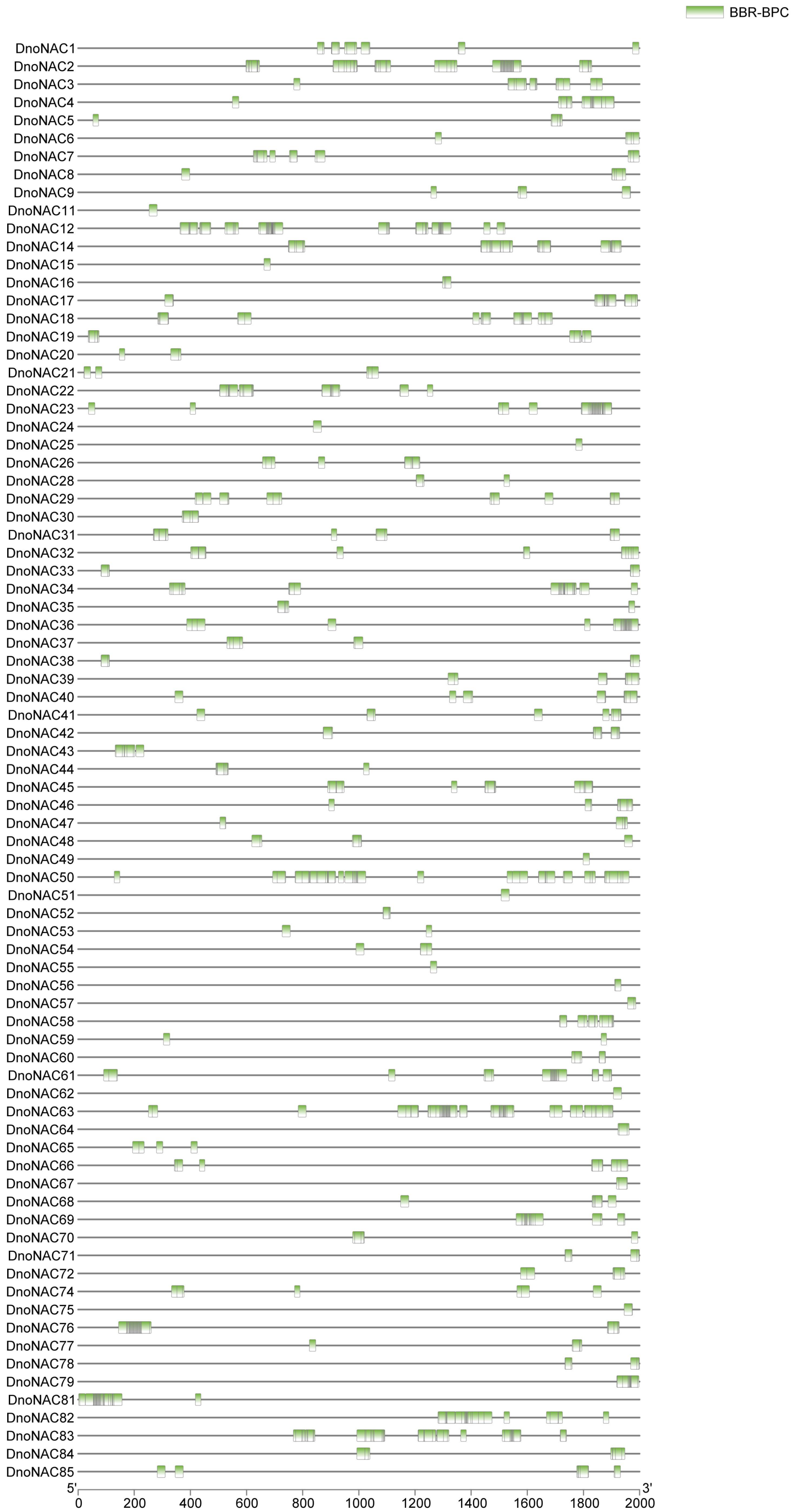

Supplementary Fig. 4 The BBR-BPC TF binding sites in the promoter region of the DnoNACs

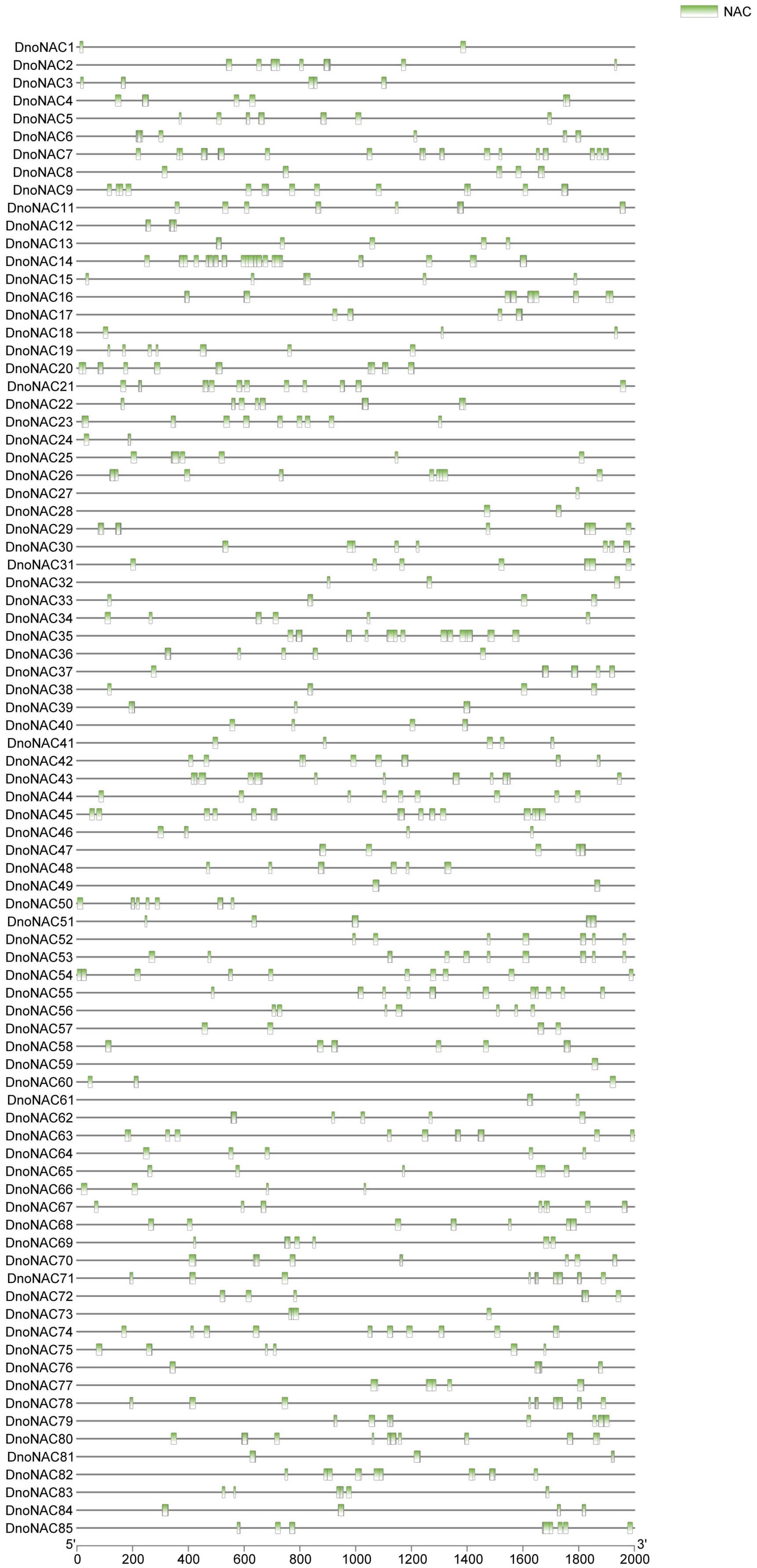

Supplementary Fig. 5. The NAC TF binding sites in the promoter region of the DnoNACs

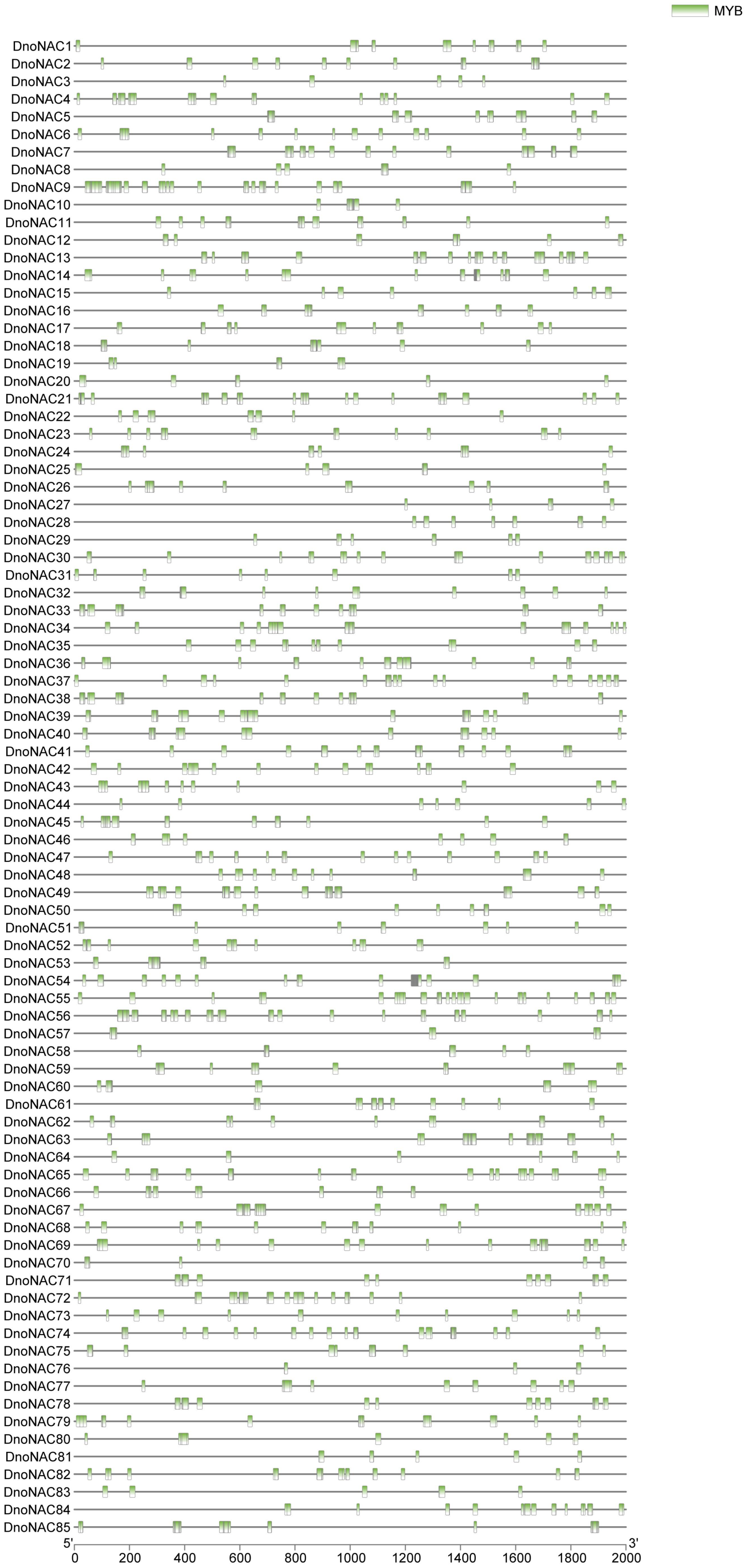

Supplementary Fig. 6. The MYB TF binding sites in the promoter region of the DnoNACs

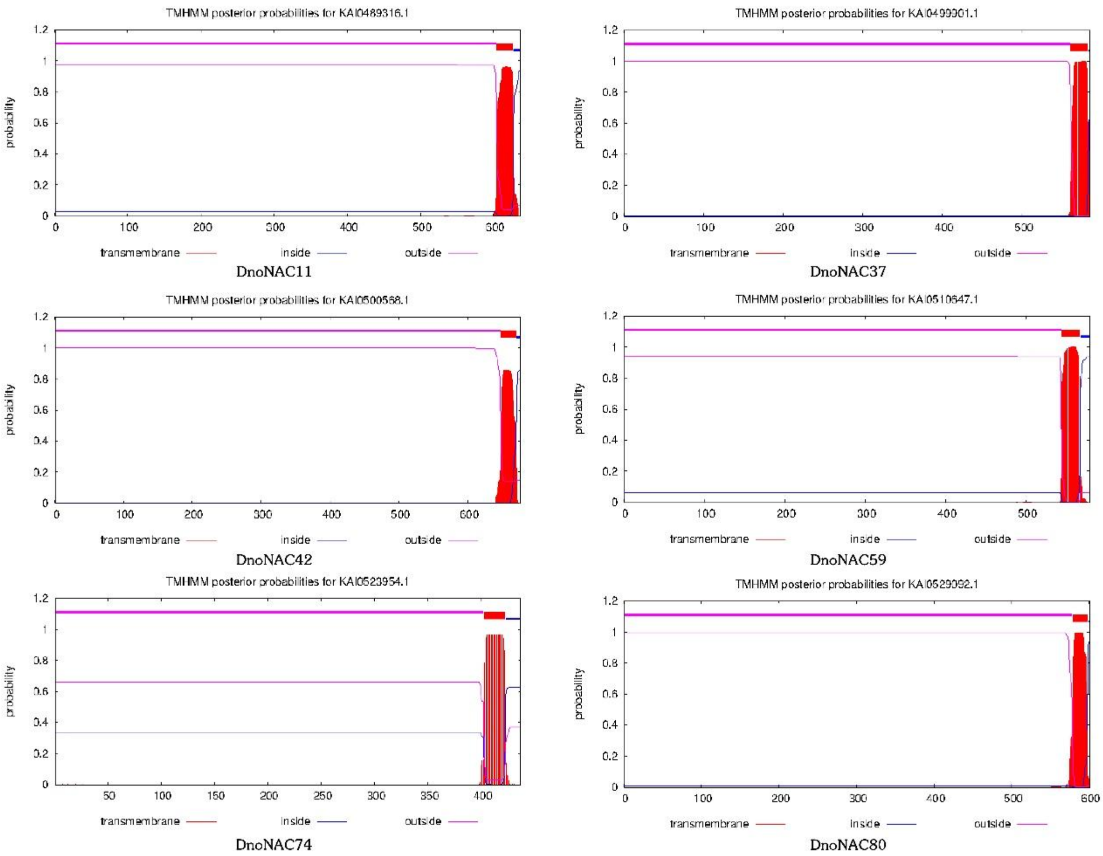

Supplementary Fig. 7. Transmembrane structure prediction of NAC protein from *Dendrobium nobile*

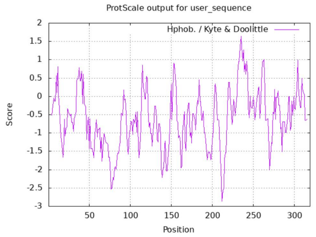

DnoNAC1

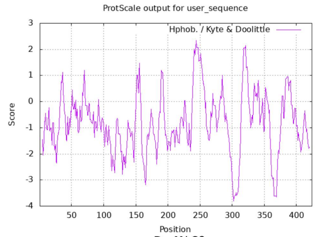

DnoNAC2

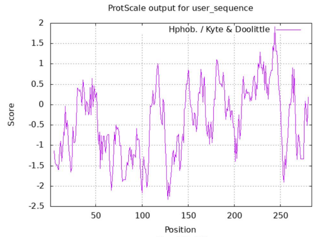

DnoNAC3

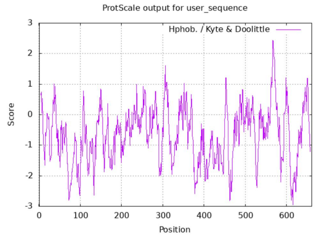

DnoNAC4

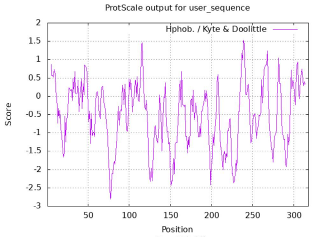

DnoNAC5

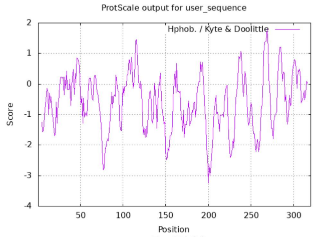

DnoNAC6

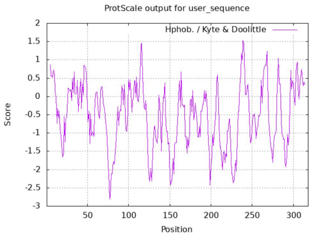

DnoNAC7

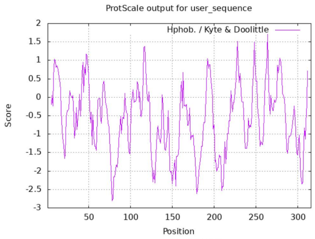

DnoNAC8

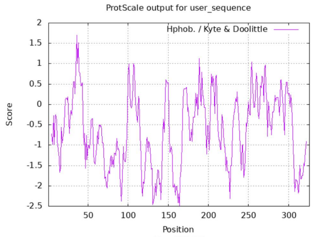

DnoNAC9

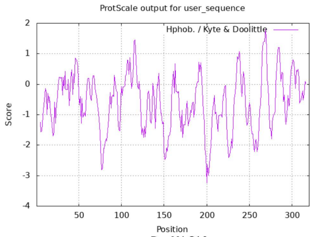

DnoNAC10

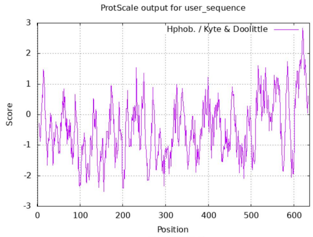

DnoNAC11

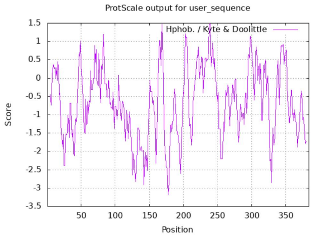

DnoNAC12

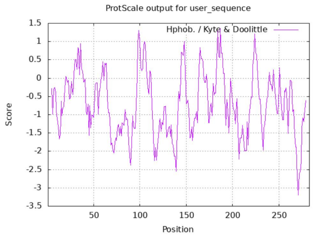

DnoNAC13

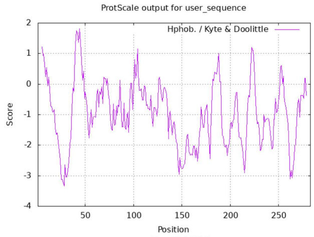

DnoNAC14

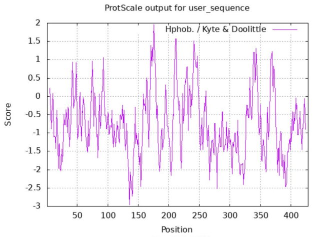

DnoNAC15

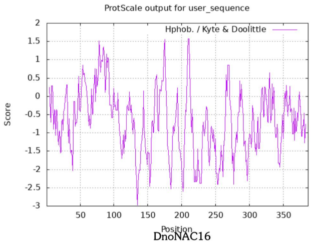

DnoNAC16

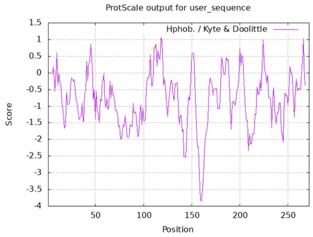

DnoNAC17

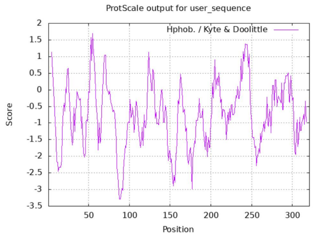

DnoNAC18

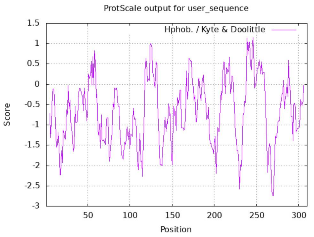

DnoNAC19

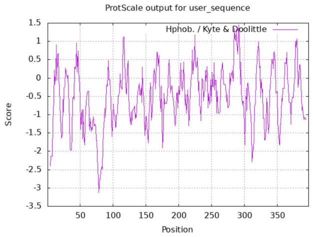

DnoNAC20

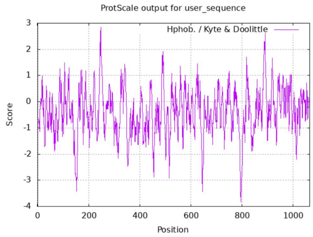

DnoNAC21

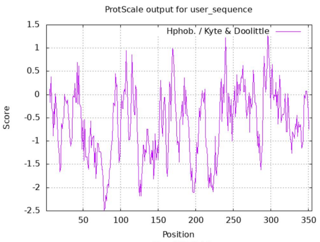

DnoNAC43

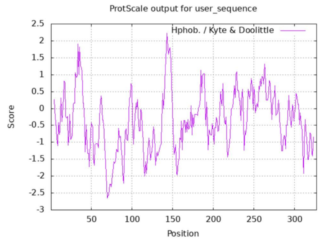

DnoNAC44

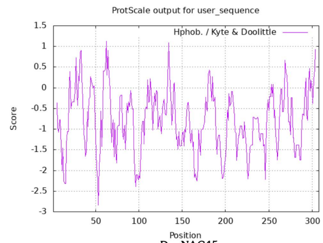

DnoNAC45

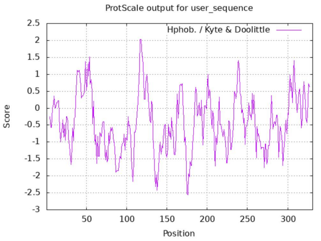

DnoNAC46

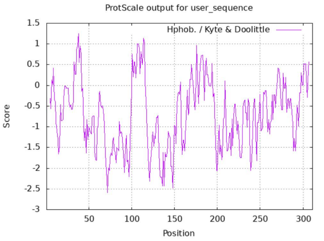

DnoNAC47

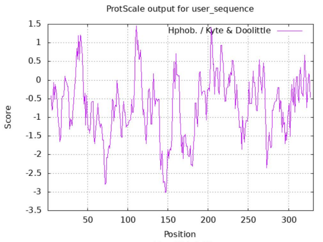

DnoNAC48

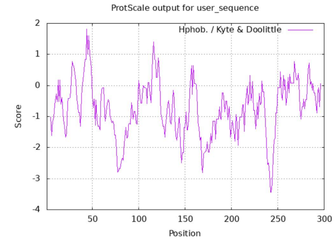

DnoNAC49

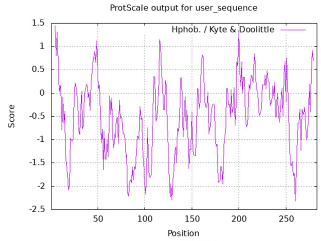

DnoNAC50

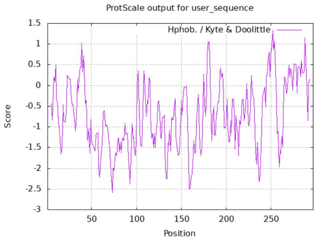

DnoNAC51

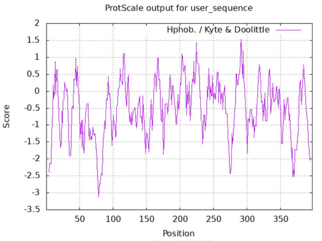

DnoNAC52

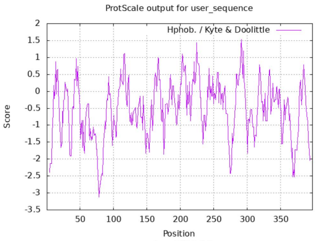

DnoNAC53

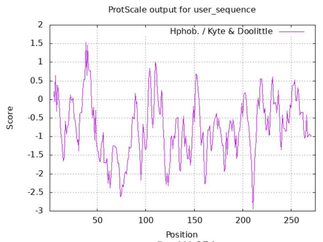

DnoNAC54

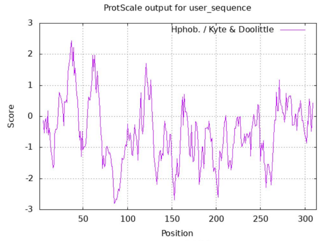

DnoNAC55

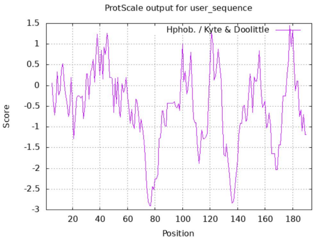

DnoNAC56

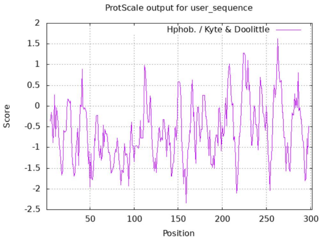

DnoNAC57

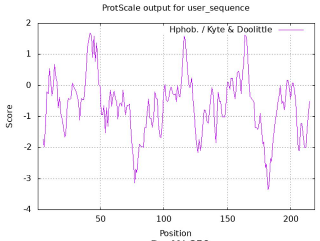

DnoNAC58

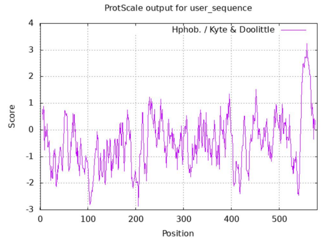

DnoNAC59

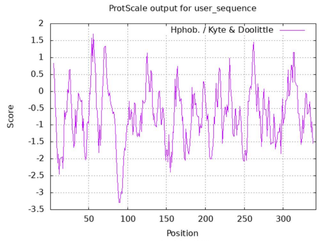

DnoNAC60

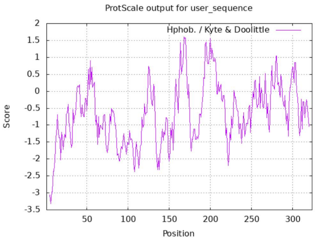

DnoNAC61

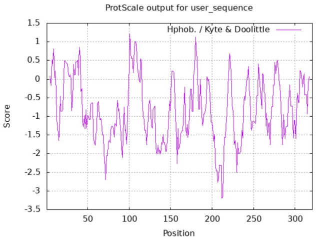

DnoNAC62

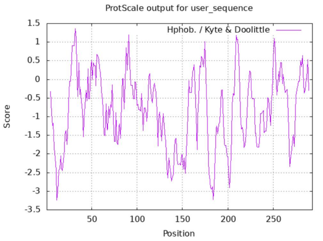

DnoNAC63

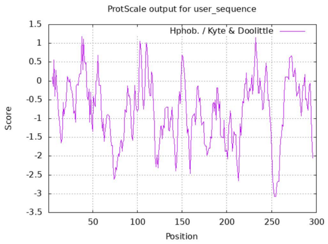

DnoNAC22

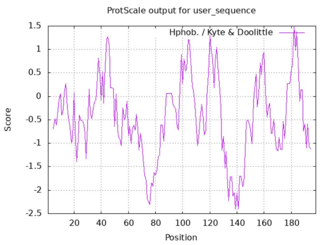

DnoNAC23

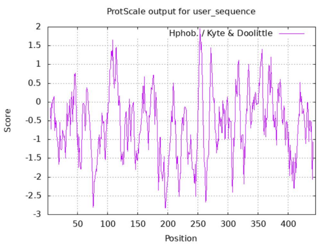

DnoNAC24

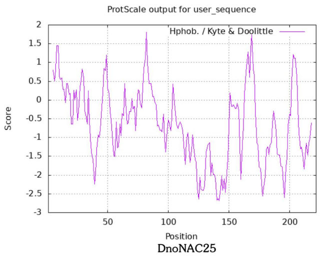

DnoNAC25

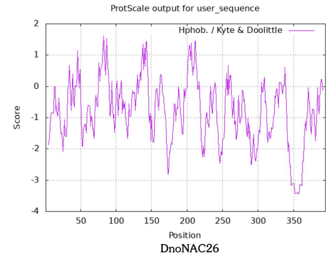

DnoNAC26

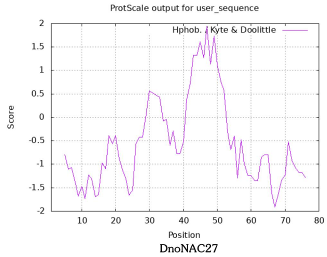

DnoNAC27

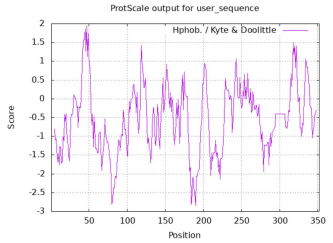

DnoNAC28

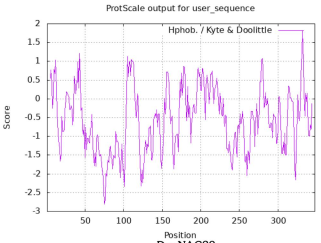

DnoNAC29

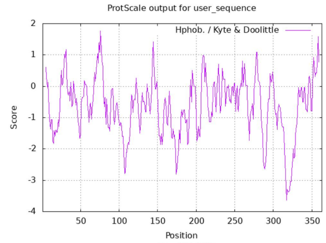

DnoNAC30

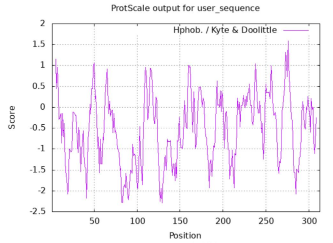

DnoNAC31

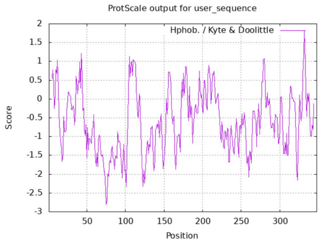

DnoNAC32

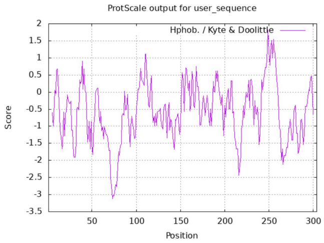

DnoNAC33

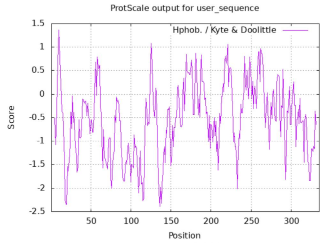

DnoNAC34

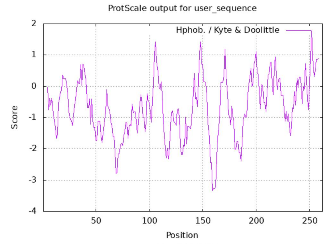

DnoNAC35

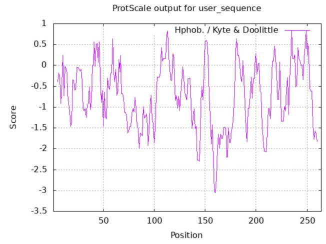

DnoNAC36

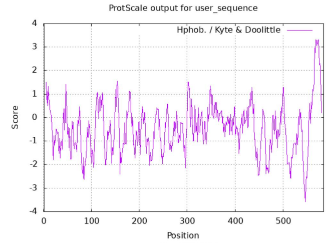

DnoNAC37

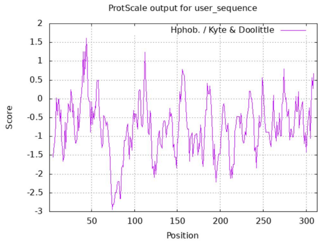

DnoNAC38

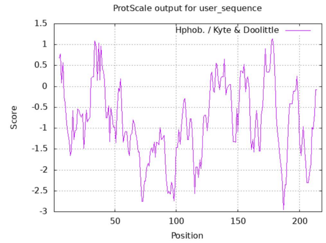

DnoNAC39

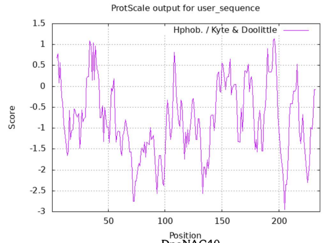

DnoNAC40

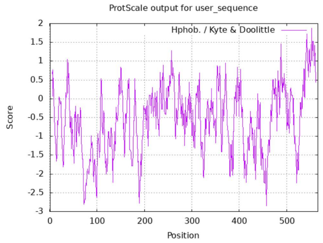

DnoNAC41

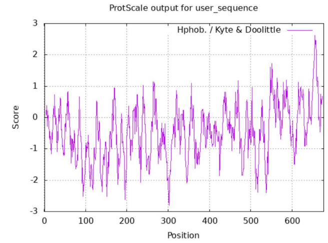

DnoNAC42

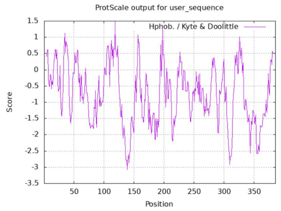

DnoNAC64

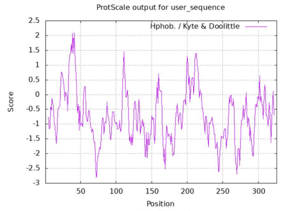

DnoNAC65

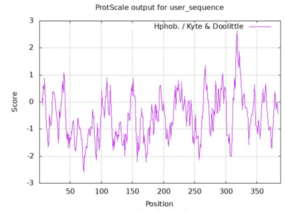

DnoNAC66

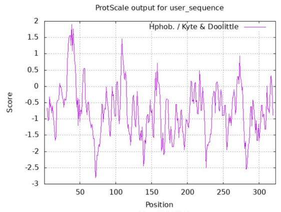

DnoNAC67

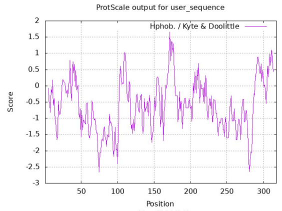

DnoNAC68

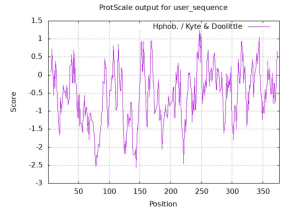

DnoNAC69

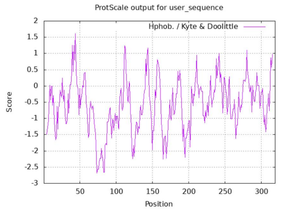

DnoNAC70

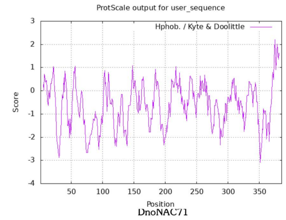

DnoNAC71

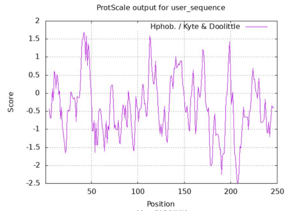

DnoNAC72

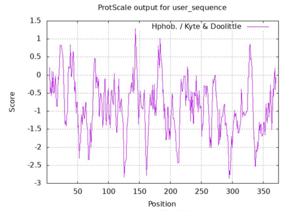

DnoNAC73

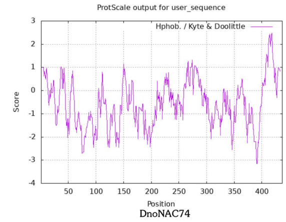

DnoNAC74

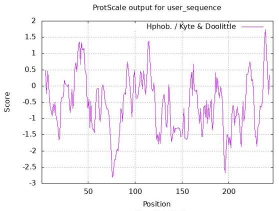

DnoNAC75

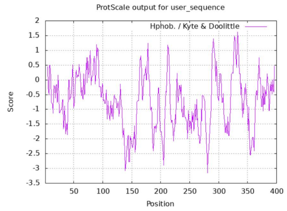

DnoNAC76

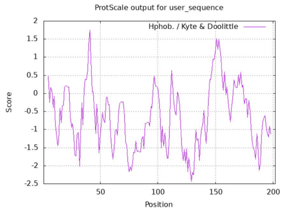

DnoNAC77

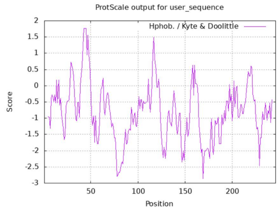

DnoNAC78

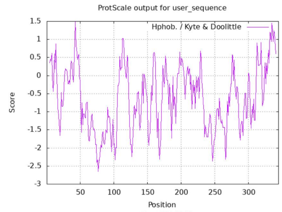

DnoNAC79

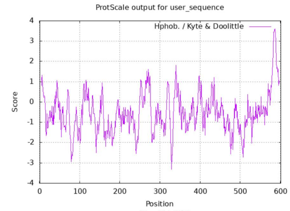

DnoNAC80

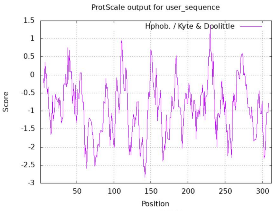

DnoNAC81

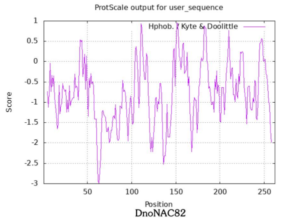

DnoNAC82

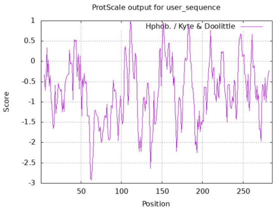

DnoNAC83

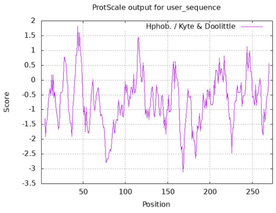

DnoNAC84

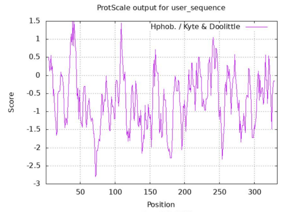

DnoNAC85

Supplementary Fig. 8 Hydrophilicity/hydrophobicity analysis of NAC proteins in *Dendrobium nobile*

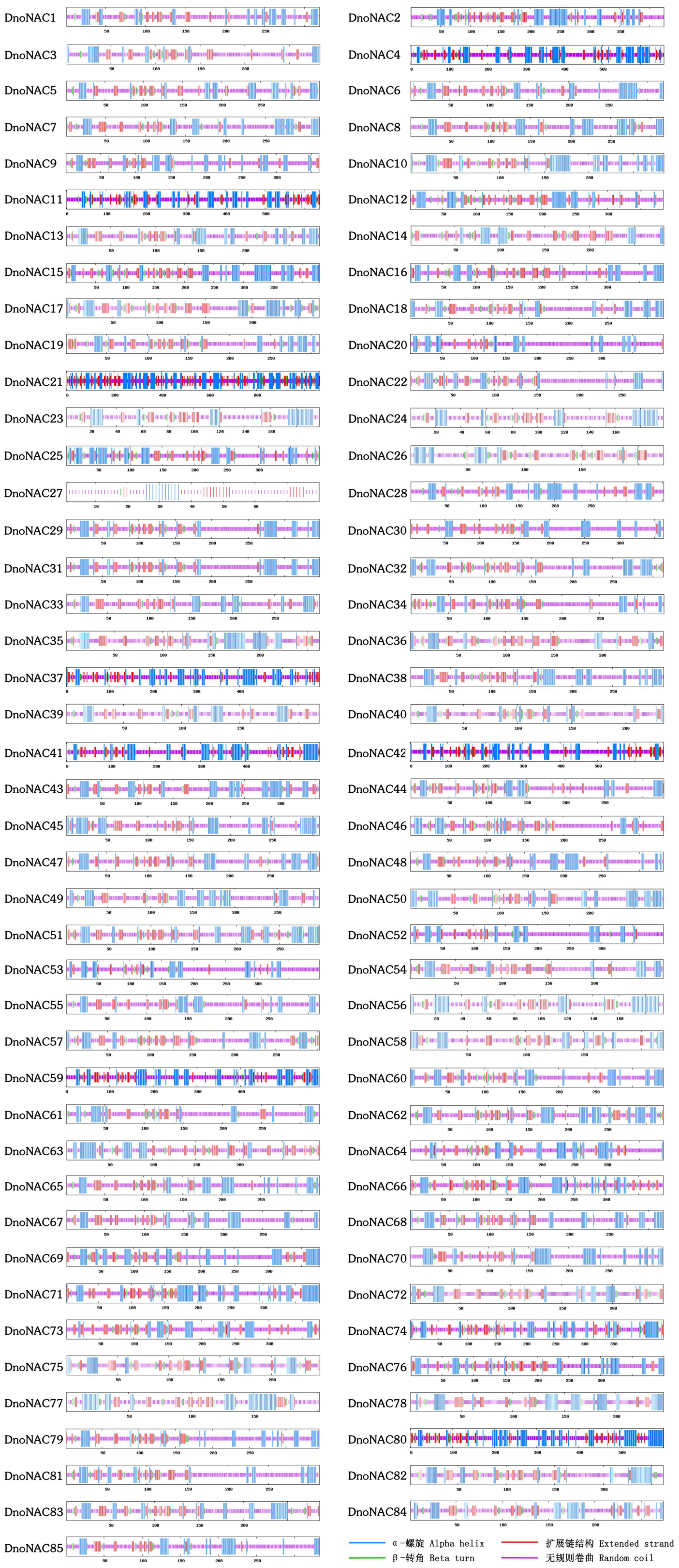

Supplementary Fig. 9. Secondary structure of *NAC* family in *Dendrobium nobile*

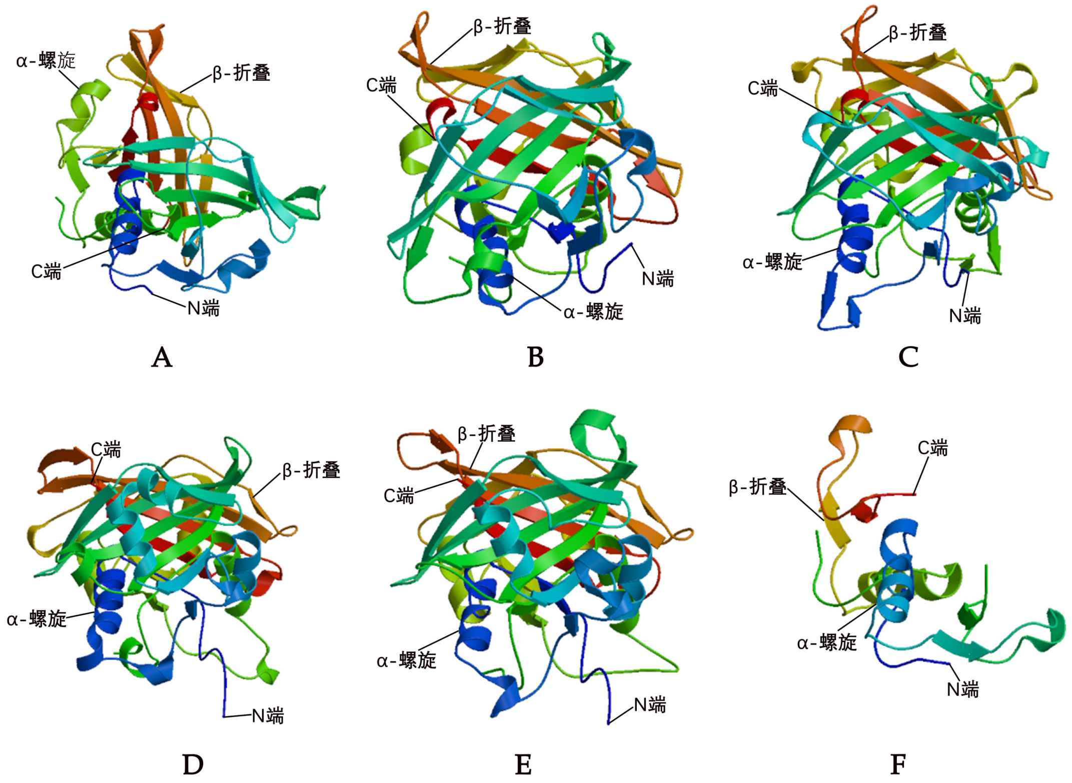

A-DnoNAC01；B-DnoNAC03；C-DnoNAC12；
D-DnoNAC14；E-DnoNAC21；F-DnoNAC27；

Supplementary Fig. 10. The tertiary structure of NAC proteins in *Dendrobium nobile*
